# Historical RNA expression profiles from the extinct Tasmanian tiger

**DOI:** 10.1101/gr.277663.123

**Published:** 2023-08

**Authors:** Emilio Mármol-Sánchez, Bastian Fromm, Nikolay Oskolkov, Zoé Pochon, Panagiotis Kalogeropoulos, Eli Eriksson, Inna Biryukova, Vaishnovi Sekar, Erik Ersmark, Björn Andersson, Love Dalén, Marc R. Friedländer

**Affiliations:** 1 Department of Molecular Biosciences, The Wenner-Gren Institute, Science for Life Laboratory, Stockholm University, 114 18 Stockholm, Sweden;; 2 Centre for Palaeogenetics, 106 91 Stockholm, Sweden;; 3 The Arctic University Museum of Norway, UiT - The Arctic University of Norway, 9006 Tromsø, Norway;; 4 Department of Biology, National Bioinformatics Infrastructure Sweden, Science for Life Laboratory, Lund University, 223 62 Lund, Sweden;; 5 Department of Archaeology and Classical Studies, Stockholm University, 106 91 Stockholm, Sweden;; 6 Department of Bioinformatics and Genetics, Swedish Museum of Natural History, 104 05 Stockholm, Sweden;; 7 Department of Cell and Molecular Biology (CMB), Karolinska Institute, 171 77 Stockholm, Sweden;; 8 Department of Zoology, Stockholm University, 106 91 Stockholm, Sweden

## Abstract

Paleogenomics continues to yield valuable insights into the evolution, population dynamics, and ecology of our ancestors and other extinct species. However, DNA sequencing cannot reveal tissue-specific gene expression, cellular identity, or gene regulation, which are only attainable at the transcriptional level. Pioneering studies have shown that useful RNA can be extracted from ancient specimens preserved in permafrost and historical skins from extant canids, but no attempts have been made so far on extinct species. We extract, sequence, and analyze historical RNA from muscle and skin tissue of a ∼130-year-old Tasmanian tiger (*Thylacinus cynocephalus*) preserved in desiccation at room temperature in a museum collection. The transcriptional profiles closely resemble those of extant species, revealing specific anatomical features such as slow muscle fibers or blood infiltration. Metatranscriptomic analysis, RNA damage, tissue-specific RNA profiles, and expression hotspots genome-wide further confirm the thylacine origin of the sequences. RNA sequences are used to improve protein-coding and noncoding annotations, evidencing missing exonic loci and the location of ribosomal RNA genes while increasing the number of annotated thylacine microRNAs from 62 to 325. We discover a thylacine-specific microRNA isoform that could not have been confirmed without RNA evidence. Finally, we detect traces of RNA viruses, suggesting the possibility of profiling viral evolution. Our results represent the first successful attempt to obtain transcriptional profiles from an extinct animal species, providing thought-to-be-lost information on gene expression dynamics. These findings hold promising implications for the study of RNA molecules across the vast collections of natural history museums and from well-preserved permafrost remains.

Over the past decade, high-throughput sequencing techniques have propelled the analysis of ancient DNA (aDNA) molecules, enabling the study of genomes from extinct or extant species that lived up to around 2 million years ago (van der Valk et al. 2021; Kjær et al. 2022) or in more recent times (Feigin et al. 2022). Studies on aDNA, alongside ancient proteins to lesser extent, have facilitated the exploration of evolutionary processes by simply examining the snapshot of time that paleogenomes and paleoproteomes can provide. This has allowed the reconstruction of genomes and ancestral lineages from multiple extinct species from the Pleistocene era, including Neanderthals (Green et al. 2010), woolly mammoths (Palkopoulou et al. 2015), and woolly rhinoceros (Lord et al. 2020), as well as from others that disappeared more recently, such as the quagga (Vilstrup et al. 2013; Jónsson et al. 2014) and the Tasmanian tiger (Feigin et al. 2018, 2022).

Aside from the well-established field of paleogenomics, and the emerging field of paleoproteomics (Hendy et al. 2018), the analysis of ancient RNA, a key molecule between both DNA and proteins across the central dogma of life, remains elusive. Unlike DNA, RNA provides researchers with additional layers of information so far unexplored in extinct species, such as cell and tissue identity, gene regulatory mechanisms, and evidence of the expression of coding and noncoding loci. Indeed, studies focused on ancient and/or historical RNA molecules have not experienced the similar advancements witnessed in aDNA and proteins (Smith and Gilbert 2018). Although a few early and controversial studies indicated the potential presence of RNA sequences in ancient plant seeds (Rollo 1985; Venanzi and Rollo 1990; Rollo et al. 1991) and ice cores (Castello et al. 1999; Zhang et al. 2006), subsequent research reported the recovery of partial and complete genomes from RNA viruses preserved in seeds (Guy 2013; Smith et al. 2014), feces (Ng et al. 2014), and formalin-fixed tissues (Xiao et al. 2013; Worobey et al. 2016; Gryseels et al. 2020; Patrono et al. 2022), as well as partial transcriptomes from plants (Fordyce et al. 2013). However, it was not until 2017 that the first example of metazoan ancient RNAs were detected using qPCR-based methods in mummified cold-preserved remains of a human dating back more than 5000 yr (Keller et al. 2017), and later reproduced through sequencing techniques in humans from medieval times (Shaw et al. 2019). More recently, two additional studies have used sequencing techniques to recover ancient RNA profiles, including messenger RNA (mRNA) and microRNA (miRNA) molecules from preserved tissues of a Late Pleistocene canid and historical wolf skins (Smith et al. 2019; Fromm et al. 2021). The presence of endogenous RNA sequences in extremely well preserved yet ancient specimens and in historical wolf skins showed that, under favorable conditions, RNA molecules could be preserved to the extent of still representing their abundance in the once-living cells of origin. Albeit there are promising results from both early and recent studies on sequencing and analyzing RNA in metazoan specimens of considerable age, there is currently no example of applying a paleotranscriptomics approach to extinct metazoan species.

To address this gap in the emerging field of paleotranscriptomics, we have focused on the renowned and recently extinct Tasmanian tiger (*Thylacinus cynocephalus*), also referred to as the thylacine. Thylacines were the largest carnivorous marsupials across the Holocene (Mitchell et al. 2014) and represented the only surviving species of the Thylacinidae family to survive into the modern era. They belonged to the order Dasyuromorphia and were closely related to the extant families Dasyuridae (including Tasmanian devils, quolls, phascogales, and dunnarts, among others) and Myrmecobiidae (numbats) (Miller et al. 2009; Feigin et al. 2018). These apex marsupials were once widespread all across the Australian region but eventually became restricted to an isolated population on the island of Tasmania ∼3000 years ago (Paddle 2000). Wild thylacines persisted in Tasmania until the early twentieth century, when European colonizers classified them as an agricultural pest and aggressively targeted their remaining populations, leading to their complete extinction. The last known thylacine died in captivity in 1936 at the Beaumaris Zoo in Hobart, Tasmania. Thylacines are particularly important because they exemplify both a recent human-driven extinction event and an evident case of convergent evolution (Newton et al. 2021; Rovinsky et al. 2021).

Despite diverging from placental carnivorous mammals ∼160 million years ago (Bininda-Emonds et al. 2007), thylacines showed striking phenotypic similarities with extant species like those belonging to the Canidae family. This illustrates how species with distinct evolutionary relationships can undergo common selective pressures, resulting in shared adaptations (Losos 2011). Previous studies have used mitochondrial DNA to determine the evolutionary position of thylacines among marsupial mammals (Miller et al. 2009) and have explored their demographic history and genetic diversity by sequencing and assembling their nuclear genome (Feigin et al. 2018, 2022). This invaluable information has provided researchers with unprecedented insights into the biology of this species.

In this study, we present the thylacine as a proof of concept for expanding the field of paleotranscriptomics into the analysis of historical RNA remains in an extinct species for the first time.

## Results

### Recovery, sequencing, and genome-wide mapping of RNA fragments

**Figure 1.**
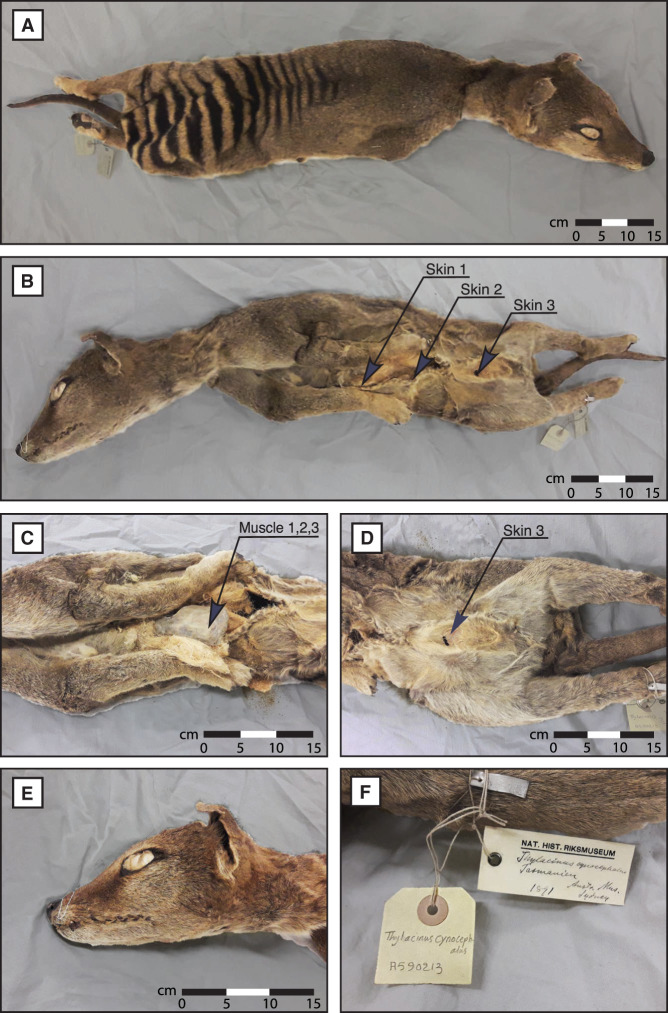
Thylacine specimen NRM-MA590213. (*A*) Dorsal view. (*B*) Ventral view and skin sampling areas. (*C*) Ventral view and skeletal muscle sampling area from the inner side of the left scapula. (*D*) Inguinal region. (*E*) Head view. (*F*) Museum identification.

We first set out to investigate if it is possible to recover useful RNA molecules from a desiccated thylacine specimen stored at room temperature without specific preservative conditions. Three independent samples of both skeletal muscle and skin tissues were obtained by biopsy from a thylacine specimen available at the Stockholm Natural History Museum (NRM-MA590213) (Fig. 1).

**Figure 2.**
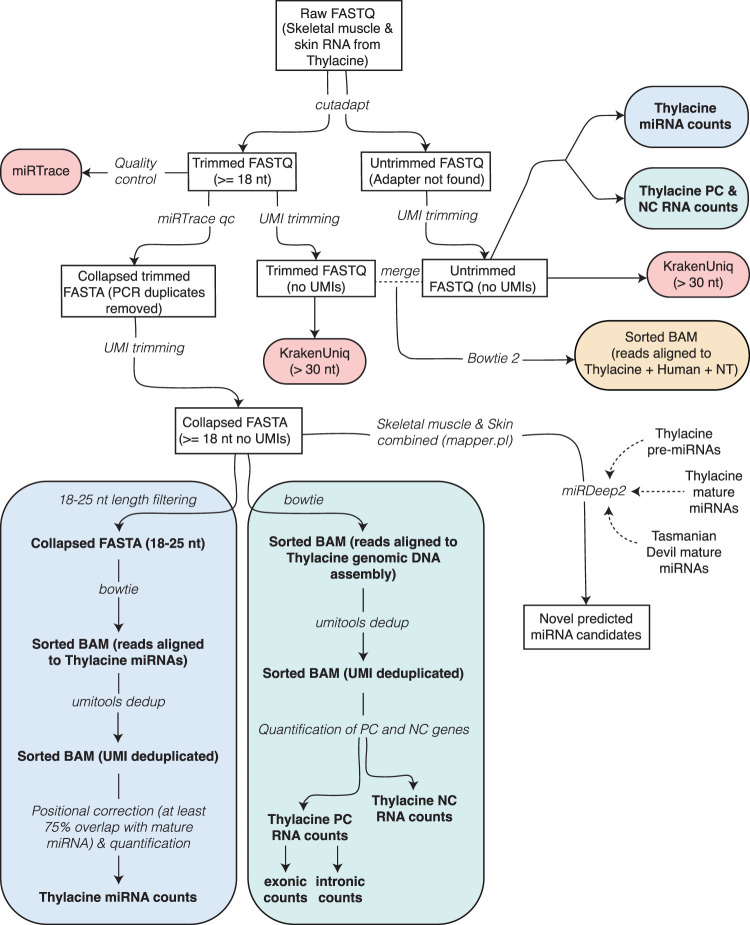
Preprocessing, mapping, metatranscriptomics, and annotation pipeline of skin and skeletal muscle RNA sequences from the NRM-MA590213 thylacine specimen. (NT) Full NCBI nonredundant reference nucleotide database (Pochon et al. 2022); (PC) protein-coding; (NC) noncoding.

Considering the age and preservation status of the specimen, we anticipated a significant fragmentation in the historical RNA sequences possibly present within the tissue matrix. Therefore, we used an RNA extraction protocol targeting small RNA molecules, specifically designed for miRNA sequencing, on each of the six tissue samples obtained (see Methods). The samples were ground in liquid nitrogen and incubated in a digestion buffer to homogenize keratinous hard fibrous tissues (Gilbert et al. 2007; Sinding et al. 2015) while minimizing the incubation time to maximize the RNA extraction yield. From ∼80 mg of tissue per sample, we obtained variable but substantial amounts of total RNA (Supplemental Table 1). Subsequently, the extracted and purified RNA fragments were prepared for high-throughput sequencing using a cDNA library protocol tailored for short RNA transcripts. The library size distribution indicated a successful extraction and library preparation, with an overall length of 150 bp (Supplemental Fig. 1). We sequenced the cDNA libraries using an Illumina NextSeq 500 instrument, generating between 81.9 million and 223.6 million raw sequencing reads per sample (Supplemental Table 2). A computational workflow for processing the RNA data is illustrated in Figure 2 and will be described in subsequent sections. Initially, we trimmed the reads to remove artificial sequencing adapter sequences. Approximately 96% and 94.5% of reads had successful adapter detection and were trimmed (Supplemental Table 2), whereas the remaining ∼5% were kept untrimmed, potentially originating from long RNA transcripts beyond the small RNA sequencing window used. Trimmed sequences <18 nucleotides (nt) were discarded as they were deemed too short for reliable mapping to reference genomes. This is based on the smallest reported size for a functional miRNA transcript (∼20 nt), while allowing up to a 2-nt loss in their 3′ overhangs (Bartel 2018) and avoiding shorter RNA fragments that could probably lead to an excess of unwanted spurious mapping. This step eliminated ∼30% of the trimmed reads, indicating the presence of highly degraded RNA molecules (Supplemental Table 2). PCR duplicates originating from the same RNA molecules were identified and deduplicated based on identical sequences and unique molecular identifiers (UMIs). This procedure reduced the number of sequences to 2.6 million to 12 million per sample, indicating a PCR duplication rate of approximately 21.3 and 11 for skeletal muscle and skin tissues, respectively (Supplemental Table 2). Untrimmed reads showed a lower PCR duplication rate of ∼2.5 and 1.8 for skeletal muscle and skin (Supplemental Table 3), suggesting increased sequence variability compared with that of trimmed reads. The PCR-deduplicated and trimmed reads were then mapped to the thylacine nucleic and mitochondrial genomes, resulting in an overall successful mapping rate of 62.5% in skeletal muscle and 63.3% in skin tissues (Supplemental Table 2).

The read length distribution of mapped trimmed reads showed a predominance of short sequences <30 nt, indicative of time-dependent fragmentation of RNA transcripts from thylacine origin. This pattern was more pronounced in skeletal muscle compared with skin samples (Supplemental Fig. 2A,B). A small increase in reads ranging from 28–35 nt and long reads of 42 nt was also observed (Supplemental Fig. 2A,B). After UMI-based deduplication, we obtained about 1.5 million and 2.8 million mapped trimmed reads for skeletal muscle and skin tissues, with an average UMI deduplication rate of 3.7 and 6.2, respectively (Supplemental Table 2). Most reads were short, although sample 2 from skeletal muscle and sample 1 from skin showed more abundant short reads, indicative of a greater degradation (Supplemental Fig. 2C,D). In contrast, ∼4.5% and 7% of the PCR-deduplicated untrimmed reads were successfully mapped to the thylacine nuclear and mitochondrial genomes in skeletal muscle and skin tissues, respectively, with an average UMI deduplication rate of 3.25 and 9.1 (Supplemental Table 3). This represents an approximately 11-fold decrease in successfully mapped long untrimmed reads compared with short trimmed reads with a similar UMI deduplication rate. Subsequent analyses will primarily focus on short trimmed RNA reads, unless stated otherwise. A detailed analysis of the sequenced RNAs using the miRTrace tool on trimmed and untrimmed reads is available in Supplemental Files 1 and 2, respectively. In summary, we produced millions of stringently quality-controlled sequences from thylacine tissue biopsies.

### Historical RNA sequences show a characteristic damage pattern

The observed damage profiles of RNA reads mapping to the thylacine genome were indicative of the historical nature of the tissues, displaying increased deamination and other nucleotide substitutions (Supplemental Fig. 3). This pattern was more prominent toward the end of the reads, whether they were short (18–25 nt), medium-sized (26–30 nt), or long (>30 nt), in accordance with previous evidence on RNA (Smith et al. 2014, 2019; Fromm et al. 2021) and DNA damage patterns (Dabney et al. 2013). Adenosine deamination to inosine (A > I; read as A > G by the sequencer) was generally less frequent than cytidine to uridine deamination (C > U; read as C > T by the sequencer), except at the 3′ end of short RNA reads (Supplemental Fig. 3). However, distinguishing genuine time-dependent A > I deamination from technical misincorporation is challenging (Gilbert et al. 2003; Binladen et al. 2006). Other types of misincorporations were also prevalent and roughly followed deamination events in RNA reads (Supplemental Fig. 3), which warrants caution about the reliability of some of the observed damage. A similar pattern emerged when examining the sequence damage of untrimmed RNA reads (Supplemental Fig. 4), although samples 2 and 3 from skeletal muscle showed increased damage around the 18th nucleotide, likely owing to the presence of shorter untrimmed reads with undetermined (“N”) nucleotides. This suggests a more pronounced and widespread occurrence of time-dependent damage in RNA compared with DNA sequences, in agreement with the increased degradation susceptibility of RNA molecules relative to DNA. Furthermore, considering that our thylacine specimen has been stored at room temperature for more than a century, DNA preservation might be compromised, and it is highly likely that the same holds true for RNA (Binladen et al. 2006).

### Metatranscriptomic analyses reveal that thylacine-like RNAs are predominant

We aimed to determine the origins of the RNA sequences derived from thylacine tissues and distinguish endogenous reads from additional sources of contamination like other metazoans, microbes, or any other microscopic life. To accomplish this, we used a metatranscriptomic analysis based on the classification of long RNA reads (>30 nt) using KrakenUniq software (Breitwieser et al. 2018) and the complete collection of sequenced organisms in the NCBI nonredundant reference nucleotide (NT) database. This analysis was performed separately on trimmed reads following sequencing adapter identification and on untrimmed reads when sequencing adapters were not detected and were hence left intact (see Methods).

**Figure 3.**
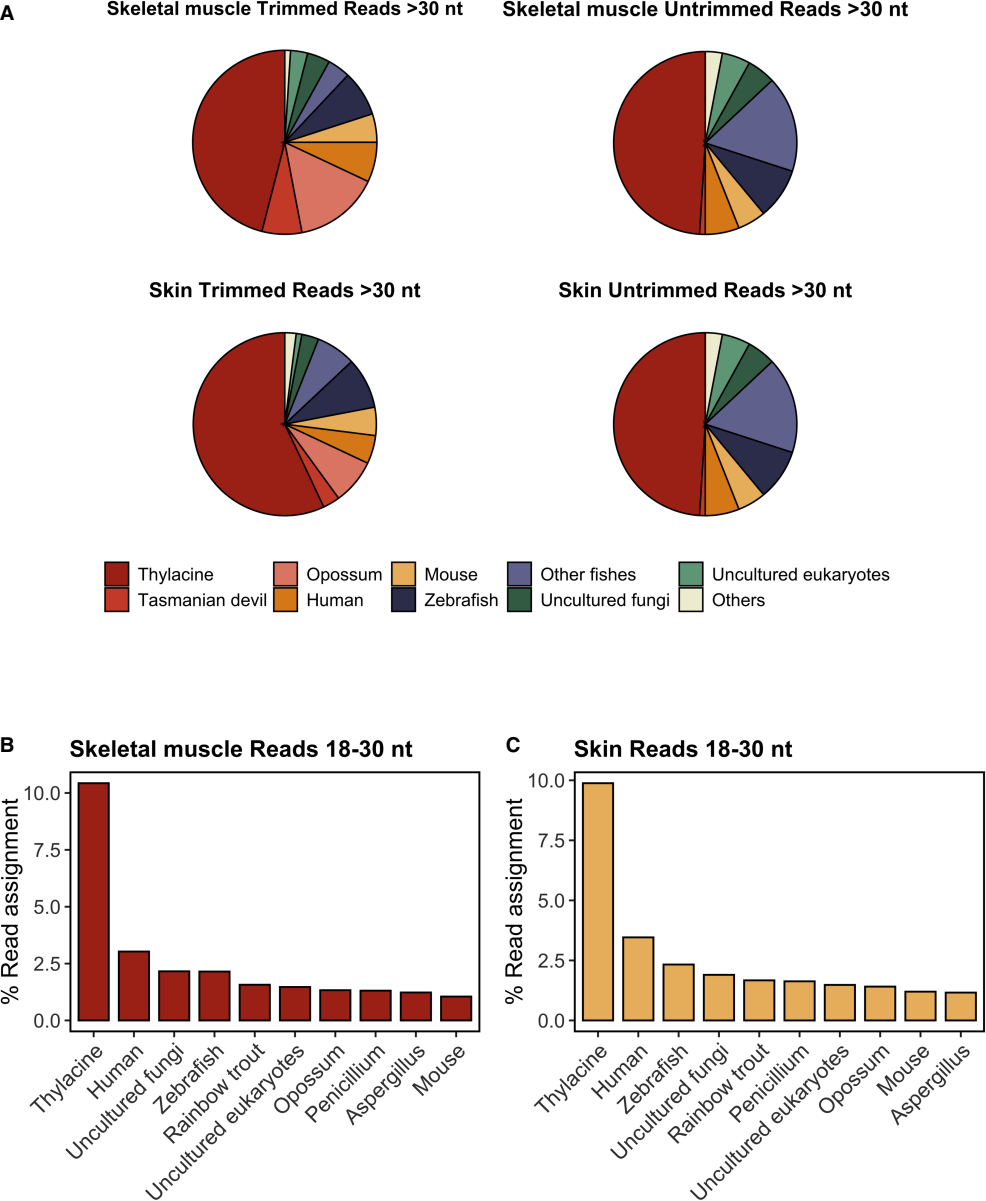
Metatranscriptomic analyses of thylacine skin and skeletal muscle samples. (*A*) Proportion of trimmed and untrimmed RNA reads (>30 nt) assigned using the KrakenUniq pipeline from thylacine skeletal muscle and skin tissues. Only species with more than 1000 *k*-mers and more than 200 species-specific reads are shown. Percentage of RNA reads aligned to the 10 most abundant species detected, focusing specifically on trimmed and untrimmed sequences uniquely mapped (MAPQ ≥ 1) of size ≥18 and ≤30 nt in skeletal muscle (*B*) and skin tissues (*C*). Reads with multiple-species ambiguous mapping were discarded for calculating the percentage of read assignment. Global alignment was performed using Bowtie 2 with flags *‐‐end-to-end* and *‐‐very-sensitive*.

In the case of skeletal muscle, ∼46% of the reliably assigned sequences were attributed to thylacine contigs present in the NCBI NT database (Fig. 3A; Supplemental File 3). Additionally, 15% of the reads were assigned to opossum (*Monodelphis domestica*), and 7% to Tasmanian devil (*Sarcophilus harrisii*), accounting for a total of 68% of reads assigned to these three marsupial species (Fig. 3A; Supplemental File 3). Given the phylogenetic proximity of opossums, Tasmanian devils, and thylacines as members of the Metatheria clade (marsupials), it is plausible that all these sequences indeed originated from the thylacine. Moreover, the probability of cross-contamination from marsupial species other than the thylacine in our data is relatively low. The remaining assigned reads corresponded to human (7%), mouse (5%), undetermined fungi (4%), other eukaryotes (1%), and zebrafish (8%) and other related fish species (4%). These findings were consistent with those obtained for untrimmed skeletal muscle reads (Fig. 3A; Supplemental File 4). The assignment obtained for skin tissue was similar to that of the muscle, although the fraction of sequences that could be reliably traced to the thylacine genome was higher for untrimmed reads (Fig. 3A; Supplemental Files 5, 6). In both skin and muscle samples, the proportion of untrimmed reads attributed to nonthylacine marsupial species was significantly smaller than that of shorter trimmed reads (Fig. 3A). This supports our previous hypothesis that read assignments to nonthylacine marsupial species likely represent misattributed short thylacine sequences. Consequently, reads assigned to other marsupial species might originate from alignments to highly conserved genomic loci across marsupials absent in the thylacine contigs used as reference. This outcome is expected because the thylacine sequences included in the NT database used by KrakenUniq represent only a limited portion of the entire thylacine genome.

We then proceeded to analyze the shorter reads (18–30 nt) that could not be classified with KrakenUniq. Instead, we mapped them directly against a custom reference NT database using a dedicated short-read mapper (see Methods). This additional analysis is crucial because we anticipate that true historical RNAs would be fragmented and therefore shorter in length. Consistent with our findings from the longer reads (>30 nt), the thylacine was the species with the highest proportion of uniquely traceable short reads, surpassing by about threefold the percentage of short reads unambiguously assigned to the most prevalent contaminant source detected, humans, in both skeletal muscle and skin tissues (Fig. 3B,C). This provides further support for the robustness of our analyses and confirms that the thylacine is indeed the primary source of our RNA sequencing data.

There were very few RNA reads assigned to prokaryotes or viral species, and their abundance profiles differed between muscle and skin tissues. The most abundant prokaryotic taxa were uncultured bacteria, followed by *Escherichia*, *Acinetobacter*, *Myroides*, *Streptomyces*, *Rheinheimera*, and *Corynebacterium*. These bacteria are typically associated with environmental contamination (Supplemental Table 4). Regarding viruses, the assigned RNA reads mostly belonged to fungi-specific viruses such as *Penicillium aurantiogriseum partitivirus 1* or *Primate T-lymphotropic virus 1*, potentially derived from human contamination (Supplemental Table 5). A small number of sequences from RNA viruses of unknown origin (picorna-like) were present, indicating that such viruses can be detected. However, these RNA viruses were shallowly supported by reads mapping to only few distinct positions in their genomes. Therefore, additional confirmation is required.

We also investigated an unexpected enrichment of reads assigned to fish-related species (Fig. 3A), primarily to zebrafish (*Danio rerio*). We discovered that most of the sequences successfully aligned to the zebrafish genome (∼80%) mapped to specific regions of its Chromosome 4, which contains numerous repetitive transfer RNAs (tRNAs; accounting for 50% of mapped reads) and ribosomal RNAs (rRNAs; the remaining 30%) (Howe et al. 2013). Because of the high sequence conservation of these loci across species and because of the extensive presence of multiple fish species in the NT database used for *k*-mers classification, we believe that these sequences were falsely assigned to the zebrafish genome. Supporting this observation, we did not observe any enrichment of fish-related assigned reads in the skin compared with muscle tissue, as would be expected if there was a physical contamination from the environment, which is more likely to be present on the skin than deep within the muscle tissue. These findings highlight the need for caution regarding the reliability of nonendogenous contaminating sources detected by our metatranscriptomic pipeline.

One potential explanation for some of the reads assigned to nonthylacine genomes might be a technical bias in our NCBI NT database. Because humans, mice, and zebrafish are model organisms, their reference contigs may be overrepresented. Consequently, a significant fraction of reads, not necessarily of thylacine origin, might be falsely assigned to these species by default. Supporting this hypothesis, when we realigned the reads classified as zebrafish, human, and mouse contamination to the thylacine, Tasmanian devil, and opossum assemblies, a considerable fraction of them were successfully mapped (average mapping rate of 85.74% to thylacine, 85.04% to opossum, and 70.71% to Tasmanian devil). Therefore, it would be premature to dismiss the possibility of an endogenous thylacine origin for at least some of the reads assigned to other nonmarsupial species.

In summary, we consistently observe a predominance of sequences originating from the thylacine genome using different complementary computational approaches, thus supporting the authenticity of the historical RNA sequences.

### Historical RNAs map to numerous classes of protein-coding and noncoding thylacine genes

**Figure 4.**
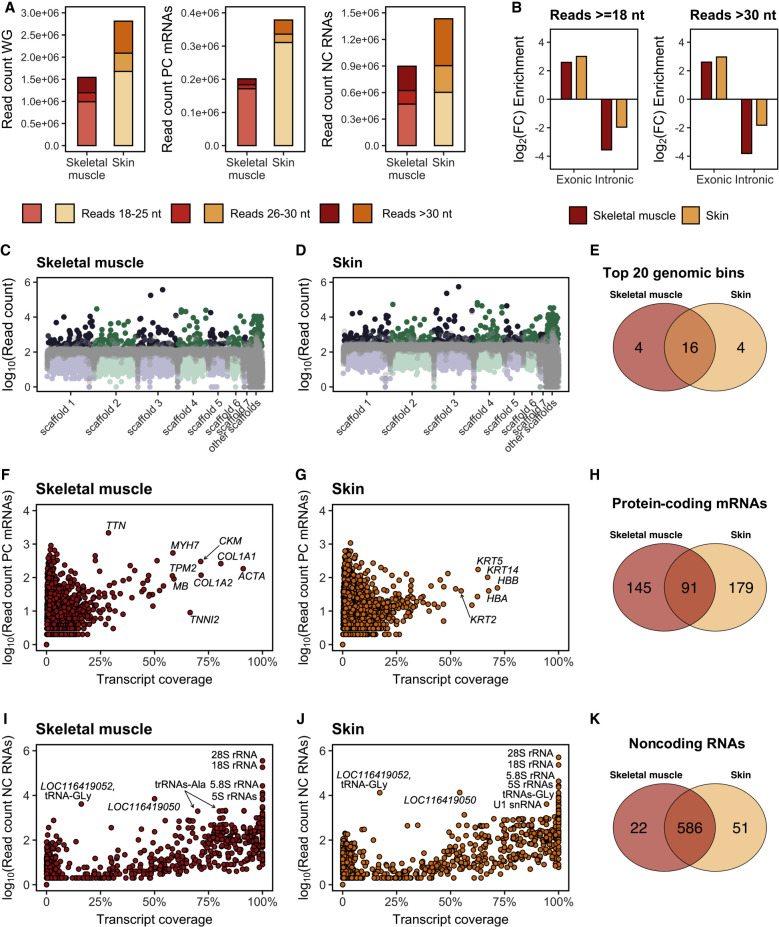
Distribution of RNA sequences over protein-coding and noncoding genes. (*A*) Read length distribution of RNA sequences mapped to the thylacine whole-genome (WG) assembly, annotated protein-coding (PC) genes (N = 19,356) and noncoding (NC) RNA genes (N = 3613). (*B*) Exonic enrichment and intronic depletion of RNA reads mapped to exonic and intronic regions of PC genes (at least 10% coverage) in skeletal muscle (N = 236) and skin (N = 270). Thylacine historical DNA reads mapped to the same PC genes were used as reference for comparison. (*C*,*D*) Number of RNA reads mapped to each consecutive 250-kb window genome-wide in skeletal muscle and skin, respectively. Thylacine DNA reads (SRR5055304) mapped to each consecutive 250-kb window genome-wide are in gray. (*E*) Venn diagram showing the top 20 genomic windows (250 kb) with the highest number of RNA reads mapped in skeletal muscle and skin. (*F*) Number of RNA reads mapped and coverage of each annotated PC gene (N = 19,356) in skeletal muscle. (*G*) Number of RNA reads mapped and coverage of each annotated PC gene (N = 19,356) in skin. (*H*) Venn diagram showing PC genes quantified (at least 10% coverage) in skeletal muscle and skin. (*I*) Number of RNA reads mapped and coverage of each annotated NC RNA gene in skeletal muscle (N = 3613). (*J*) Number of RNA reads mapped and coverage of each annotated NC RNA gene in skin (N = 3613). (*K*) Venn diagram showing NC RNA genes quantified (at least 10% coverage, N = 608 for skeletal muscle and N = 637 for skin) in skeletal muscle and skin.

To understand the molecular nature of the sequenced RNAs, we traced them back to the nuclear and mitochondrial genomes of the thylacine (see Methods). Most reads mapped to noncoding RNA genes, whereas a smaller proportion mapped to protein-coding genes (Fig. 4; Supplemental Table 6), in agreement with the abundance of noncoding RNAs in the eukaryotic cell transcriptome. These noncoding RNAs primarily originated from highly expressed rRNA and tRNA loci. The majority of reads mapped to protein-coding genes were short, indicating age-related fragmentation (90% were <30 nt), whereas fewer reads mapping to noncoding RNA genes were <30 nt (69% in muscle and 62% in skin).

The incorporation of reads >30 nt is widely accepted as the lower threshold for aDNA data to avoid spurious alignments (de Filippo et al. 2018). Previous research (Smith et al. 2019) and our results show the usefulness of including ultrashort reads (18–30 nt) in obtaining comprehensive RNA abundance profiles. When mapping RNA reads to noncoding genes, the majority were assigned to rRNA genes (60% and 68% for skeletal muscle and skin tissues, respectively), followed by tRNA genes, with approximately half the hits. Other noncoding RNA genes, such as long noncoding RNAs (lncRNAs), small nucleolar RNAs (snoRNAs), and small nuclear RNAs (snRNAs) only accounted for 3% of the RNA reads in skeletal muscle and 7% in skin (Supplemental Table 6). This pattern was consistent across all samples except for skeletal muscle sample 3 and skin sample 1, which had a relatively low abundance of rRNAs (Supplemental Table 6). The length of mapped RNA reads varied among the three most abundant loci types (rRNAs, tRNAs, and protein-coding mRNAs) (Supplemental Fig. 5). Protein-coding mRNAs predominantly gathered ultrashort reads <25 nt in both skeletal muscle and skin tissues (Supplemental Fig. 5A,B), whereas rRNA loci showed a higher proportion of longer reads (Supplemental Fig. 5C,D). Reads ranging from 28–35 nt mapped to tRNA loci were particularly prevalent, especially in skeletal muscle 3 (Supplemental Fig. 5E,F). The latter is interesting because tRNA-derived small RNA fragments are known to have sizes compatible with such enrichment (Liu et al. 2022). Additionally, untrimmed RNA reads mapped to the thylacine assembly displayed a similar pattern to that of trimmed reads, although the overall number of successfully mapped reads was significantly lower (approximately 22-fold and 27-fold fewer untrimmed reads compared with trimmed reads in skeletal muscle and skin, respectively) (Supplemental Table 7). Similar to trimmed reads, the dominant loci in untrimmed reads were rRNAs, accounting for an average of ∼74% of the mapped reads, followed by protein-coding mRNAs and tRNAs at a considerable distance (Supplemental Table 7).

We further investigated an unexpected enrichment in long trimmed reads of 42 nt mapped to the thylacine assembly (∼5% over all mapped trimmed reads) (Supplemental Fig. 5; Supplemental Table 8) by cross-species comparison with the most probable source of exogenous modern contamination, humans. Approximately 55% of them mapped to thylacine rRNAs (Supplemental Table 8), which might explain, at least partially, the increased number of long reads >30 nt assigned to noncoding loci, as previously shown in Figure 4A. We then compared the damage profiles of 42-nt reads that indistinctly mapped to the thylacine or the human assemblies (∼57.5%, suspicious of human origin) with those that mapped to the thylacine but failed to align to the human assembly (Supplemental Table 8). Indeed, we observed an increased C > U deamination profile for the 42-nt reads that mapped to the thylacine assembly but failed for the human, whereas those reads that mapped to both thylacine and human assemblies were less damaged (Supplemental Fig. 6). This supports our suspicion that a relevant proportion of these long enriched 42-nt sequences (up to ∼60%) might have an exogenous origin (derived either from humans or from another modern unknown contamination source).

In summary, we find that numerous RNA fragments can be reliably traced to thylacine protein-coding and noncoding genes.

### RNA sequences are enriched in exonic regions and span exon–exon junctions

We investigated whether RNA reads mapped to protein-coding genes were concentrated on exonic regions compared with intronic regions. This is important because potential DNA contamination traces could perturb our analyses when working with sparse amounts of highly fragmented historical RNA material. Sequencing mature mRNA molecules after splicing should show an enrichment of reads mapped to exonic regions and few reads mapped to intronic regions, as opposed to the even distribution expected for DNA sequencing data. The analysis of RNA reads mapped to protein-coding loci with reliable breadth of coverage (>10%) revealed 93% and 77% of reads mapping to exonic regions in skeletal muscle and skin tissue, respectively. This finding agrees well with previous paleotranscriptomic analyses in a Pleistocene canid (Smith et al. 2019) and indicates a significant enrichment in exonic reads compared with what was observed with thylacine DNA sequences (15% and 10% exonic reads in skeletal muscle and skin) (Fig. 4; Supplemental Table 9). Conversely, RNA reads mapped to intronic regions showed a significant depletion in skeletal muscle (12-fold) and skin tissues (fourfold) compared with DNA reads mapped to introns (Fig. 4B).

We also aimed to investigate the presence of RNA reads spanning exon–exon junctions, indicative of mature intron-less mRNAs rather than nascent transcripts or DNA contamination. Approximately 1% of the RNA reads mapped to exonic regions across all annotated protein-coding loci spanned exon–exon junctions (Supplemental Table 10). In contrast, only ∼0.25% of the RNA reads mapped to intronic regions spanned exon–intron junctions, confirming that we primarily detected mature cytoplasmatic transcripts (Supplemental Table 10).

In summary, we provide evidence of the sequencing of RNA fragments from mature cytoplasmic mRNAs that are enriched in exonic sequences and span exon–exon junctions.

### RNA sequences map unevenly and show evidence of unannotated loci

Contrary to aDNA sequences, we expect that ancient/historical RNA fragments would map unevenly across the thylacine genome, representing variations in gene expression from distinct loci. We found that the breadth of coverage for the thylacine genome is 0.17% in skeletal muscle and 0.32% in skin. Moreover, the average depth genome-wide was ∼0.008× for skeletal muscle and ∼0.015× for skin (Supplemental Table 11). When analyzing thylacine DNA data (Feigin et al. 2018) at an equivalent sequencing depth, the observed breadth of coverage genome-wide was 78.35%, with an average depth of 3.45× (Supplemental Table 11).

To investigate the variation in transcriptional depth throughout the genome, we aggregated reads mapping to consecutive nonoverlapping genomic windows of 250 kb. This analysis revealed several genome-wide expression hotspots (Fig. 4C,D; Supplemental Fig. 7), including two prominent ones in thylacine Scaffold 3 that consistently displayed higher read counts (Supplemental Table 12). Among the top 20 expression hotspots (Supplemental Table 12), 16 (80%) were shared between skeletal muscle and skin tissues (Fig. 4E), indicating a common pattern of expression hotspots across the genome in both tissues. Although some of these hotspots could be attributed to RNA reads mapping to the 340 annotated tRNAs or the four annotated 5S rRNAs in the thylacine assembly, the majority did not align with any annotated loci. The thylacine gene annotation currently includes only four 5S rRNA genes (Supplemental Table 13). Therefore, we speculated that the missing 18S, 28S, and 5.8S rRNA genes in the thylacine genome might be located within the top two highlighted expression hotspots (Fig. 4C,D), potentially explaining the abundance of RNA reads mapped to these loci. Indeed, ∼70% of the unexplained reads assigned to the expression hotspots in thylacine Scaffold 3 aligned with the Tasmanian devil 18S rRNA gene or the four human reference rRNA genes. Consequently, we determined the probable location of the missing 18S, 28S, and 5.8S rRNA genes in the thylacine genome (Supplemental Table 13; Supplemental Fig. 8).

We further investigated the causes and origin of RNA reads mapping to intergenic regions, which are not expected to produce transcriptional products. Approximately 29% of mapped reads in skeletal muscle and 38% in skin were assigned to intergenic regions (Supplemental Table 6). To assess the potential influence of historical thylacine DNA contamination in these mappings, we compared their distribution across genomic windows with thylacine DNA data (Feigin et al. 2018). Reads mapping to intergenic regions revealed highly expressed unannotated loci, indicating the need for further annotation efforts in the thylacine assembly (Supplemental Fig. 9). Reads mapping to genomic windows with breadth and depth of coverage similar to thylacine DNA reads, within a twofold difference (see Methods), were considered potential DNA contamination candidates. DNA reads are expected to be evenly distributed across the genome, resulting in an increased but roughly constant genome-wide breadth of coverage. In contrast, RNA reads should be concentrated in coding regions, leading to genomic hotspots with high read counts but lower genome-wide breadth of coverage compared with DNA data at an equivalent sequencing depth. After accounting for sequencing depth and read length distribution biases, ∼3.2% of intergenic mapped reads in skeletal muscle and 2.1% in skin showed a distribution resembling that of thylacine DNA data. Thus, a limited presence of endogenous thylacine DNA contamination in our data should not be discarded. The remaining intergenic mapping reads may be attributed, at least partially, to spurious mapping caused by repetitive low-complexity genomic regions and/or unintended cross-species DNA/RNA contamination of unknown origin.

### Thylacine RNA expression profiles reflect cellular and tissue functions

An advantage of RNA sequence data is that they can yield information about gene expression patterns in ancient/historical tissues, a feature that DNA alone cannot provide. We found that approximately 0.2 million skeletal muscle RNA reads mapped to protein-coding genes, whereas the number was even higher for the skin sample, at approximately 0.4 million reads. Among the protein-coding genes quantified in muscle with reliable coverage (>10%; see Methods), several were characteristic of skeletal muscle tissue metabolism and structure (Fig. 4F; Supplemental Table 14). The most abundantly detected protein-coding transcript in the skeletal muscle was titin (*TTN*), which also showed low coverage (28.70%), and this was reproduced in both trimmed and untrimmed reads (Supplemental Table 14). These two observations can be explained by *TTN* being the gene with the longest known coding sequence, producing a giant protein largely abundant in striated muscle and mainly responsible for preventing overstretching of the sarcomere (Labeit and Kolmerer 1995; Lee et al. 2007). To discard the presence of a confounding effect of long transcripts gathering a higher number of mapped reads, leading to higher breadth of coverage, we investigated whether a positive correlation between transcript length and transcript coverage was present. We found a near-neutral correlation between these variables (Supplemental Fig. 10), indicating that transcript abundance is not solely influenced by sequence length. This observation dispels concerns about length-induced biases in transcript abundance and underscores the reliability of our findings.

Other highly expressed and well-covered transcripts in skeletal muscle included the following: *LOC100913894*, which corresponds to a subunit of actin alpha (*ACTA*); *LOC100925998*, corresponding to myosin heavy chain 7 (*MYH7*); myosin heavy chain 2 (*MYH2*); tropomyosin beta (*TPM2*); and troponin I1, slow skeletal type and troponin I2, fast skeletal type (*TNNI1* and *TNNI2*), all of which form integral part of the sarcomere unit of striated muscle cells (Ahmed et al. 2022). Untrimmed reads also showed similar patterns but with reduced resolution (Supplemental Table 14). *MYH7*, *TPM2*, and *TNNI1* transcripts represented the most abundant isoforms in their gene families, indicating the prevalence of type I slow muscle fibers (Bottinelli and Reggiani 2000). This is in agreement with the tentative functionality of the muscle fibers sequenced, which were probably sampled from the slow-twitched subscapularis muscle according to the bone topology from where the muscle tissue was obtained. Creatine kinase, M-type (*CKM*) and myoglobin (*MB*), important for muscle cell metabolism (Johnson et al. 1989; Ordway and Garry 2004), were also highly abundant. Collagen type I alpha 1 chain and collagen type I alpha 2 chain transcripts (*COL1A1*, *COL1A2*), which contribute to the structure of tissues as type I collagen constituents (Henriksen and Karsdal 2019), were abundant as well.

In the skin, prominently expressed and reliably covered (>10%) loci included keratin-derived transcripts such as keratin 14 (*KRT14*) and keratin 2 and 5 (*KRT2* and *KRT5*) consistent with the epithelial nature of the skin tissue (Fig. 4G; Supplemental Table 15). Actin gamma 1 (*ACTG1*), a cytoplasmic actin isoform expressed ubiquitously except in muscle tissues, also showed high coverage (Supplemental Table 15). In addition, hemoglobin alpha and beta subunits were detected, although predominantly in skin samples 2 and 3, suggesting possible blood infiltration in those samples. Comparing the reliably captured protein-coding genes between skeletal muscle (N = 236) and skin (N = 270) revealed a shared subset of only 91 genes (22%) (Fig. 4H; Supplemental Table 16).

Although tissue-specific protein-coding genes were prevalent, noncoding RNA genes displayed a different pattern. The most abundant noncoding RNA transcripts were rRNA genes in both skeletal muscle and skin tissues, followed by tRNAs and snRNAs (Fig. 4I,J; Supplemental Tables 17, 18). Two highly expressed lncRNAs, *LOC116419050* and *LOC116419052*, likely contained reads mapping to overlapping tRNA loci. Among the reliably detected noncoding RNA genes, 586 (89%) were shared between the tissues (Fig. 4K; Supplemental Table 19).

Regarding RNA reads mapping to the thylacine mitochondrial genome, the most abundant transcripts were the 16S and 12S mitochondrial rRNAs. These transcripts showed high breadth of coverage comparable to their nuclear rRNA counterparts in both muscle and skin tissues using trimmed and untrimmed reads (Supplemental Tables 20, 21).

### Expanding the miRNA complement of the thylacine genome

miRNAs are short regulatory molecules involved in post-transcriptional gene expression repression (Bartel 2018). They play crucial roles in various biological processes, including development and cell identity. Our library preparation method allowed us to directly detect full miRNA transcripts, and we used our data to enhance the annotation of thylacine miRNAs. We applied three complementary annotation approaches to obtain a comprehensive complement of thylacine miRNA loci (see Methods), combining the output of MirMachine (Umu et al. 2023), which annotates miRNAs by homology search, and miRMiner (Wheeler et al. 2009), which fits high-throughput sequencing data to a model of miRNA biogenesis, along with homologous sequence search using the opossum (*M. domestica*) and Tasmanian devil (*S. harrisii*) genome assemblies. In this way, we expanded the thylacine miRNA repertoire from 62 to 325 annotated miRNA genes (Supplemental Table 22). Among the previously described hairpins (Feigin et al. 2022), all were detected except for Mir-340 and Mir-497 (Supplemental Table 23). The annotated precursor miRNA sequences in the thylacine genome can be found in Supplemental File 7.

In summary, we increased the number of annotated thylacine miRNA genes by fivefold.

### Thylacine RNA profiles show high tissue specificity

**Figure 5.**
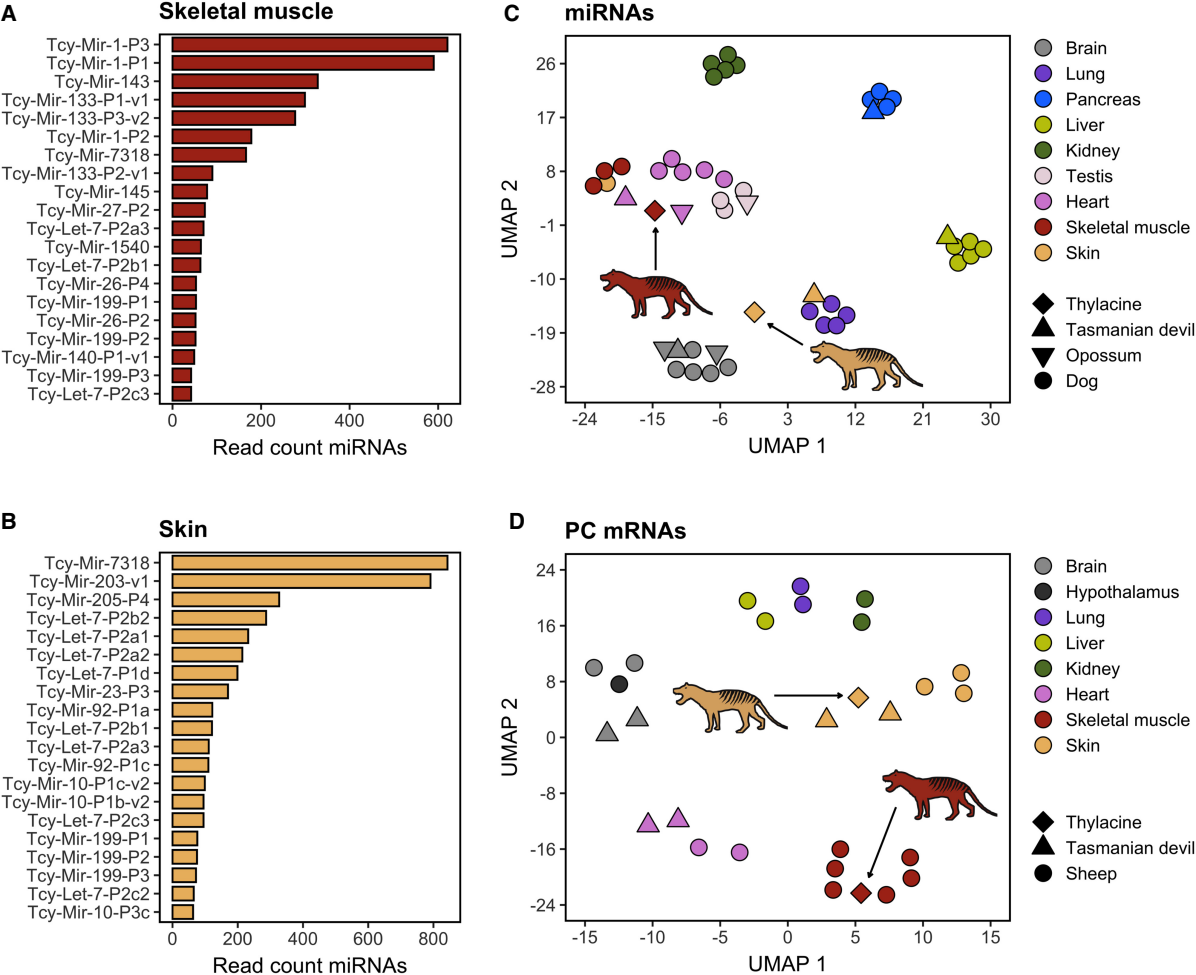
Divergent RNA profiles in thylacine skin and skeletal muscle samples. Number of RNA sequences mapped to the 20 most abundant thylacine miRNA genes profiled in skeletal muscle (*A*) and skin tissue (*B*). (*C*) UMAP embedding depicting diverse tissue samples clustering belonging to dog (circular shape), Tasmanian devil (triangular shape), and opossum (inverted triangular shape) miRNA expression profiles (N = 119) available at MirGeneDB2.1 (Fromm et al. 2022), as well as miRNA profiles of thylacine skeletal muscle and skin tissues (diamond shape). (*D*) UMAP embedding depicting diverse tissue samples clustering belonging to sheep (circular shape) and Tasmanian devil (triangular shape) protein-coding (PC) mRNA expression profiles (N = 261 mRNAs), as well as PC mRNA expression profiles of thylacine skeletal muscle and skin tissues (diamond shape).

To obtain a comprehensive profile of the miRNA complement present in our RNA sequencing data, we focused on reads mapped to the newly annotated thylacine miRNA loci in this study (N = 325) (Supplemental Table 24). In skeletal muscle, we detected and quantified a total of 120 distinct miRNAs, whereas in skin tissues, 143 distinct miRNAs were identified (Supplemental Table 25). Unlike other noncoding RNA genes such as rRNAs and tRNAs, miRNA abundance profiles varied significantly between tissues, with only 35% of the top 20 most abundant miRNAs being shared. In skeletal muscle, the MIR-1, MIR-133, and MIR-143 families were the most abundant (Fig. 5A), and this was observed for both trimmed and, at much more reduced resolution, untrimmed reads (Supplemental Table 25). From these, MIR-1 and MIR-133 are miRNAs at times referred to as myomirs, given their abundance and characteristic muscle-specific functions (Chen et al. 2006; Li et al. 2018; Safa et al. 2020). In contrast, the skin miRNA profile highlighted the abundance of the MIR-7318, MIR-203, MIR-205, and LET-7 families (Fig. 5B; Supplemental Table 25). MIR-203 and MIR-205 are also known to be abundant in the skin (Fromm et al. 2022), contributing to epithelial growth and keratinization (Yi and Fuchs 2010; Viticchiè et al. 2012; Wang et al. 2013; Jiang et al. 2020). We also observed, consistent with miRNA biogenesis dynamics (Bartel 2018), dominant transcripts from either the 5′ or 3′ arms of the precursor molecules (Supplemental Fig. 11; Supplemental Table 26). This shows how even subtle details of miRNA biogenesis are reproduced in our historical RNA expression profiles, and further supports the authenticity of the detected thylacine miRNA molecules.

In addition, we aimed to determine whether the observed divergent miRNA abundance patterns in thylacine skeletal muscle and skin tissue resemble their modern mammalian tissue counterparts. To do so, we used a miRNA-focused tissue expression atlas from the Tasmanian devil (*S. harrisii*), opossum (*M. domestica*), and dog (*Canis familiaris*) species available at MirGeneDB 2.1 (Fromm et al. 2022). It is well established that tissue-specific miRNA expression patterns are conserved across evolution; therefore, we hypothesized that our historical miRNA profiles should cluster according to tissues rather than to their species identity. Only shared miRNAs among the four species included and captured in the thylacine by small RNA sequencing were considered (N = 119) (Supplemental Table 27).

The UMAP dimensionality reduction built using the miRNA tissue expression atlas showed a close relationship in tissue identity (Fig. 5C). The Tasmanian devil, opossum, and dog miRNA profiles largely clustered according to their tissue of origin. The thylacine skeletal muscle grouped closely to the skeletal muscle and heart tissues from dog, as well as to heart muscle samples from Tasmanian devil and opossum. Hence, this is indicative of a conserved miRNA expression profile across species for muscle-related tissues that was preserved and recovered after RNA sequencing of thylacine skeletal muscle. The thylacine skin, however, did not reproduce the same pattern (Fig. 5C), probably owing to the limited collection of skin samples available and to the lack of homogeneous miRNA profiles among the few reference skin samples considered. A similar pattern was reproduced when projecting each thylacine sample from skeletal muscle and skin tissue independently (Supplemental Fig. 12A).

We also attempted to reproduce the tissue clustering analysis using protein-coding mRNA expression profiles with sheep (*Ovis aries*) and Tasmanian devil (*S. harrisii*) tissue atlases, including reliably profiled protein-coding mRNA genes (breadth of coverage >10%), and with shared homologous loci among sheep, Tasmanian devil, and the thylacine (N = 261) (Supplemental Table 27). In this case, both thylacine skeletal muscle and skin samples clustered concordantly with the corresponding sheep and Tasmanian devil muscles and skins, revealing a conserved tissue-specific abundance of protein-coding mRNA transcripts across species, at least for the protein-coding loci considered in our analyses. When attempting to reproduce the tissue clustering with each six independent samples from the thylacine, the overall tissue identity observed with merged samples was preserved (Supplemental Fig. 12B), as previously seen for the embedding using reads mapped to miRNA loci.

In summary, the miRNA and mRNA profiles in historical samples from the extinct thylacine resemble extant modern animal counterparts, supporting the authenticity of the sequences and showing that tissue-specific expression profiles can be preserved in dried museum specimens.

### Discovery of putative novel miRNAs in the thylacine genome

Species-specific miRNAs can only be confirmed through direct sequencing of their RNA molecules. Therefore, RNA sequencing data offer a unique opportunity to identify species-specific miRNA genes from extinct organisms like the thylacine. We used the miRNA discovery algorithm miRDeep2 (Friedländer et al. 2012) to predict novel miRNAs from thylacine skeletal muscle and skin. After careful curation of all miRNA candidates supported by RNA reads in both tissues, two promising loci were selected (Supplemental Tables 28, 29).

**Figure 6.**
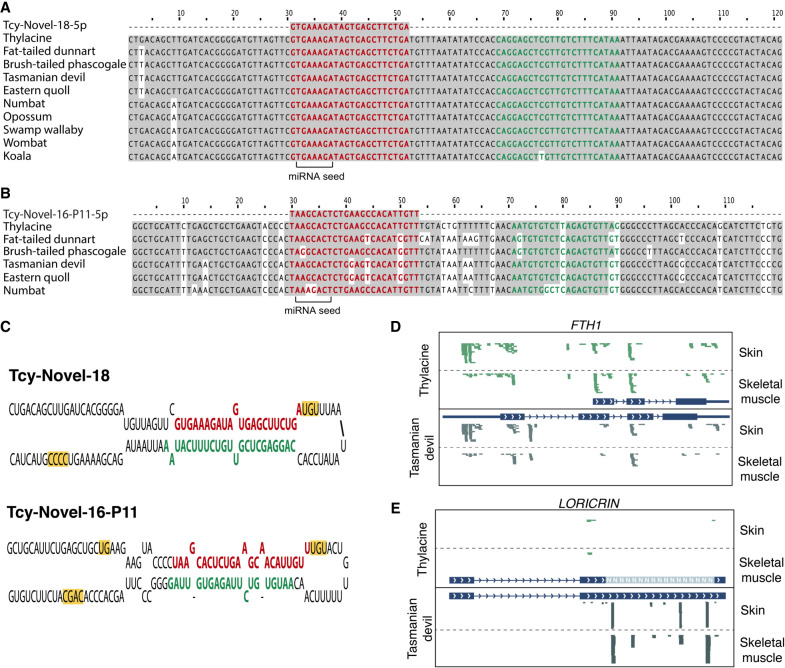
Novel thylacine miRNAs and improved gene annotations guided by historical RNA sequences. Multiple sequence alignment for Tcy-Novel-18 (*A*) and Tcy-Novel-16-P11 (*B*) selected novel miRNA candidates predicted using miRDeep2 software. The 5p arm showing transcriptional evidence in the thylacine RNA sequencing data from skeletal muscle and skin tissue is highlighted in bold red. The 3p arm is highlighted in bold green. Nucleotides that are shared with respect to the thylacine species for each novel miRNA candidate are shown in gray. (*C*) Predicted secondary structure folding of the pri-miRNA precursor sequences (±30 nt from the pre-miRNA) for Tcy-Novel-18 and Tcy-Novel-16-P11 novel miRNA candidates. The 5p and 3p mature arms of the miRNA hairpins are highlighted in bold red and green, respectively. Processing motifs characteristic of true miRNA loci are highlighted in yellow. Examples of missing exonic annotations in the thylacine assembly are shown for transcriptional profiles (in green) obtained for *FTH1* (*D*) and *LORICRIN* (*E*) genes, using the thylacine genome assembly as a reference. RNA sequencing data of skeletal muscle and skin thylacine tissues were aligned to the Tasmanian devil assembly (in gray) for comparison.

One of the selected candidates, named Tcy-Novel-18, was found to be highly conserved in all analyzed marsupial genomes (Fig. 6A) and located in an intron of the E3 ubiquitin ligase ring finger protein 144A (*RNF144A*) gene. This putative novel miRNA did not match any known noncoding RNA loci in the commonly used RNA databases (see Methods). However, mature miRNA transcripts from both stems of the precursor molecule were detected as expressed in Tasmanian devil (Supplemental Fig. 13) and opossum tissues (Supplemental Fig. 14), supporting the reliability of this locus as a true novel miRNA conserved and expressed in related extant marsupials.

The other novel miRNA candidate, named Tcy-Novel-16-P11, partially matched the Sha-Novel-16-P11 miRNA annotated in the Tasmanian devil complement according to MirGeneDB 2.1 (Fromm et al. 2022). The thylacine sequence had three mismatches compared with its Tasmanian devil homologous miRNA, and this pattern was replicated in the Easter quoll (Fig. 6B). Other dasyuromorphids showed one or two mismatches at different positions, indicating variations in the seed region. There was no evidence from public sequence data that this putative miRNA is expressed in any extant species, suggesting that it may be a thylacine-specific miRNA isoform from the NOVEL-16 family. However, because of the conserved “seed” region with Sha-Novel-16-P11 and other equivalent dasyuromorphid homologous loci, it is expected to have a common repertoire of targeted mRNAs and a shared gene regulatory function.

Both novel miRNAs were supported by historical RNA reads with damage patterns consistent with their antiquity (Supplemental File 8), as well as by RNA hairpin-like secondary structures (Fig. 6C) resembling those of bona fide miRNAs in extant species. miRNA-specific processing motifs (Fang and Bartel 2015) were also found in both candidates. Additional information on the two putative novel miRNAs and other candidate loci reported by the miRDeep2 software can be found in Supplemental Table 30.

In summary, we identified two novel miRNA candidates from transcriptional evidence of thylacine skeletal muscle and skin tissues.

### Historical RNA fragments guide improved thylacine gene annotations

We performed comparative mapping of RNA sequences to the reference thylacine and Tasmanian devil assemblies (Supplemental Tables 31–36) to investigate potential imperfections in the thylacine genome annotations. Our analyses revealed two instances of missing thylacine annotations: The ferritin heavy chain 1 gene (*FTH1*) showed differences in the number of exonic regions between the thylacine and Tasmanian devil. Although the current thylacine annotation had three exons, the corresponding homologous locus in the Tasmanian devil assembly had four (Fig. 6D). However, transcriptional evidence from the thylacine historical RNA profiles of skeletal muscle and skin tissues supported the existence of a missing first leading exonic region in the thylacine genome for the *FTH1* gene, as annotated in the Tasmanian devil assembly.

Another example is the *LORICRIN* locus (Fig. 6E). Using the Tasmanian devil genome as a reference, we detected high expression of this gene, with a majority of the mapped reads originating from the skin tissue. However, when mapping to the thylacine assembly, the expression profile of the corresponding gene was barely detected (Supplemental Table 32). Upon closer inspection, we found that most of the coding sequence of the *LORICRIN* gene was missing from the thylacine assembly, represented by unknown “N” nucleotides. This hindered the mapping of reads to this genomic region. The high abundance of RNA reads mapping to the *LORICRIN* gene in the thylacine skin tissue agrees with its relevance as the major protein component of the cornified envelope in terminal epidermal cells of mammals (Yoneda et al. 1992).

In summary, we were able to detect, and partly correct, missing annotations in the thylacine genome assembly using historical transcriptional evidence from RNA sequencing.

## Discussion

In the current study, we present the first successful transcriptomic sequencing evidence from an extinct metazoan species, the Tasmanian tiger (*T. cynocephalus*), also known as the thylacine. The recovery of RNA expression profiles no longer existing in living cells expands the possibility of delving into the biology of extinct animals. Previous studies have reported the sequencing of RNA molecules from an extremely well preserved permafrozen canid that lived in the late Pleistocene, with an estimated age of ∼14,300 yr, as well as from historical wolf skins (Smith et al. 2019; Fromm et al. 2021). The thylacine specimen used in this study has been stored at room temperature in embalmed desiccation for more than a century. Desiccation, mummification, or both have proven to be a favorable environment for the preservation of oligonucleotides (Hekkala et al. 2011; Schuenemann et al. 2017; Rossi et al. 2021; Pedersen et al. 2022; Richards et al. 2022).

Furthermore, the presence of different ranges of tissues still available in the thylacine specimen analyzed entailed a good opportunity to explore one of the main characteristics of RNA profiles that DNA cannot provide: tissue-specific gene expression signatures. We found protein-coding transcripts highly representative of the sampled tissues, including titin and actin for skeletal muscle and keratin for skin. In addition, we found transcriptional profiles that indicate the presence of slow-acting muscle fibers in the skeletal muscle samples and the presence of a putative source of blood infiltration in two of the three skins analyzed, showing the resolution of information that can potentially be extracted from such data.

We verified the authenticity of our data in several ways:

Metatranscriptomic analyses determined that thylacine was the main source of unambiguously assigned reads for both long and short reads, with the main contaminant source likely coming from human manipulation of the specimen. Other contamination sources are doubtful, as they might have arisen from read misassignments owing to reference database biases and sequences mapping to highly conserved loci across species. Despite the high percentage of successful read mapping to the thylacine genome and the damage patterns found in the RNA sequences supporting their antiquity, accurately estimating the true endogenous RNA content is challenging. Unambiguously assigned reads detected by our metatranscriptomic pipeline represent only a small proportion of sequences mapping to species-specific loci, whereas the remaining reads with ambiguous mapping, possibly of endogenous origin, are discarded. Targeted mapping to the thylacine assembly alone provided a high percentage of successful alignment for short trimmed reads in both tissues (∼63%). However, this value was more reduced for longer untrimmed reads, possibly owing to contamination from highly conserved genomic regions across species. The unexpected abundance of long (42-nt) trimmed reads mapped to the human assembly, and their reduced damage profile supports this interpretation. Moreover, the reduced number of long untrimmed reads successfully mapped to the thylacine assembly, along with their damage profiles resembling those of shorter reads, indicates time-dependent nucleotide modifications in our data, further reinforcing the antiquity and the probable thylacine origin of most sequences, whether short or long.

Second, we observed distinctive abundance profiles in the skeletal muscle and skin samples, with rRNAs and tRNAs being the most abundant noncoding transcripts, as expected for eukaryote transcriptomes (Westermann et al. 2012), and protein-coding and miRNA genes showing relevant hallmarks of tissue specificity conserved in extant species. This characteristic pattern would not have been found had the RNA reads come from undisclosed exogenous contamination or from thylacine DNA sequenced together with our RNA extracts. Nevertheless, our estimates of putative unwanted endogenous DNA contamination revealed a reduced percentage <5% of intergenic mapped reads that might belong to sequenced thylacine DNA. We also observed marsupial-specific miRNAs. Although possible, it is unlikely that marsupial-derived contamination from species other than the thylacine would have occurred, given the known history of the specimen and the procedures used during sampling, RNA extraction, and sequencing.

Third, the sequenced RNA molecules showed characteristic patterns of nucleotide substitutions, similar to damage patterns observed in previous ancient RNA studies (Smith et al. 2019; Fromm et al. 2021).

Despite these compelling results, we observed sample-dependent variability in the recovered RNA profiles. One of the three samples per tissue generally provided most of the tissue-specific resolution in coding loci, whereas the other two had more limited profiles. Nevertheless, the overall relative tissue-specific RNA abundance was preserved, as evidenced by the tissue clustering at the miRNA and protein-coding mRNA level using both merged samples and individual samples from skeletal muscle and skin thylacine tissues. The observed differences among samples may have originated from inherent differences in RNA preservation owing to the sampling strategy, as well as technical biases introduced during RNA extraction, library preparation, or sequencing. However, for the majority of the results presented in this study, a merged sample composite for each tissue was considered in order to maximize the resolution and statistical power of our analyses. Because no specific methodologies were applied to remove highly abundant and repetitive elements of the transcriptome, the depth of coverage for RNA transcripts other than highly expressed loci, such as rRNAs or tRNAs, is expected to be low. Future developments could benefit from applying rRNA and/or tRNA fragment depletion protocols to increase the breadth and depth of coverage of low-abundant target transcripts with a similar sequencing effort.

Aside from unraveling patterns of gene activity, another application of RNA sequencing is to detect molecules not present as DNA copies, such as RNA viruses. We found a limited number of RNA molecules that could be assigned to viral genomes. Their presence in such old remains suggests the potential to profile the RNA virome from specimens of extant and extinct species stored in museum dry collections. Evidence of the reconstruction of historical viral genomes has been previously reported (Zhang et al. 2006; Smith et al. 2014), and tracing the origins and evolution of relevant RNA virus families could provide knowledge to prepare mitigation measures for future pandemics (Keusch et al. 2022).

Ancient/historical RNA sequencing opens an unprecedented opportunity to obtain expression evidence of still unknown loci that are virtually impossible to annotate from DNA information alone. We used several complementary approaches to annotate thylacine miRNA loci from our RNA sequence data, increasing the total number of annotated thylacine miRNAs from 62 to 325, thus bringing it on par with other extant mammalian species (Fromm et al. 2022). In addition, we predicted two novel miRNA candidates. Given that one of the novel miRNA candidates contains several nucleotide substitutions relative to homologous loci in other closely related marsupial species (see Results above), its annotation would have been difficult without the expression support of the historical RNA sequences used in this study. The NOVEL-16 family, as annotated in the Tasmanian devil according to MirGeneDB 2.1 (Fromm et al. 2022) and in the annotation produced in this study for the thylacine genome, harbors multiple copies with subtle sequence variations. This denotes an active undergoing evolutionary differentiation for this miRNA family, giving rise to dasyuromorphid-specific isoforms and novel miRNA–mRNA interactions. The other novel miRNA candidate was found to be highly conserved in all analyzed marsupial genomes and to be broadly expressed in Tasmanian devils and opossums. Although it is not clear why this miRNA has eluded previous annotation efforts in extant marsupial species, it clearly shows how analyses of historical RNA sequences from extinct ancestors and sister families can help improve genome annotations and our understanding of the gene regulatory network repertoire evolution in present-day extant species.

The unique characteristics of ancient/historical RNA profiles provide new opportunities to gain deeper knowledge of the genomic architecture and gene expression regulation of extinct species such as the thylacine, a still unexplored area that might benefit recent efforts in the field of de-extinction (Shapiro 2017; Seddon and King 2019). Moreover, exploring other preserved thylacine specimens available across museum collections could greatly improve the transcriptional resolution and tissue diversity reached in the present study. In general, the field of paleotranscriptomics has been neglected and underexplored, with only a few recent examples of reliable data supporting the preservation of ancient transcriptomic profiles from extant species over time (Smith et al. 2014, 2019; Fromm et al. 2021). Despite the limitations in the recovery of RNA extracts from well-preserved remains, the present study adds additional proof of RNA molecules still present and recoverable at amounts sufficient to be representative of true historical transcriptomic profiles in dry museum collections. Because the timescale of RNA preservation seems to range several 1000 yr into the past (Smith et al. 2019; Fromm et al. 2021), we believe that a vast yet unexplored compendium of preserved tissues awaits further analysis in search of long-forgotten transcriptomes. Hence, we advocate for applying transcriptome-based approaches to recover RNA molecules from preserved specimens in dry, and possibly wet, museum collections, fostering a new era of integrative paleo-studies covering genomics, proteomics, and transcriptomics.

## Methods

### Sample collection, RNA extraction, and sequencing

Skeletal muscle and skin tissue samples were collected from an ∼130-yr-old embalmed desiccated thylacine specimen (NRM-MA590213), preserved at room temperature and available at the Stockholm Natural History Museum (Naturhistoriska Riksmuseet [NRM]). This adult specimen was captured on the island of Tasmania and arrived at the NRM collection in 1891 as a donation from the Australian Museum of Sydney. The exact date of death and sex are unknown. Tissue samples were obtained in triplicate and stored at −20°C until use. Skeletal muscle tissue was collected from the inner surface of the left scapula bone, whereas skin tissue was collected from three different sections of the ventrolateral skin flaps and the inguinal region, as shown in Figure 1.

All laboratory work, including tissue subsampling and homogenization, RNA extraction, and library preparation, were performed in dedicated aDNA facilities within the Centre for Palaeogenetics (CPG) at Stockholm University, following strict standard guidelines for working with ancient/historical biomolecules (Knapp et al. 2012).

Approximately 80 mg of tissue per sample was sectioned into small pieces with a scalpel and pulverized in liquid nitrogen using a mortar and pestle. The resulting tissue powder was then added to 900 µL of digestion buffer (Gilbert et al. 2007; Sinding et al. 2015). The resulting lysis mixture was then incubated for 30 min at 37°C. Optionally, for tissue samples that showed almost no digestion after incubation, a further homogenization process was implemented by mechanical lysis with 2 mL PowerBead pro tubes (Thermo Fisher Scientific) loaded with 2.38-mm metallic beads in a TissueLyser LT equipment (Qiagen). Subsequently, the total RNA fraction was isolated using the mirVana miRNA isolation kit (Thermo Fisher Scientific) according to the manufacturer's specifications except for the following: (1) substituting the initial lysis/binding buffer with the previously described incubated homogenized tissue mixture and (2) performing the final elution in 25 µL ultrapure nuclease-free H_2_O and repeating the elution flow through the filter cartridge twice. The total RNA concentration from each eluted extract was determined in triplicate using both Qubit miRNA and Qubit RNA HS (high sensitivity) assay kits in a dedicated Qubit 2.0 fluorometer equipment (Thermo Fisher Scientific). Sequencing libraries were prepared using the NEXTflex small RNA-seq kit v3 protocol (Bioo Scientific) and allowing 23× PCR amplification cycles with no size selection. A positive control sample was included by using a 21-nt miRNA-like sequence not matching any known miRNA in miRBase database (Kozomara et al. 2019) and provided within the NEXTflex small RNA-seq kit. The resulting library concentration was then determined with a Qubit dsDNA broad-range assay kit (Thermo Fisher Scientific), and cDNA fragment size distribution and integrity were assessed with the Agilent high-sensitivity DNA kit assay in a Bioanalyzer 2100 system (Agilent Technologies). Single-end sequencing was performed independently for each tissue on a NextSeq 500 sequencing system (Illumina) using the Illumina NextSeq high-output sequencing reagent kit (75 cycles).

### Sequence preprocessing and quality control

Raw sequenced reads were processed to remove sequencing adapters using cutadapt 3.2 software (Martin 2011), with a minimum read length after adapter trimming of 18 nt per read and a maximum error rate of 10% in adapter sequence detection. Trimmed reads were collapsed and quality control–filtered using the miRTrace *qc* function (Kang et al. 2018) to remove low-quality reads and identical PCR duplicates. UMIs were then removed from the trimmed collapsed sequences using the *trimfq* function (*-b 4 -e 4*) from the Seqtk tool (https://github.com/lh3/seqtk) and retained as sequence ID tags for further deduplication procedures.

### Metatranscriptomics

We performed a taxonomic analysis of the skeletal muscle and skin sequences to estimate the amount of endogenous RNA and identify potential additional RNA contamination. The analysis was conducted before PCR deduplication collapsing and after adapter trimming and UMI removal. Additionally, the same analyses were performed on RNA reads for which sequencing adapters were not identified and were kept untrimmed. The taxonomic classification of the reads was performed using the KrakenUniq software (Breitwieser et al. 2018) with the full NCBI nonredundant reference nucleotide (NT) database. The classification was based on *k*-mer mapping, commonly used with the standard BLASTN algorithm (Altschul et al. 1990). The resulting alignments were filtered based on two specific criteria: (1) “species” level was selected as the taxonomic level, and (2) only “species” with more than 1000 *k*-mers and more than 200 species-specific reads (taxReads) were considered.

Because reads <31 nt could not be classified using the KrakenUniq methodology (which uses a default *k*-mer length of 31), we performed additional alignment using Bowtie 2 v.2.4.2 (Langmead and Salzberg 2012). The reference database used for alignment included the thylacine genome assembly (Feigin et al. 2022), the hg19 human reference genome, and the full NCBI NT database as built in December 2020 (Pochon et al. 2022). The use of the hg19 human assembly, instead of more modern versions like hg38, is not expected to significantly impact the overall results obtained, as human genomic/transcriptomic content is considered to be of contaminant origin and not the main focus of our analyses. We used the global alignment Bowtie 2 mode with the flags *‐‐end-to-end* and *‐‐very-sensitive* and selected ultrashort reads (18–30 nt) that mapped uniquely to one of the references in the merged database (MAPQ ≥ 1). The sequence IDs were matched with their corresponding taxIDs using the seqid2taxid.map mapping file constructed using Kraken2 software (Wood et al. 2019) from the NCBI NT taxonomy information. The number of reads assigned to each taxID was quantified, and organisms detected in skeletal muscle and skin tissue samples were ranked based on abundance. The filtered taxonomic assignment proportions were visualized using KronaTools (Ondov et al. 2011). Data visualization and subsequent analyses were conducted using ggplot2 graphics (Wickham 2016) within R software (R Core Team 2022).

### Mapping and quantification

Transcriptome sequence models and exon–exon maps for annotated genes in the thylacine chromosome-based nuclear genome assembly (Feigin et al. 2022) were generated using the gffread v0.12.6 tool (*-Z -W ‐‐force-exons ‐‐gene2exon ‐‐t-adopt ‐‐tlf*). Exonic, intronic, and intron–intron maps were deduced from genome-wide and transcriptome-wide annotations. Only the longest transcript isoforms per gene were considered for further analyses. PCR deduplicated and adapter-trimmed/untrimmed sequences without UMIs from skeletal muscle and skin tissues were mapped against a composite of the thylacine nuclear and mitochondrial genomes (NC_011944.1) (Miller et al. 2009), as well as to the nuclear transcriptome assembly using the Bowtie aligner tool v1.3.0 (Langmead et al. 2009). The alignment allowed up to one mismatch within a seed equal to the minimum read length (18 nt) and reported a maximum of one valid alignment with high sensitivity (*-n 1 -l 18 -k 1 -y ‐‐best*). To account for sequence replication, repetitive and degenerated UMIs were removed using the *dedup* function from UMI-Tools v1.1.2 software (Smith et al. 2017) with the directional methodology for UMI clustering. UMI identifiers were obtained from trimmed reads (8-mer UMIs) and untrimmed reads (4-mer UMIs) after adapter removal. Additionally, the last 4 nt of untrimmed reads was removed to prevent alignment biases.

RNA transcript abundance on a per gene basis was quantified based on UMI-deduplicated alignments, and the breadth of coverage for each gene was determined using the coverage function of BEDTools v2.30.0 (Quinlan and Hall 2010). Genes with a breadth of coverage of at least 10% of the entire coding sequence were considered to have reliable expression evidence. A similar procedure was applied to map RNA reads from thylacine skeletal muscle and skin tissues to the Tasmanian devil nuclear genomic DNA + mitochondrial DNA (NC_018788.1) (Miller et al. 2011) and the transcriptome assembly obtained from NCBI annotation (mSarHar1.11).

An investigation was conducted to explore unexpected fish-related contamination identified by KrakenUniq and Bowtie 2–based metatranscriptomic analyses. Trimmed deduplicated RNA reads from thylacine skeletal muscle and skin tissues were mapped to the zebrafish reference genome assembly (GRCz11) using the Bowtie aligner v1.3.0 (*-v 0 -k 1 ‐‐best -y*) (Langmead et al. 2009). Additionally, trimmed RNA reads assigned to zebrafish, human, or mouse genomes by KrakenUniq were realigned (*-n 1 -l 18 -k 1 -y ‐‐best*) to the thylacine (Feigin et al. 2022), Tasmanian devil (mSarHar1.11), and opossum (MonDom5) assemblies to reevaluate their origin.

Additional analyses describing the identification of expression hotspots genome-wide, rRNA annotation, DNA contamination, and exonic/intronic RNA enrichment are detailed in the Supplemental Methods.

### RNA damage

Because no UDG treatment (Briggs et al. 2010) was implemented during RNA extraction and library preparation to correct for cytosine deamination events, damage patterns in RNA sequences mapped to the thylacine assembly after PCR and UMI deduplication were assessed using the *platypus* function from PMDtools software (Skoglund et al. 2014). Damage analyses were performed for trimmed RNA reads of different length ranges and for untrimmed reads. Additionally, a comparative analysis of sequence damage patterns was conducted for the observed excess of long trimmed reads of 42 nt in length. Deamination profiles from reads of 42 nt mapped to both the thylacine and human assemblies (hg19) were compared with those from reads of 42 nt that mapped only to the thylacine assembly. Reads mapping to the human genome were considered as potential modern contamination.

### miRNA annotation

We used the thylacine genome assembly (Feigin et al. 2022) together with MirGeneDB 2.1 reference miRNA annotation (Fromm et al. 2022) as inputs of MirMachine software (Umu et al. 2023) to predict miRNA loci based on homology searches across the thylacine genome. In addition, annotated miRNA hairpins from opossum and Tasmanian devil according to MirGeneDB 2.1 (Fromm et al. 2022) were mapped to the thylacine assembly to identify homologous marsupial-derived miRNA loci. PCR- and UMI-deduplicated RNA reads from thylacine skeletal muscle and skin tissues were then used with the MirMiner algorithm (Wheeler et al. 2009) for the annotation of additional novel miRNA genes. The criteria for annotating novel miRNA genes included (1) the expression of at least two 18- to 25-nt-long reads from each arm of the putative miRNA hairpin precursor, (2) consistent 5′-end homogeneity of supporting RNA reads, (3) at least 16-nt complementarity between the predicted arms of the precursor miRNA, and (4) a loop sequence length between 8 and 40 nt. A summary of the miRNA annotation pipeline is provided in Supplemental Figure 15. The predicted set of pre-miRNA hairpins was elongated by 30 nt on both sides to build primary miRNA hairpin annotations for mapping and quantification purposes.

### Mapping to miRNA loci and quantification

Trimmed RNA reads in the range of 18–25 nt, as well as untrimmed reads, were mapped to the set of predicted pri-miRNA hairpin precursors in the thylacine genome using the Bowtie aligner tool v1.3.0 (*-n 1 -l 18 -k 1 -y ‐‐best*) (Langmead et al. 2009). A similar procedure was performed using Tasmanian devil pri-miRNA hairpins annotated according to MirGeneDB 2.1 (Fromm et al. 2022). Reads mapping outside mature miRNAs within the stem of pri-miRNA hairpins and those with offset nucleotides covering >25% of their sequence length with respect to the mature miRNAs were considered unreliable mapping events and discarded. UMI deduplication was performed, and the mature 5p and 3p arms were quantified separately. The ratio between 5p and 3p abundance was then computed for each miRNA precursor with successfully mapped reads supporting its expression.

### Tissue clustering

We aimed to determine whether the expression profiles identified in thylacine skeletal muscle and skin tissue resembled any expected miRNA and/or protein-coding mRNA expression profiles in modern tissues from extant related species. For miRNAs, samples from the miRNA abundance tissue atlas of Tasmanian devil (*S. harrisii*), opossum (*M. domestica*), and domestic dog (*C. familiaris*) (Koenig et al. 2016; Penso-Dolfin et al. 2016) available in MirGeneDB 2.1 (Fromm et al. 2022) were selected. Thylacine miRNA abundance profiles for all annotated miRNAs (N = 325) were transformed to counts per million (CPM) estimates based on the total number of genome-wide mapped reads after UMI deduplication. Only miRNAs with shared homologous loci among dogs, Tasmanian devils, opossums, and thylacines were retained (N = 119).

For protein-coding mRNAs, we used a gene expression atlas in sheep (*O. aries*) (Jiang et al. 2014) available at the EMBL-EBI Expression Atlas database (Papatheodorou et al. 2020), along with Tasmanian devil RNA-seq data from different tissues (Stammnitz et al. 2023). The selected protein-coding genes were those reliably captured in our thylacine historical RNA sequencing data in both skeletal muscle and skin tissues (breadth of coverage >10%), with a shared homologous locus among sheep, Tasmanian devils, and thylacines (N = 261). Expression profiles for protein-coding mRNAs were transformed to the log_2_ scale. To perform a dimensionality reduction of protein-coding and miRNA profiles independently, the uniform manifold approximation and projection (UMAP) algorithm (McInnes et al. 2018) was implemented using the UMAP R package (https://github.com/tkonopka/umap) with the following specifications: *n_neighbors = 5*, *metric =*“*pearson*,” *spread = 10*, *random_state = 30*. Thylacine tissues were excluded from the initial embedding estimation. Finally, the thylacine expression profiles of protein-coding mRNAs and miRNAs were projected onto their corresponding previously learned UMAP embeddings.

### Novel miRNA prediction

We further implemented the miRDeep2 software (Friedländer et al. 2012) in an attempt to capture additional putative miRNA loci in the thylacine genome. The *mapper.pl* function (*-d -c -m*) was used to jointly align trimmed PCR- and UMI-deduplicated thylacine skeletal muscle and skin RNA sequences against the thylacine assembly (Feigin et al. 2022). The *miRDeep2.pl* function was then run, including thylacine pre-miRNA and mature miRNA sequences, as well as Tasmanian devil mature miRNA sequences for cross-species identification. The mature miRNA sequences of the selected novel miRNA candidates were mapped to miRBase v22.1 (Kozomara et al. 2019), MirGeneDB 2.1 (Fromm et al. 2022), and Rfam 14 (Kalvari et al. 2021) to identify any overlaps with already annotated loci. Secondary structure folding was predicted using the mFold software (Zuker 2003), and the presence of conserved homologous loci in additional marsupial genome assemblies was assessed, including the Tasmanian devil (*S. harrisii*; mSarHar1.11), numbat (*Myrmecobius fasciatus*), Eastern quoll (*Dasyurus viverrinus*; DasViv_v1.0), fat-tailed dunnart (*Sminthopsis crassicaudata*), brush-tailed phascogale (*Phascogale tapoatafa*), wombat (*Vombatus ursinus*), opossum (*M. domestica*, MonDom5), swamp wallaby (*Wallabia bicolor*), and koala (*Phascolarctos cinereus*; phaCin_unsw_v4.1). The brush-tailed phascogale, numbat, and swamp wallaby genome assemblies were obtained from the publicly available DNA Zoo Consortium (https://www.dnazoo.org/) database generated through 3D de novo assembly (Dudchenko et al. 2017). The fat-tailed dunnart genome assembly was generated using an unpublished draft from the University of Melbourne and was available through the DNA Zoo Consortium database. The remaining assemblies correspond to the last version available in the NCBI database. A schematic representation of the evolutionary relationship among marsupial species is shown in Supplemental Figure 16. Multiple sequence alignment was performed using the Clustal Omega software (Sievers et al. 2011). Small RNA-seq data from Tasmanian devil and opossum species were mapped to their corresponding genome assemblies (mSarHar1.11 and MonDom5, respectively) to detect transcriptional evidence of the selected novel miRNA candidate homologous loci. Alignment was performed using Bowtie aligner v1.3.0 (Langmead et al. 2009), allowing a maximum of two mismatches (*-v 2 -k 1 ‐‐best -y*). Small RNA-seq data sets are available at the NCBI Gene Expression Omnibus (GEO; https://www.ncbi.nlm.nih.gov/geo/) under accession numbers GSE18352 (Murchison et al. 2010) and GSE40499 (Meunier et al. 2013).

## Data access

The sequence data generated in this study have been submitted to the NCBI BioProject database (https://www.ncbi.nlm.nih.gov/bioproject/) under accession number PRJNA900297.

## Supplementary Material

Supplement 1

Supplement 2

Supplement 3

Supplement 4

Supplement 5

Supplement 6

Supplement 7

Supplement 8

Supplement 9
