# Supplementary material for: Historical RNA expression profiles from the extinct Tasmanian tiger
Source: Genome Res. 2023 Aug;33(8):1299–316. doi: 10.1101/gr.277663.123 (PMC10552650; doi:10.1101/gr.277663.123)
Supplement: Supplement 3 [file Supplemental_File_3.html]

Javascript must be enabled to view this page.

members
magnitude
magnitudeUnassigned
count
unassigned
taxon
rank

Thylacine\_Muscle\_merged\_over18nt\_noUMIs\_sequences.krakenuniq\_kmers1000

1390405

2759
1388424
superkingdom

kingdom
33090
3534

3534
35493
phylum

131221
3534
subphylum

clade
3193
3534

clade
58023
3534

clade
3534
78536

clade
58024
3534

3398
3534
class

3534
1437183
clade

clade
71240
3534

clade
91827
3534

3534
1437201
clade

71275
3534
clade

91836
3534
clade

order
3699
3534

family
3700
3534

tribe
3534
981071

3534
3705
genus

species
node20.members.0.js
3534
3711

1343172
33154
clade

kingdom
1294887
33208

clade
1294887
6072

clade
1294887
33213

clade
1294887
33511

1294887
7711
phylum

1294887
89593
subphylum

clade
1294887
7742

clade
1294887
7776

117570
1294887
clade

clade
1294887
117571

1123781
8287
superclass

clade
1338369
1123781

32523
1123781
clade

class
8292
9574

9574
8445
order

30380
5058
family

5058
194407
genus

node39.members.0.js
5058
194408
species

4516
1277737
family

264009
4516
genus

species
node42.members.0.js
4516
1415580

clade
1114207
32524

1114207
40674
class

clade
32525
1114207

9347
169294
clade

clade
1437010
169294

169072
314146
superorder

clade
314147
70100

9989
70100
order

suborder
1963758
70100

clade
70100
337687

family
10066
70100

subfamily
39107
70100

70100
10088
genus

subgenus
70100
862507

70100
10090
node57.members.0.js
species

order
98972
9443

98972
376913
suborder

314293
98972
infraorder

9526
98972
parvorder

superfamily
98972
314295

9604
98972
family

subfamily
207598
98972

9605
98972
genus

9606
98972
node66.members.0.js
species

superorder
222
314145

order
222
33554

379584
222
suborder

family
222
9608

9611
222
genus

node72.members.0.js
222
9612
species

944913
9263
clade

order
38605
209363

family
209363
9265

209363
126287
subfamily

genus
209363
13615

species
node78.members.0.js
13616
209363

38608
735550
order

family
643118
9273

9274
643118
genus

species
9275
643118
node82.members.0.js

family
92432
9277

genus
9304
92432

species
9305
92432
node85.members.0.js

superclass
7898
171106

class
186623
171106

41665
171106
subclass

infraclass
171106
32443

clade
1489341
171106

no rank
186625
171106

107340
186634
cohort

subcohort
282425
1469

order
32446
1469

1489459
1469
suborder

family
1469
299319

genus
299320
1469

species
1469
299321
node98.members.0.js

32519
105871
subcohort

clade
186626
105871

105871
186627
superorder

105871
7952
order

suborder
30727
105871

family
105871
2743709

2743711
105871
subfamily

105871
7954
genus

node107.members.0.js
7955
105871
species

1489388
63766
cohort

clade
123365
55000

clade
123366
55000

123367
55000
clade

clade
123368
55000

4660
1489838
clade

1489841
4660
clade

1489843
4660
clade

4660
8043
order

4660
1489845
suborder

family
4660
8045

4660
8048
genus

node120.members.0.js
8049
4660
species

clade
123369
50340

1489872
50340
clade

1489908
45612
clade

1489920
9978
clade

order
1489921
9978

123349
4649
suborder

family
63826
4649

subfamily
4649
557415

genus
4649
210581

species
node130.members.0.js
4649
441366

suborder
5329
56717

56718
5329
family

subfamily
703913
5329

5329
94311
genus

node135.members.0.js
181472
5329
species

35634
1489913
superorder

order
76071
35634

28781
35634
suborder

35634
47757
family

subfamily
8088
35634

genus
35634
8089

species
node142.members.0.js
35634
8090

clade
1489874
4728

4728
8064
order

4728
8065
family

subfamily
4728
390319

genus
289381
4728

4728
390379
node148.members.0.js
species

clade
8766
41705

order
8006
8766

8015
8766
family

378
504567
subfamily

27772
378
genus

no rank
378
2649731

node155.members.0.js
861768
378
species

subfamily
504568
8388

genus
8388
8028

node158.members.0.js
8388
8032
species

kingdom
48285
4751

451864
2960
subkingdom

phylum
4890
2960

clade
822
716545

822
147538
subphylum

822
716546
clade

822
147545
class

subclass
822
451871

5042
822
order

822
1131492
family

genus
822
5073

254878
822
no rank

1108849
822
node171.members.0.js
species

2138
136265
no rank

node173.members.0.js
175243
2138
species

no rank
57731
45325

species
node175.members.0.js
45325
175245

no rank
61964
41718

100272
41718
node177.members.0.js
species

superkingdom
1981
2

no rank
48479
1981

node180.members.0.js
1981
77133
species
