# Supplementary material for: Historical RNA expression profiles from the extinct Tasmanian tiger
Source: Genome Res. 2023 Aug;33(8):1299–316. doi: 10.1101/gr.277663.123 (PMC10552650; doi:10.1101/gr.277663.123)
Supplement: Supplement 4 [file Supplemental_File_4.html]

Javascript must be enabled to view this page.

members
magnitude
magnitudeUnassigned
count
unassigned
taxon
rank

Thylacine\_Muscle\_merged\_Untrimmed\_noUMIs\_sequences.krakenuniq\_kmers1000

139117

2759
139117
superkingdom

kingdom
840
33090

840
phylum
35493

840
subphylum
131221

3193
840
clade

clade
840
58023

840
clade
78536

840
clade
58024

class
840
3398

840
clade
1437183

71240
840
clade

clade
840
91827

clade
840
1437201

840
clade
71275

91836
clade
840

3699
840
order

3700
family
840

840
tribe
981071

3705
genus
840

species
3711
node20.members.0.js
840

133979
clade
33154

17966
kingdom
4751

451864
1746
subkingdom

phylum
1746
4890

1473
clade
716545

1473
subphylum
147538

716546
clade
1473

1473
class
147545

1473
subclass
451871

5042
1473
order

1131492
1473
family

939
genus
5052

939
subgenus
2720871

node34.members.0.js
species
5061
939

534
genus
5073

254878
534
no rank

1108849
species
node37.members.0.js
534

273
no rank
136265

273
node39.members.0.js
species
175243

no rank
16220
57731

16220
node41.members.0.js
species
175245

33208
116013
kingdom

6072
116013
clade

116013
clade
33213

33511
116013
clade

7711
116013
phylum

89593
116013
subphylum

7742
116013
clade

clade
116013
7776

clade
116013
117570

clade
116013
117571

8287
80202
superclass

clade
80202
1338369

80202
clade
32523

8292
class
828

8445
828
order

828
family
30380

194407
genus
828

828
node59.members.0.js
194408
species

79374
clade
32524

40674
class
79374

32525
79374
clade

9347
clade
15149

1437010
clade
15149

314146
15149
superorder

314147
9809
clade

9989
9809
order

suborder
9809
1963758

337687
9809
clade

family
9809
10066

39107
9809
subfamily

10088
9809
genus

862507
9809
subgenus

9809
node74.members.0.js
species
10090

5340
order
9443

suborder
5340
376913

314293
infraorder
5340

parvorder
5340
9526

314295
superfamily
5340

9604
5340
family

207598
subfamily
5340

9605
5340
genus

node83.members.0.js
species
9606
5340

9263
64225
clade

38608
64225
order

9273
57390
family

57390
genus
9274

57390
species
9275
node88.members.0.js

9277
6835
family

9304
6835
genus

6835
node91.members.0.js
species
9305

35811
superclass
7898

35811
class
186623

41665
35811
subclass

35811
infraclass
32443

35811
clade
1489341

186625
35811
no rank

186634
11961
cohort

subcohort
11961
32519

11961
clade
186626

11961
superorder
186627

11961
order
7952

11961
suborder
30727

11961
family
2743709

2743711
11961
subfamily

genus
11961
7954

node107.members.0.js
7955
species
11961

23850
cohort
1489388

clade
873
41705

8006
873
order

873
family
8015

504568
subfamily
873

8028
873
genus

node114.members.0.js
species
8032
873

22977
clade
123365

123366
22977
clade

123367
22977
clade

123368
clade
22977

123369
clade
22344

1489872
22344
clade

1489908
21687
clade

1489913
19076
superorder

76071
19076
order

28781
suborder
19076

19076
family
47757

8088
19076
subfamily

19076
genus
8089

8090
species
node128.members.0.js
19076

1489920
clade
2611

1489921
2611
order

suborder
1467
56717

family
1467
56718

1467
subfamily
703913

genus
1467
94311

species
181472
node135.members.0.js
1467

suborder
1144
123349

63826
family
1144

subfamily
1144
557415

210581
genus
1144

1144
species
441366
node140.members.0.js

1489874
657
clade

657
order
8064

657
family
8065

657
subfamily
390319

genus
657
289381

657
390379
species
node146.members.0.js

1489838
clade
633

1489841
633
clade

1489843
633
clade

633
order
8043

1489845
suborder
633

8045
633
family

8048
genus
633

node154.members.0.js
species
8049
633

61964
no rank
4298

species
100272
node156.members.0.js
4298
