# Supplementary material for: Historical RNA expression profiles from the extinct Tasmanian tiger
Source: Genome Res. 2023 Aug;33(8):1299–316. doi: 10.1101/gr.277663.123 (PMC10552650; doi:10.1101/gr.277663.123)
Supplement: Supplement 5 [file Supplemental_File_5.html]

Javascript must be enabled to view this page.

members
magnitude
magnitudeUnassigned
count
unassigned
taxon
rank

Thylacine\_Skin\_merged\_over18nt\_noUMIs\_sequences.krakenuniq\_kmers1000

3222610

superkingdom
3218565
2759

clade
3157262
33154

kingdom
3061610
33208

6072
clade
3061610

33213
clade
3061610

3061610
clade
33511

7711
3061610
phylum

89593
subphylum
3061610

clade
3061610
7742

7776
clade
3061610

117570
3061610
clade

clade
3061610
117571

8287
superclass
2560294

1338369
clade
2560294

32523
clade
2560294

32524
2522001
clade

8457
12951
clade

12951
clade
32561

clade
12951
1329799

12951
clade
8492

clade
12951
436486

12951
clade
436489

clade
12951
436491

12951
clade
436492

12951
class
8782

8825
12951
infraclass

9126
12951
order

114313
12951
family

88178
genus
12951

node30.members.0.js
species
12951
649802

40674
class
2509050

2509050
clade
32525

2191668
clade
9263

38608
order
1922819

9277
87958
family

87958
genus
9304

9305
87958
species
node37.members.0.js

9273
1834861
family

9274
1834861
genus

9275
node40.members.0.js
species
1834861

38605
order
268849

268849
family
9265

126287
268849
subfamily

268849
genus
13615

species
node45.members.0.js
268849
13616

clade
317382
9347

1437010
317382
clade

4341
superorder
314145

9397
order
736

30560
suborder
736

9431
736
family

736
genus
27671

736
species
node53.members.0.js
59474

33554
order
3605

379584
3605
suborder

9608
family
3605

9611
3605
genus

9612
species
node58.members.0.js
3605

314146
superorder
313041

clade
161323
314147

161323
order
9989

1963758
suborder
161323

clade
161323
337687

family
161323
10066

39107
161323
subfamily

10088
genus
161323

161323
subgenus
862507

10090
node68.members.0.js
species
161323

151718
order
9443

376913
suborder
151718

151718
infraorder
314293

9526
151718
parvorder

314295
151718
superfamily

9604
151718
family

151718
subfamily
207598

9605
genus
151718

9606
151718
species
node77.members.0.js

class
38293
8292

8445
order
38293

30380
14859
family

194407
genus
14859

194408
14859
node82.members.0.js
species

1277737
23434
family

264009
23434
genus

1415580
node85.members.0.js
species
23434

superclass
501316
7898

501316
class
186623

41665
subclass
501316

32443
501316
infraclass

501316
clade
1489341

no rank
501316
186625

198441
cohort
1489388

41705
clade
29159

order
29159
8006

family
29159
8015

29159
subfamily
504568

29159
genus
8028

species
node98.members.0.js
29159
8032

clade
169282
123365

123366
169282
clade

123367
169282
clade

123368
169282
clade

1489838
19547
clade

clade
19547
1489841

19547
clade
1489843

8043
19547
order

1489845
19547
suborder

family
19547
8045

8048
19547
genus

node110.members.0.js
species
19547
8049

123369
149735
clade

149735
clade
1489872

1489922
clade
217

8111
217
order

suborder
217
1489943

family
217
30871

274794
217
subfamily

1505891
tribe
217

94231
genus
217

217
species
node120.members.0.js
293821

1489874
6595
clade

8064
6595
order

6595
family
8065

subfamily
6595
390319

genus
6595
289381

6595
species
node126.members.0.js
390379

1489908
clade
137264

33101
clade
1489920

1489921
33101
order

123349
26343
suborder

63826
family
26343

557415
subfamily
26343

210581
26343
genus

species
node134.members.0.js
26343
441366

56717
suborder
6758

56718
6758
family

703913
subfamily
6758

6758
genus
94311

181472
node139.members.0.js
species
6758

104163
superorder
1489913

104163
order
76071

suborder
104163
28781

47757
family
104163

8088
subfamily
104163

8089
104163
genus

104163
node146.members.0.js
species
8090

5659
clade
1489892

1489900
5659
order

suborder
5659
50370

270602
family
5659

158449
subfamily
5659

genus
5659
158455

node153.members.0.js
species
5659
158456

cohort
302875
186634

subcohort
295431
32519

clade
295431
186626

superorder
295431
186627

7952
order
295431

30727
295431
suborder

295431
family
2743709

295431
subfamily
2743711

295431
genus
7954

node163.members.0.js
species
295431
7955

subcohort
7444
282425

order
7444
32446

1489459
7444
suborder

299319
family
7444

genus
7444
299320

node169.members.0.js
species
7444
299321

4751
kingdom
95652

451864
subkingdom
5909

4890
5909
phylum

136265
4130
no rank

175243
node174.members.0.js
species
4130

716545
clade
1779

1779
subphylum
147538

716546
clade
1779

1779
class
147545

subclass
1779
451871

1779
order
5042

family
1779
1131492

genus
1301
5073

no rank
1301
254878

1301
species
node184.members.0.js
1108849

478
genus
5052

2720871
subgenus
478

node187.members.0.js
species
478
5061

no rank
89743
57731

89743
node189.members.0.js
species
175245

no rank
44212
61964

node191.members.0.js
species
44212
100272

17091
kingdom
33090

35493
17091
phylum

131221
subphylum
17091

3193
17091
clade

58023
17091
clade

78536
clade
17091

58024
clade
17091

3398
class
17091

clade
17091
1437183

71240
clade
17091

91827
17091
clade

17091
clade
1437201

71275
clade
17091

91836
clade
17091

order
17091
3699

3700
family
17091

981071
tribe
17091

3705
genus
17091

3711
17091
node210.members.0.js
species

4045
superkingdom
2

48479
no rank
4045

species
node213.members.0.js
4045
77133
