# Supplementary material for: Historical RNA expression profiles from the extinct Tasmanian tiger
Source: Genome Res. 2023 Aug;33(8):1299–316. doi: 10.1101/gr.277663.123 (PMC10552650; doi:10.1101/gr.277663.123)
Supplement: Supplement 6 [file Supplemental_File_6.html]

Javascript must be enabled to view this page.

members
magnitude
magnitudeUnassigned
count
unassigned
taxon
rank

Thylacine\_Skin\_merged\_Untrimmed\_noUMIs\_sequences.krakenuniq\_kmers1000

440052

2
superkingdom
1239

1239
no rank
48479

node3.members.0.js
species
77133
1239

438813
2759
superkingdom

23599
no rank
61964

100272
23599
node6.members.0.js
species

clade
33154
413188

4751
kingdom
23951

subkingdom
451864
3083

3083
4890
phylum

no rank
136265
2630

2630
175243
species
node12.members.0.js

716545
clade
453

453
subphylum
147538

clade
716546
453

147545
class
453

453
subclass
451871

5042
order
453

453
family
1131492

5073
genus
453

254878
no rank
453

1108849
453
species
node22.members.0.js

no rank
57731
20868

node24.members.0.js
species
175245
20868

kingdom
33208
389237

clade
6072
389237

389237
clade
33213

33511
clade
389237

389237
phylum
7711

389237
89593
subphylum

389237
7742
clade

389237
7776
clade

clade
117570
389237

389237
117571
clade

superclass
7898
114964

186623
class
114964

114964
subclass
41665

32443
infraclass
114964

1489341
clade
114964

114964
no rank
186625

1489388
cohort
65701

60873
clade
123365

60873
123366
clade

123367
clade
60873

60873
clade
123368

clade
123369
57459

57459
1489872
clade

50612
1489908
clade

1489913
superorder
43037

76071
order
43037

43037
28781
suborder

43037
47757
family

subfamily
8088
43037

genus
8089
43037

species
node55.members.0.js
8090
43037

1489920
clade
7575

1489921
order
7575

123349
suborder
4531

63826
family
4531

557415
subfamily
4531

210581
genus
4531

4531
441366
species
node62.members.0.js

3044
suborder
56717

3044
56718
family

subfamily
703913
3044

3044
genus
94311

species
node67.members.0.js
181472
3044

347
1489922
clade

order
8111
347

347
1489943
suborder

30871
family
347

274794
subfamily
347

1505891
tribe
347

genus
94231
347

293821
347
species
node75.members.0.js

3225
1489874
clade

order
8064
3225

3225
8065
family

subfamily
390319
3225

3225
genus
289381

390379
3225
node81.members.0.js
species

1489892
clade
3275

order
1489900
3275

suborder
50370
3275

family
270602
3275

158449
subfamily
3275

3275
158455
genus

158456
3275
species
node88.members.0.js

3414
clade
1489838

3414
1489841
clade

clade
1489843
3414

3414
order
8043

suborder
1489845
3414

3414
family
8045

8048
genus
3414

3414
8049
node96.members.0.js
species

4828
41705
clade

4828
8006
order

family
8015
4828

subfamily
504568
4828

4828
8028
genus

4828
8032
species
node102.members.0.js

49263
cohort
186634

subcohort
282425
1388

order
32446
1388

suborder
1489459
1388

1388
299319
family

1388
299320
genus

299321
1388
species
node109.members.0.js

47875
32519
subcohort

47875
186626
clade

47875
186627
superorder

order
7952
47875

47875
suborder
30727

39938
family
2743709

2743711
subfamily
39938

genus
7954
39938

39938
7955
species
node118.members.0.js

7937
7953
family

7937
subfamily
2743694

7956
genus
7937

7957
7937
node122.members.0.js
species

8287
superclass
274273

1338369
clade
274273

274273
32523
clade

32524
clade
271294

class
40674
271294

32525
clade
271294

9347
clade
49801

49801
1437010
clade

234
314145
superorder

234
order
33554

suborder
379584
234

9608
family
234

234
9611
genus

species
node136.members.0.js
234
9612

314146
superorder
49567

order
9443
28410

suborder
376913
28410

28410
infraorder
314293

28410
9526
parvorder

superfamily
314295
28410

9604
family
28410

28410
207598
subfamily

28410
genus
9605

9606
28410
species
node146.members.0.js

21157
clade
314147

order
9989
21157

21157
suborder
1963758

21157
clade
337687

21157
family
10066

21157
subfamily
39107

10088
genus
21157

21157
subgenus
862507

10090
21157
node155.members.0.js
species

9263
clade
221493

38608
order
221493

5814
family
9277

5814
genus
9304

9305
5814
species
node160.members.0.js

215679
9273
family

9274
genus
215679

9275
215679
species
node163.members.0.js

class
8292
2979

order
8445
2979

1871
30380
family

1871
194407
genus

node168.members.0.js
species
1871
194408

family
1277737
1108

264009
genus
1108

1108
1415580
species
node171.members.0.js

2026
33090
kingdom

phylum
35493
2026

131221
subphylum
2026

2026
clade
3193

58023
clade
2026

clade
78536
2026

clade
58024
2026

2026
3398
class

1437183
clade
2026

2026
71240
clade

91827
clade
2026

1437201
clade
2026

2026
clade
71275

91836
clade
2026

order
3699
2026

3700
family
2026

2026
981071
tribe

genus
3705
2026

3711
2026
node190.members.0.js
species
