# Supplementary material for: Historical RNA expression profiles from the extinct Tasmanian tiger
Source: Genome Res. 2023 Aug;33(8):1299–316. doi: 10.1101/gr.277663.123 (PMC10552650; doi:10.1101/gr.277663.123)
Supplement: Supplement 8 [file Supplemental_Files.pdf]

Historical RNA expression profiles from the extinct Tasmanian tiger

Emilio Mármol-Sánchez[1,2*], Bastian Fromm[1,3], Nikolay Oskolkov[4], Zoé Pochon[2,5], Panagiotis Kalogeropoulos[1], Eli Eriksson[1], Inna Biryukova[1], Vaishnovi Sekar[1], Erik Ersmark[2,6], Björn Andersson[7], Love Dalén[2,6,8#*] and Marc R. Friedländer[1#*]

[1]Department of Molecular Biosciences, The Wenner-Gren Institute, Science for Life Laboratory, Stockholm University, Stockholm, Sweden. [2]Centre for Palaeogenetics, Stockholm, Sweden. [3]The Arctic University Museum of Norway, UiT, The Arctic University of Norway, Tromsø, Norway. [4]Department of Biology, National Bioinformatics Infrastructure Sweden, Science for Life Laboratory, Lund University, Lund, Sweden. [5]Department of Archaeology and Classical Studies, Stockholm University, Stockholm, Sweden. [6]Department of Bioinformatics and Genetics, Swedish Museum of Natural History, Stockholm, Sweden. [7]Department of Cell and Molecular Biology, Karolinska Institute, Stockholm, Sweden. [8]Department of Zoology, Stockholm University, Stockholm, Sweden. [#]LD and MRF contributed equally. [*]Corresponding authors: MRF, LD and EMS.

Corresponding authors:

Marc R. Friedländer: marc.friedlander@scilifelab.se

Love Dalén: love.dalen@zoologi.su.se

Emilio Mármol-Sánchez: emilio.marmol.sanchez@gmail.com

Supplemental Files

Supplemental File 1: miRTrace quality control report using RNA trimmed sequences from skeletal muscle and skin tissues.

Supplemental File 2: miRTrace quality control report using untrimmed RNA sequences from skeletal muscle and skin tissues.

Supplemental File 3: Metatranscriptomic classification of trimmed RNA reads >30 nt from skeletal muscle tissue according to the KrakenUniq pipeline (>1000 *k*-mers; >200 species-specific reads).

Supplemental File 4: Metatranscriptomic classification of untrimmed RNA reads >30 nt from skeletal muscle tissue according to the KrakenUniq pipeline (>1000 *k*-mers; >200 species-specific reads).

Supplemental File 5: Metatranscriptomic classification of trimmed RNA reads >30 nt from skin tissue according to the KrakenUniq pipeline (>1000 *k*-mers; >200 species-specific reads).

Supplemental File 6: Metatranscriptomic classification of untrimmed RNA reads >30 nt from skin tissue according to the KrakenUniq pipeline (>1000 *k*-mers; >200 species-specific reads).

Supplemental File 7: FASTA file of pre-miRNA hairpin sequences from the entire list of annotated microRNAs in the thylacine genome (N = 325).

Supplemental File 8: Pre-miRNA hairpin structure and supporting RNA reads of two selected putative novel thylacine-specific microRNA candidates according to the miRDeep2 algorithm. Msc: skeletal muscle. Skn: skin. The miRNA identified as scaffold_2_6678 corresponds to Tcy-Novel-18, while scaffold_7_26772 corresponds to Tcy-Novel-16-P11.

*__Supplemental Files 1-6__ can be found in html format in the Extended Data online.

Supplemental File 7

>Tcy-Let-7-P1c scaffold_3:238189863-238189929(+)
UGAGGUAGUAGGUUGUAUGGUUUAGAAUUAUAUCCUAGGAGUUAACUGUACAACCUUCUAGCUUUC
>Tcy-Let-7-P1d scaffold_3:374122242-374122308(-)
UGAGGUAGUAGGUUGUAUAGUUUAGAAUUACAUCAAGGGAGAUAACUGUACAGCCUCCUAGCUUUC
>Tcy-Let-7-P2a1 scaffold_1:65441657-65441729(-)
UGAGGUAGUAGGUUGUAUAGUUUUAGGGUCACACCCACCACUGGGAGAUAACUAUACAAUCUACUGUCUUUC
>Tcy-Let-7-P2a2 scaffold_45499:620-693(+)
UGAGGUAGUAGGUUGUAUAGUUUGGGGGUCGCUCCCUCUGUCUGUGAGAUAACUAUACAGUCUACUGCCUUUC
>Tcy-Let-7-P2a3 scaffold_80626:226-297(-)
UGAGGUAGUAGAUUGUAUAGUUUUAGGGUCACACCCGAUCUCGGAGAUAACUAUACAGUCUACUGUCUUUC
>Tcy-Let-7-P2b1 scaffold_1:65440882-65440959(-)
UGAGGUAGUAGAUUGUAUAGUUGUGGGGUAGUGAUUUUACCCUGUUCAGGAGAUAACUAUACAAUCUAUUGCC
UUCC
>Tcy-Let-7-P2b2 scaffold_46664:476-553(-)
UGAGGUAGUAGGUUGUGUGGUUUCAGGGUAGUGAUUUUGCCCCAAUCAGAAGAUAACUAUACAACCUACUGCC
UUCC
>Tcy-Let-7-P2c1 scaffold_1:65436490-65436565(-)
AGAGGUAGUAGGUUGCAUAGUUUUAGGGCAGGGAUUUUGCCCACAAGGAGUUAACUAUACAACCUGCUGCCUU
UC
>Tcy-Let-7-P2c2 scaffold_5:79325311-79325388(-)
UGAGGUAGUAGUUUGUGCUGUUGGUCGGGUUGUGACAUUGCCCGCUGUGGAGAUAACUGCGCAAGCUACUGCC
UUGC
>Tcy-Let-7-P2c3 scaffold_1:84118919-84118997(+)
UGAGGUAGUAGUUUGUACAGUUUGAGGGUCUAUGAUACCACCCGGUACAGGAGAUAACUGUACAGGCCACUGC
CUUGC
>Tcy-Mir-1-P1 scaffold_1:506775569-506775630(+)
ACAUACUUCUUUAUGUACCCAUAUGAACAUACAAUGCUAUGGAAUGUAAAGAAGUAUGUAU
>Tcy-Mir-1-P2 scaffold_4:264884848-264884908(-)
ACAUGCUUCUUUAUAUCCCCAUAUGAAUUAUGCUGCUAUGGAAUGUAAGGAAGUGUGUGG
>Tcy-Mir-1-P3 scaffold_2:250585939-250586000(+)
ACAUACUACUUUAUAUGCCCAUAUGAACGUGCUAAGCUAUGGAAUGUAAAGAAGUAUGUAU
>Tcy-Mir-10-P1b-v2 scaffold_3:408052983-408053044(-)
ACCCUGUAGAACCGAAUUUGUGUGGUAUUUACAUAGUCACAGAUUCGAUUCUAGGGGAAUA
>Tcy-Mir-10-P1c-v2 scaffold_4:340923967-340924029(+)
ACCCUGUAGAUCCGAAUUUGUGUAAGGAAUUUUGUGGUCACAAAUUCGUAUCUAGGGGAAUA
>Tcy-Mir-10-P2c scaffold_3:238189117-238189176(+)
AACCCGUAGAUCCGAUCUUGUGGUGAAAUGGACUUCACAAGCUCGUCUCUUUGGGUCUG
>Tcy-Mir-10-P2d scaffold_3:374127422-374127479(-)
AACCCGUAGAUCCGAACUUGUGGUGAUAUUCCACACAAGCUUGUGUCUAUAGGUAUG
>Tcy-Mir-10-P3b scaffold_3:613831400-613831460(-)
UCCCUGAGACCCUUUAACCUGUGAAGACGUCCAGGGUCACAGGUGAGGUUCUUGGGAGCC
>Tcy-Mir-10-P3c scaffold_3:238270556-238270618(+)
UCCCUGAGACCCUAACUUGUGAGAUUUUUUAGCAACAAUCACAGGUCAGGCUCUUGGGACCU
>Tcy-Mir-10-P3d scaffold_3:374078221-374078283(-)
UCCCUGAGACCCUAACUUGUGAUGUUUACCGUUUAAAUCCACGGGUUAGGCUCUUGGGAGCU
>Tcy-Mir-101-P1-v1 scaffold_4:12892887-12892946(+)
UCAGUUAUCACAGUGCUGAUGCUGUCCGUUCUCAAGGUACAGUACUGUGAUAACUGAAG
>Tcy-Mir-103-P1 scaffold_2:600723398-600723458(-)
GGCUUCUCUACAGUGUUGCCUUGUGGCGUGGAGUUCAAGCAGCAUUGUACAGGGCUAUCA
>Tcy-Mir-103-P2 scaffold_2:23705673-23705733(+)
AGCUUCUUUACAGUGCUGCCUUGUUGCAUUGAUGUCAAGCAGCAUUGUACAGGGCUAUGA
>Tcy-Mir-103-P4 scaffold_2:414802677-414802737(-)
GGCUUCUUUACAGUGCUGCCUUGUUGCAUAUGGAUCAAGCAGCAUUGUACAGGGCUAUGA
>Tcy-Mir-122 scaffold_1:362459288-362459346(-)

UGGAGUGUGACAAUGGUGUUUGUGUCCAGUUUAUCAAACGCCAUUAUCACACUAAAUA
>Tcy-Mir-12298 scaffold_2:618087992-618088051(-)
UUUCCAGGAAUUGGGGAUAGCCUGCAUGAAUGCAGAGGUCAUUCCCUAUGCCUGGAGUG
>Tcy-Mir-12316-P1-v1 scaffold_2:23650523-23650587(+)
GGCCUCUGAGGUUCCUUCCAACUCUGAGUUUCUGUGACUGGUUUGAAAGGGAUCAGAGGUCAUU
>Tcy-Mir-12355 scaffold_3:586952075-586952133(-)
GCGGAGGUCCGUUUCCACUCUGAAGUUAUGUGAUUCAGAGCUGGAAUGGACCUCAGAG
>Tcy-Mir-12358-P2-v1 scaffold_3:594178066-594178126(-)
UACCGAUGAGCUCACAGAUCUAUUAUGUCAUGCUUUAAAGAUCUAUGAUUUCGUCGGUGU
>Tcy-Mir-12366-v1 scaffold_6:523503-523558(-)
CCAGCUCUGACAUUCUAUGAUCUACAUACACAGGAUCAAAGGAUUCAGAACUGGA
>Tcy-Mir-12380 scaffold_1:25478402-25478457(-)
UUCCCUCCUACGGCUCUCUGCAGAGGUACCAGAUGCAGAGGGAGGAGGUUGGGAU
>Tcy-Mir-124-P1-v1 scaffold_2:252266640-252266700(+)
CGUGUUCACAGCGGACCUUGAUUUAAUGUCUAUACAAUUAAGGCACGCGGUGAAUGCCAA
>Tcy-Mir-12404 scaffold_7:67032284-67032342(+)
GGUGAAGUGACUUGCCAAGGUUAUUCAUGUGGUAUCUUAAGCAAGUCCCUUCCCCUCU
>Tcy-Mir-1251 scaffold_5:29106475-29106534(-)
ACUCUAGCUGACUAAGGCGCUUCUCUUUCUAAAAAGAGCGCUUUACUUAGCCAGUGUAG
>Tcy-Mir-126-P2-v3 scaffold_2:503100536-503100595(-)
CAUUAUUACUUUUGGUACGCGCUGUGACACAUCAAACUCGUACCGUGAGUAAUAAUGCG
>Tcy-Mir-128-P1 scaffold_1:1943437-1943495(+)
GGGGGCCGUUACACUGUUAGAGAGUGAGUAGCAGGUCUCACAGUGAACCGGUCUCUUU
>Tcy-Mir-128-P2 scaffold_3:350700457-350700513(+)
CGGGGCCGUAGCACUGUCUGAGAGGUUUACAUUUCUCACAGUGAACCGGUCUCUUU
>Tcy-Mir-129-P1 scaffold_6:237621696-237621761(+)
CUUUUUGCGGUCUGGGCUUGCUGUAUAUAACUAUGUAGCCGGGAAGCCCUUACCCCAAAAAGCAU
>Tcy-Mir-129-P2 scaffold_5:180948875-180948938(+)
CUUUUUGCGGUCUGGGCUUGCUGUUCUAAUCAAUAGUCAGGAAGCCCUUACCCCAAAAAGUAU
>Tcy-Mir-130-P1b scaffold_4:360003362-360003425(-)
GCCCUUUUUAUGUUGUACUACUAGUGAUCCUGCACAAGAAGCAGUGCAAUGUUAAAAGGGCAU
>Tcy-Mir-130-P1c scaffold_6:257211561-257211623(-)
GCUCUUUUCACAUUGUGCUACUGUCUGCACCUGUCACUAGCAGUGCAAUGUAAAAAGGGCAU
>Tcy-Mir-130-P2b scaffold_4:360002351-360002413(-)
GCUCUGACUUUAUUGCACUACUGUACUUUACAGCUAGCAGUGCAAUAGUAUUGUCAAAGCAU
>Tcy-Mir-130-P3b scaffold_4:359984350-359984415(-)
ACCCUAUCGAUAUUGUCUCUGCUGUGUAUAUAGCUGUGAGUAGUGCAAUAUUGCUUAUAGGGUUU
>Tcy-Mir-130-P4a scaffold_69449:47-104(+)
GCUCUUUCCCUGUUGCCCUGCUGUGUGUCAGCAAGCAGUGCAAUUGUGAAAGGGCAU
>Tcy-Mir-1306 scaffold_1:731935444-731935505(+)
CCACCUCCCCUGCAAACGUCCAGUGACGCAGACGUAAUGGACGUUGGCUCUGGUGGUGCUG
>Tcy-Mir-132-P2 scaffold_4:451460283-451460345(-)
ACCUUGGCUCUAGACUGCUUACUGCCCGGGCCACCCUCAGUAACAGUCUCCAGUCACGGCCA
>Tcy-Mir-1329 scaffold_2:215678167-215678228(-)
GUACAGUGAUCGGGUUAUGAUGGAUUUCUCAAGUAACCACCUCGUAGCUCGGUCACGAUAU
>Tcy-Mir-133-P1-v1 scaffold_1:506779517-506779576(+)
AGCUGGUAAAAUGGAACCAAAUCACCUAUUCAAUGGAUUUGGUCCCCUUCAACCAGCUG
>Tcy-Mir-133-P2-v1 scaffold_4:264876992-264877052(-)
GGCUGGUCAAACGGAACCAAGUCCGUCUGACUGAGAGGUUUGGUCCCCUUCAACCAGCUA
>Tcy-Mir-133-P3-v2 scaffold_2:250611025-250611084(+)
AGCUGGUAAAAAGGAACCAAAUCACCUGUGCGAUGGAUUUGGUCCCCUUCAACCAGCUG
>Tcy-Mir-135-P2 scaffold_5:29015297-29015357(-)
UAUGGCUUUUUAUUCCUAUGUGAUAGUAAUAAAGUCUCAUGUAGGGAUGGAAGCCAUGAA
>Tcy-Mir-135-P4 scaffold_4:103709005-103709066(-)

UAUGGCUUUUCAUUCCUAUGUGAUUGCUUUUCCUAACUCAUGUAGGGCUAAAAGCCAUGGG
>Tcy-Mir-137-P1-v2 scaffold_4:409130227-409130286(-)
ACGGGUAUUCUUGGGUGGAUAAUACGGAUUACGUUGUUAUUGCUUAAGAAUACGCGUAG
>Tcy-Mir-137-P3 scaffold_3:309372663-309372722(-)
ACGGGUAUUCUUGGGUAGAUAAUACCGAUGGUGCUGUUAUUGCUUGAGAAUACACGUAG
>Tcy-Mir-138-P1 scaffold_2:487928054-487928122(+)
AGCUGGUGUUGUGAAUCAGGCCGACAAGCAGCUCAUCCUAUUACCCGGCUAUUUCACCACACCAGGGU
>Tcy-Mir-138-P2 scaffold_5:276581506-276581567(-)
AGCUGGUGUUGUGAAUCAGGCCGUCGCCCGUCUGAGAACGGCUACUUCACAACACCAGGGU
>Tcy-Mir-1388 scaffold_2:254219414-254219474(-)
AGGACUGUCUAACCUGAGAAUGGUGAAUCUCAAGGUCAAUCUCAGGUUUGUCAGCCCAUG
>Tcy-Mir-139 scaffold_3:517874900-517874959(+)
UCUACAGUGCAUGUGUCUCCAGUGUGACUAAGGGACUGGAGAUACAGCCCUGUUGGAAU
>Tcy-Mir-1397 scaffold_3:216481140-216481198(+)
UGCAUUGCGACGGGUUACAUCACUGAAUUUAGCAUGAUGUAACCCAACGCAGCAUGAU
>Tcy-Mir-140-P1-v1 scaffold_2:45964188-45964249(-)
CAGUGGUUUUACCCUAUGGUAGGUUACGUCAUGCUGUUCUACCACAGGGUAGAACCACGGA
>Tcy-Mir-142-P1-v3 scaffold_4:358906534-358906593(-)
CAUAAAGUAGAAAGCACUACUAACAGCAAUGUAGGGUGUAGUGUUUCCUACUUUAUGGA
>Tcy-Mir-143 scaffold_2:424187300-424187355(-)
GGUGCAGUGCUGCAUCUCUGGUCAGUUGUGAGUCUGAGAUGAAGCACUGUAGCUC
>Tcy-Mir-144 scaffold_4:447593750-447593808(-)
GGAUAUCAUCGUAUACUGUAAGUUUGCAAUGAGACACUACAGUAUAGAUGAUGUACUG
>Tcy-Mir-145 scaffold_2:424184407-424184467(-)
GUCCAGUUUUCCCAGGAAUCCCUUAGGUGCUAAGAUGGGGAUUCCUGGAAAUACUGUUCU
>Tcy-Mir-146-P1 scaffold_2:574663931-574663991(+)
UGAGAACUGAAUUCCAUAGGCUGUGAGCUCAUGCAGAUGCCCUAGGAAGUCAGUUCUGGA
>Tcy-Mir-146-P4 scaffold_2:404913124-404913181(+)
UGAGAACUGAAUUCCAUGGGUUGUCUUUGUAUCAGACCUAUGAAACUCAGUUCUUCA
>Tcy-Mir-147 scaffold_2:275985888-275985948(+)
GUGGAAACAUUUCUGCACAAACUAGACUACUGAAACCAGUGUGCGGAAUUGCUUCUGCUA
>Tcy-Mir-148-P1 scaffold_69470:458-518(+)
AAAGUUCUGUGACGCUCAGGCUCUGAUUAGGAAAGCAGUCAGUGCACUACAGAACUUUGU
>Tcy-Mir-148-P3 scaffold_4:341639640-341639701(+)
CAGGUUCUGUGAUACACUCUGACUUAGACUCUGGAGCAGUCAGUGCAUGACAGAACUUGGG
>Tcy-Mir-148-P4 scaffold_5:90394826-90394887(-)
GAAGUUCUGUUAUACACUCAGACUGUGGCUCUCUGAAAGUCAGUGCAUCACAGAACUUUGU
>Tcy-Mir-15-P1a scaffold_3:538226608-538226667(-)
UAGCAGCACAUAAUGGUUUGUUGGUUUUGAAAAGGUGCAGGCCAUAUUGUGCUGCCUCA
>Tcy-Mir-15-P1b scaffold_3:19896435-19896495(+)
UAGCAGCACAUCAUGGUUUACGUGUUCUUGCCCAGAUGCGAAUCAUUAUUUGCUGCUUUA
>Tcy-Mir-15-P1c scaffold_7:38476109-38476167(-)
UAGCAGCACGCCAUGGUUUGUAGAGAUAAGGUGAUGCAAACCAUCGUGGGCUGUUACA
>Tcy-Mir-15-P2a scaffold_3:538226458-538226523(-)
UAGCAGCACGUAAAUAUUGGCGUUAAGAUUUUAAAAGUAUCUCCAGUAUUAACUGUGCUGCUGAA
>Tcy-Mir-15-P2b scaffold_3:19896603-19896668(+)
UAGCAGCACGUAAAUAUUGGCGUAGUGAAAUAAACCUGAAACCCCAAUAUUAUUGUGCUGCUUUA
>Tcy-Mir-15-P2c-v1 scaffold_7:38475898-38475959(-)
UAGCAGCACGUCAAAACUGGAGUCAUCUACCUAAAUCCUCCAGUAUUGCCUUGCUGCUUGA
>Tcy-Mir-15-P2d scaffold_4:297090589-297090651(-)
UAGCAGCACAGAAAUAUUGGCACCUGAGGGAAAGCCAUGCCAGUAUUGAGAGUGCUGCUCCA
>Tcy-Mir-153-P1 scaffold_5:213520277-213520337(-)
UCAUUUUUGUGAUGUUGCAGCUAGUAAUAUGAGCCCAGUUGCAUAGUCACAAAAGUGAUC
>Tcy-Mir-153-P2 scaffold_3:66542313-66542372(+)

UCAUUUUUGUGAUCUGCAGCUAGUACUAUGGCUUCAGUUGCAUAGUCACAAAAGUGAUC
>Tcy-Mir-1540 scaffold_2:137417066-137417124(+)
AUUCCAUGGAGCAUAUGCUCUGGCUCUCUGGGGAUGGACAUGCGCUCUAUGGAAUCAC
>Tcy-Mir-1541 scaffold_3:622610307-622610366(-)
ACAUCCCAACUGGGCACACUGUUGUGAUGAUGGACAGAUGGUGUGCUUGUUUGGAUGUG
>Tcy-Mir-1542 scaffold_5:18354160-18354221(+)
CUGGCACAUAGGAGCUUCAUAAAUGUGUGUUGAUUUAUUUAUUGAUCUCCAAUGCCUAGCA
>Tcy-Mir-1546 scaffold_1:727160126-727160184(+)
AGGGAUUCUGAGAGGUGGAAGGUAUUUUGUCUCACUUCCAUCCCUGAGAAUCCCUGAU
>Tcy-Mir-1546-as scaffold_1:727160123-727160183(-)
UCAGGGAUUCUCAGGGAUGGAAGUGAGACAAAAUACCUUCCACCUCUCAGAAUCCCUAUU
>Tcy-Mir-1547 scaffold_3:518764375-518764435(+)
AUCAGAGUCUUGGGUCCUUGUGUAGAUGCUUUGAGACUUCAAGGAUCCCUGACUUGAUCA
>Tcy-Mir-1549 scaffold_5:109471784-109471841(-)
GCCGGGCUCGUGGGUAGGAGCUGCGUUUACUUUUGCUUCCGCCCUGCAAGCCCGGUA
>Tcy-Mir-155 scaffold_3:243701259-243701327(+)
UUAAUGCUAAUCGUGAUAGGGGUUUUUCUCUUUUAUUUCUGACUGACUCCUACAUGUUAGCAUUAACA
>Tcy-Mir-17-P1a scaffold_3:143851872-143851933(+)
CAAAGUGCUUACAGUGCAGGUAGUGAUAUGUAGAAUCUACUGCAGUGAAGGCACUUGUAGC
>Tcy-Mir-17-P1c scaffold_7:37925536-37925594(-)
AAAAGUGCUUAUAGUGCAGGUAGGUCGGUGUAACUACUGCCCUGUGAGCACUUCCAAC
>Tcy-Mir-17-P2a scaffold_3:143852012-143852076(+)
UAAGGUGCAUCUAGUGCAGAUAGUGAAGUAGAUUAGCAUCUACUGCCCUAAGUGCUCCUUCUGG
>Tcy-Mir-17-P4a scaffold_3:143852330-143852389(+)
UAAAGUGCUUAUAGUGCAGGUAGUGUUUAGUUAUCUACUGCAUUAUGAGCACUUGAAGU
>Tcy-Mir-17-P4d scaffold_4:295402625-295402685(-)
CAAAGUGCUGUUCGUGCAGGUAGUGAUAACCCAACCUACUGCUGAGCUAGCACUUCCCGA
>Tcy-Mir-1805 scaffold_3:228818942-228819003(-)
AGUUGUAGCUUUUCAAACAGAGCUCUGUAUGUACACACCUGUAUUGGAACACUACAGCUCC
>Tcy-Mir-181-P1a scaffold_4:95246318-95246380(-)
AACAUUCAACGCUGUCGGUGAGUUUGGAAUUAAAAUGAAAACCAUCGACCGUUGAUUGUACC
>Tcy-Mir-181-P1b scaffold_2:461201738-461201800(+)
AACAUUCAACGCUGUCGGUGAGUUUUCAGAUUUGAAAGAAACCAUCGACCGUUGACUGUACC
>Tcy-Mir-181-P1c scaffold_1:667846683-667846746(+)
AACAUUCAACGCUGUCGGUGAGUUUGAGCAGCUGAAGGCAAACCAUCGACCGUUGAGUGGACC
>Tcy-Mir-181-P2a scaffold_4:95246132-95246194(-)
AACAUUCAUUGCUGUCGGUGGGUUUAACUAUGUGGACAAGCUCACUGAACAAUGAAUGCAAC
>Tcy-Mir-181-P2b scaffold_2:461202867-461202927(+)
AACAUUCAUUGCUGUCGGUGGGUUUUUAUCUUACUCAACUCACUGAUCAAUGAAUGCAAA
>Tcy-Mir-181-P2c scaffold_1:667847002-667847066(+)
AACAUUCAUUGCUGUCGGUGGGUUGUAAGAUGUGAGGAAAAACUCACCGAUGGAUGAAUGUCAC
>Tcy-Mir-184 scaffold_2:564635210-564635272(+)
CCUUAUCACUUUUCCAGCCCAGCUUUCUAACUCUAAUUGUUGGACGGAGAACUGAUAAGGGU
>Tcy-Mir-186 scaffold_4:7010295-7010356(+)
CAAAGAAUUCUCCUUUUGGGCUUUAUAUUCUUAAUCUCAGCCCAAAGGUGAAUUUUUUGGG
>Tcy-Mir-187 scaffold_1:393135619-393135677(+)
GGCUACAACACAGGACACGGGAGCUUCUCUGACCCCUCGUGUCUUGUGUUGCAGCCAG
>Tcy-Mir-19-P1a scaffold_3:143852161-143852219(+)
AGUUUUGCAUAGUUGCACUACAAGAAGAAUGUAGUUGUGCAAAUCUAUGCAAAACUGA
>Tcy-Mir-19-P2a scaffold_3:143852462-143852523(+)
AGUUUUGCAGGUUUGCAUCCAGCUGUAUGAUAUUCUGCUGUGCAAAUCCAUGCAAAACUGA
>Tcy-Mir-19-P2c scaffold_7:37924922-37924981(-)
AGUUUUGCAGGUCUUGCAUCGGCCUGUGUCAAUUGCUGUGCAAAUCCAUGCAAAACUGA
>Tcy-Mir-190-P1 scaffold_2:538720458-538720517(-)

UGAUAUGUUUGAUAUAUUAGGUUGUAAUUUAAUCCAACUAUAUAUCAAACAUAUUCCUA
>Tcy-Mir-190-P3 scaffold_4:366107052-366107112(-)
UGAUAUGUUUGAUAUUAGGUUGUUUGAUUGGAAAUCAACCAAAUGUCAAACAUAUUCUUA
>Tcy-Mir-191 scaffold_1:89426481-89426545(+)
CAACGGAAUCCCAAAAGCAGCUGUUGUCUUCAGAGCAUUCCAGCUGCAAUUGGAUUUCGUUCCC
>Tcy-Mir-193-P1a scaffold_1:311935970-311936029(+)
CGGGGUUUUGAGGGCGAGAUGAGUUUAUAUUUUAUCCAACUGGCCCACAAAGUCCCGCU
>Tcy-Mir-193-P1b scaffold_4:445349888-445349944(-)
UGGGUCUUUGCGGGCGAGAUGAGGGUGUCAAUUCAACUGGCCUACAAAGUCCCAGU
>Tcy-Mir-193-P2a scaffold_1:311941583-311941645(+)
AGGGACUUUUGGGGGCAGAUGUGUUUCCAUUACACUAUCAUAAUGCCCCUAAAAAUCCUUAU
>Tcy-Mir-193-P2b scaffold_4:445324137-445324199(-)
AGGGACUUUCAGGGGCAGCUGUGUUUUAUUAACUCAGUCAUAAUGCCCCUAAAAAUCCUUAU
>Tcy-Mir-194-P2 scaffold_6:250976414-250976472(+)
UGUAACAGCAACUCCAUGUGGGACGGCUUCCUUCUUCCAGUGGGGAUGCUGUUACUUU
>Tcy-Mir-196-P1 scaffold_5:280645531-280645590(+)
UAGGUAGUUUCCUGUUGUUGGGGCUCCACCUUUCUCUCGACAGCAUGAUACUGCCUUCA
>Tcy-Mir-196-P3 scaffold_4:340860600-340860660(+)
UAGGUAGUUUCCUGUUGUUGGGCCUAGAUUUCUAAACACAAGAACAUUAAACCACCUGAU
>Tcy-Mir-196-P4 scaffold_5:90906858-90906917(-)
UAGGUAGUUUCAUGUUGUUGGGAUUGAGUUUUGAACUCGGCAACAAGAAACUGCCUGAG
>Tcy-Mir-199-P1 scaffold_4:62820199-62820260(-)
CCCAGUGUUCAGACUACCUGUUCAGGACAAUGCUGUUGUACAGUAGUCUGCACAUUGGUUA
>Tcy-Mir-199-P2 scaffold_2:493193323-493193384(-)
CCCAGUGUUUAGACUAUCUGUUCAGGACUCCAAUAUUGUACAGUAGUCUGCACAUUGGUUA
>Tcy-Mir-199-P3 scaffold_1:665742117-665742180(-)
CCCAGUGUUCAGACUACCUGUCCAGGAGAUUGCAAAUGUGUACAGUAGUCUGCACAUUGGUUA
>Tcy-Mir-202 scaffold_2:682862326-682862384(-)
UUCCUAUGCAUAUACUUCUUUGAGAAUAAAUUCUAAAGAGGCGUAGGGCAUGGGAAAA
>Tcy-Mir-203-v1 scaffold_2:364523980-364524040(-)
AGUGGUUCUUAACAGUUCAACAGUUCUAUUAAAAAAUUGUGAAAUGUUUAGGACCACUCU
>Tcy-Mir-204-P1 scaffold_2:543792758-543792816(+)
UUCCCUUUGUCAUCCUAUGCCUGGAAAUCAGAGUGGGGCAGGGACAGCAAAGGGAUGC
>Tcy-Mir-204-P2 scaffold_1:299067184-299067244(-)
UUCCCUUUGUCAUCCUAUGCCUGAGAAUAUAUGAAGGGGGCUGGGAAGGCAAAGGGACGU
>Tcy-Mir-205-P4 scaffold_4:108588378-108588437(+)
UCCUUCAUUCCACCGGAGUCUGUCUCAUAUCUAAUCAGAUUUCAGUGGAGUGAAGCAUA
>Tcy-Mir-208-P1 scaffold_2:508748315-508748372(-)
AAGCUUUUUGCUCGCAUUAUAUUUUGGAUUUGAAUAUAAGACGAACAAAAGGUUUGU
>Tcy-Mir-208-P2 scaffold_2:508701900-508701957(-)
GAGCUUUUGGCUCGGGUUAUACCUGAUGCAUGUGUAUAAGACGAGCAAAAAGCUUGU
>Tcy-Mir-21 scaffold_4:360764677-360764737(+)
UAGCUUAUCAGACUGAUGUUGACUGUUGGAUCUCAUGGCAACAGCAGUCGAUGGGCUGUC
>Tcy-Mir-210 scaffold_6:242970947-242971005(-)
AGCCACUGACUAACGCACAUUGUGCUGAGGGACCCACUGUGCGUGUGACAGCGGCUAC
>Tcy-Mir-214-v1 scaffold_4:62814138-62814201(-)
UGCCUGUCUACACUUGCUGUGCAGAACAUCCUCUCACCUGUACAGCAGGCACAGACAGGCAGU
>Tcy-Mir-216-P2a scaffold_2:103425330-103425392(-)
AAAUCUCUGCAGGCAAAUGUGGUGUUGCUAUAGUUAUCACACAAUUACCUGUAGAGAUUCUG
>Tcy-Mir-216-P2b scaffold_2:103408050-103408112(-)
UAAUCUCAGCUGGCAACUGUGAGAUAUUAAUAAAUUCCCUCACAGUGGUCUCUGGGAUUAUG
>Tcy-Mir-217-v2 scaffold_2:103398219-103398278(-)
UACUGCAUCAGGAACUGAUUGGAUAAUAUUCAGGUACCAUCAGUUCCUAAUGCAUUGCC
>Tcy-Mir-218-P1 scaffold_2:414998177-414998241(-)

UUGUGCUUGAUCUAACCAUGUGGUGGAAUGAUAGAAACAGAACAUGGUUCUGUCAAGCACCGCG
>Tcy-Mir-218-P2 scaffold_6:11711786-11711850(+)
UUGUGCUUGAUCUAACCAUGUGGUUGUGAGGUAUGAGUAAAACAUGGUUCUGUCAAGCACCAUG
>Tcy-Mir-219-P3 scaffold_4:292255955-292256015(-)
UGAUUGUCCAAACGCAAUUCUCGUGGCUCCGGCCCUUGAGAGUUGGGUCUGGACAUCUCG
>Tcy-Mir-22-P1b scaffold_4:451068511-451068571(-)
AGUUCUUCAGUGGCGAGCUUUAUGUCUUGUCCCAGCUAAAGCUGCCAGUUGAAGAACUGC
>Tcy-Mir-221-P1a scaffold_3:254755142-254755206(+)
UGCUCAGUAGUCAGUGUAGAUCCUGUCCCUUUCAAUCAGCAGCUACAUCUGGCUACUGGGUCUC
>Tcy-Mir-221-P2a scaffold_3:254755726-254755790(+)
AACCUGGCAUACAAUGUAGAAUUCUGUGUUUAUUAAGUAACAGCUACAUUGUCUGCUGGGUUUC
>Tcy-Mir-223 scaffold_4:400568613-400568678(+)
CGUGUAUUUGACAAGCUGAGUCGGACACUCCAUGUGGUAGAGUGUCAGUUUGUCAAAUACCCCAA
>Tcy-Mir-23-P2 scaffold_1:234877964-234878024(-)
GGGUUCCUGGCAUGCUGAUUUGUGACUUAAGAUUAAAAUCACAUUGCCAGGGAUUACCAC
>Tcy-Mir-23-P3 scaffold_1:667771300-667771359(-)
GGGGUUCCUGGGGAUGGGAUUUGAUUACUGCCACAAAUCACAUUGCCAGGGAUUUCCAA
>Tcy-Mir-24-P2 scaffold_1:234877205-234877264(-)
GUGCCUACUGAGCUGAUAACAGUUCUGAUUUUACACACUGGCUCAGUUCAGCAGGAACA
>Tcy-Mir-24-P3 scaffold_1:667770975-667771035(-)
GUGCCUACUGAGCUGAAACACAGUUGCUUUGAAUAAACUGGCUCAGUUCAGCAGGAACAG
>Tcy-Mir-26-P1 scaffold_1:3341455-3341516(+)
UUCAAGUAAUCCAGGAUAGGCUGGGCCCAUUCUCAUUGGCCUAUUCUUGGUUACUUGCACU
>Tcy-Mir-26-P2 scaffold_3:67425585-67425642(-)
UUCAAGUAAUCCAGGAUAGGCUGUUUUCUCUUUAGCCUGUUCUCCAUUACUUGGUUC
>Tcy-Mir-26-P4 scaffold_5:85533642-85533701(+)
UUCAAGUAAUCCAGGAUAGGCUGUGUCCAGCUGCAGGCCUAUUCUUGAUUACUUGUUUC
>Tcy-Mir-27-P2 scaffold_1:234877749-234877812(-)
AGAGCUUAGCUGAUUGGUGAACAGUCAUUGAUUUCCUCUUUGUUCACAGUGGCUAAGUUCUGC
>Tcy-Mir-27-P3 scaffold_1:667771115-667771178(-)
AGGACUUAGCUGCCUUGUGAACAGAGUCAGCAUCAUAUUGUGUUCACAGUGGCUAAGUUCCGC
>Tcy-Mir-28-P1 scaffold_1:510516533-510516597(+)
CAAGAGUUUACAAUUUAGUUGGGAAGAUUGUCUUACCUCCUCAACUAGAUUAUAAACUCCUUGA
>Tcy-Mir-29-P1b scaffold_5:183305440-183305503(-)
GCUGGUUUCAUAUGGUGGUUUAGAUUUAACUACUGAGUGUCUAGCACCAUUUGAAAUCAGUGU
>Tcy-Mir-29-P1d scaffold_4:106413633-106413697(-)
GCUGGUUUCACAUGGUGGCUUAGAUUUUUCCAUCUCUGUAUCUAGCACCAUUUGAAAUCAGUGU
>Tcy-Mir-29-P2b scaffold_5:183305026-183305086(-)
ACUGAUUUCUUUUGGUGUUCAGAGUCAAUAUCAUUUUCUAGCACCAUUUGAAAUCGGUUA
>Tcy-Mir-29-P2d scaffold_4:106412873-106412931(-)
ACCGAUUUCUCUUGGUGUUCAGAGUCUGUUUUUGUCUAGCACCAUUUGAAAUCGGUUA
>Tcy-Mir-2970 scaffold_1:89429050-89429109(+)
UGCAGUCAGUAGUUGGUCUGGCGUGAGGAGGAAUUCUCAGAUCACCUCUUGGCUGUGAG
>Tcy-Mir-30-P1a scaffold_1:642724192-642724254(-)
UGUAAACAUCCCCGACUGGAAGCUGUAAGUCACAGCCAAGCUUUCAGUCAGAUGUUUGCUGC
>Tcy-Mir-30-P1b scaffold_4:245351114-245351178(+)
UGUAAACAUCCUCGACUGGAAGCUGUGAAGCAGCAGAUGGGGCUUUCAGUCGGAUGUUUGCAGC
>Tcy-Mir-30-P1d scaffold_3:561198176-561198240(+)
UGUAAACAUCCUUGACUGGAAGCUGUAAGGUGCUUGGAGGAGCUUUCAGUCGGAUGUUUACAGC
>Tcy-Mir-30-P2a scaffold_1:642718276-642718336(-)
UGUAAACAUCCUACACUCAGCUGUAACACAUGGAUUGGCUGGGAGGUGGAUGUUUACUUC
>Tcy-Mir-30-P2b scaffold_4:245399836-245399898(+)
UGUAAACAUCCUACACUCUCAGCUGUGGAAAGUGAAAAAGCUGGGAGAAGGCUGUUUACUCU
>Tcy-Mir-30-P2d scaffold_3:561201497-561201558(+)

UGUAAACAUCCUACACUCUCAGCUGUGAGCUCAAGGUGGCUGGGAGAGGGCUGUUUACUCC
>Tcy-Mir-31 scaffold_1:296416227-296416286(+)
AGGCAAGAUGUUGGCAUAGCUGUUGAUAUAAGAACCUGCUAUGCCAACAUAUUGCCAUC
>Tcy-Mir-32 scaffold_1:341087745-341087807(-)
UAUUGCACAUUACUAAGUUGCAUGUUGUCACGGCCUUAGUGCAAUUUAGUAUGUGUGAUAUU
>Tcy-Mir-325 scaffold_2:338341763-338341823(-)
CGUAGUAGGUGCUUAAUAAAUGCUUGUUGAUUGAUUGGUUGAUUAAGCACCUACUCAGUG
>Tcy-Mir-33-3P scaffold_1:240628769-240628826(-)
GUGCAUUGCAGUUGCAUUGCGUGUAUCUGUCCGGCGCAAUGCCCCAGCAAUGCAGUA
>Tcy-Mir-338-P2 scaffold_4:314635498-314635559(-)
AACAAUAUCCUGAUGCUGAGUGAGCGGCACACAGAGACUCCAGCAUCAGUGAUUUUGUUGA
>Tcy-Mir-34-P1 scaffold_2:554551941-554552007(+)
UGGCAGUGUCUUAGCUGGUUGUUGUGAGUAAUAGAUGAGGAAGCAAUCAGCAAGUAUACUGCCCUA
>Tcy-Mir-34-P2a scaffold_3:501721996-501722055(+)
AGGCAGUGUAGUUAGCUGAUUGUAUUCUACUGCCUACAAUCACUAACUCCACUGCCAUC
>Tcy-Mir-34-P2b scaffold_3:501722691-501722749(+)
AGGCAGUGUAGUUAGCUGAUUGCUAGUAGUAGUACCAAUCACUAACCACACAGCCAAG
>Tcy-Mir-34-P3c scaffold_1:148947599-148947660(-)
UGGCAGUGUAUUGUUAGCUGGUUGAAUAUCUGAAUGCACCAGCUAACAUGCAACUGCUGUC
>Tcy-Mir-34-P3d scaffold_1:148946345-148946406(-)
GGGCAGUGUAUUGUUAGUUAGCUGUUUUUCUUAUUUUCCAGCAACUGCAUACACUUCCACA
>Tcy-Mir-340-v2 scaffold_2:113436021-113436078(+)
CUAUAAAGUAAUGAGACUGGUGUUUGUGUGCAGGAUCAGUCUCAUUACUUUAUAGUC
>Tcy-Mir-3613 scaffold_3:538174635-538174694(-)
UGUUGUACUUUUUUUUUUGUUCGUUUUAUUUUUAGGAACAAAAAAAAAAGCCCAACCCU
>Tcy-Mir-375 scaffold_3:66883894-66883951(+)
GCGCCGAGCCCCUCGCACAAACCGGACCUGAACGUUUUGUUCGUUCGGCUCGCGUGA
>Tcy-Mir-383-v2 scaffold_6:185159518-185159581(+)
CAGAUCAGAAGGUGAUUGUGGCUUUGGGCAGACAUGGAACAGCCACAUCACUGGCUGGUCAGA
>Tcy-Mir-425 scaffold_1:89428595-89428658(+)
AAUGACACGAUCACUCCCGUUGAGCGGACAGCCAAGAAGCCAUCGGGCAUAUCGUGUCUGUCC
>Tcy-Mir-430-P1 scaffold_6:129570855-129570916(+)
ACUUUAACAUGGAGGUACUUUCUGUAACUAAAAAAAAGUAAGUGCUUCCAUGUUUUGGUAG
>Tcy-Mir-430-P2 scaffold_6:129571004-129571064(+)
GCUUUAACAUGGGGGUACCUGCUUCAUAAUUAAAAAGUAAGUGCUUCCAUGUUUCAGUGG
>Tcy-Mir-430-P3 scaffold_6:129571208-129571269(+)
ACUUAAACGUGGAUUUACUUGCUUUGUUUCUAAAAAAGUAAGUGCUUCCAUGUUUUAGUGA
>Tcy-Mir-430-P4 scaffold_6:129571378-129571440(+)
CUUUUAACAUGGAAUUGCUUGCUAUGGUUUUAAAAAAAUUAAGUGCUUCAUUGUUUGAGUUG
>Tcy-Mir-430-o11 scaffold_3:617160487-617160546(-)
AUCCUAACUUGGAGCACUUAUUGUAUAUGUAGAACCAUAAGUGCCCCCUGUUAGGAUGA
>Tcy-Mir-430-o13 scaffold_3:617162754-617162813(-)
AUCCUAACUCAGAGCACUUAUUGGACUUAUAUAAGAAUAAGUGUUCUCUGUUAGGAUGA
>Tcy-Mir-430-o16 scaffold_3:617163381-617163441(-)
AUCCUAAUUUAGAACACUUACUGGAUAUGUAAGAAUCAUAAGUGCUCUCUGUUAGGAUGA
>Tcy-Mir-430-o18 scaffold_5233:661-721(-)
AUCCUAAUUCGGAGCACUUACUGGAUAUGUAAGAACCAGAAGUGCCCUCUGUUAGGAUGA
>Tcy-Mir-430-o19-v1 scaffold_3:269897771-269897830(-)
GCCCUGACUGAGGGCAUUUAUUGGAUAUUUGGAAAGAUAAGUGCUCUCUGUUGGGGUGA
>Tcy-Mir-430-o19-v2 scaffold_3:617159799-617159858(-)
ACCCUAACUGAGGGCAUUUAUUGGAUAUUUGGAAAAAUAAGUGCUCUUUGUUGGGGUGA
>Tcy-Mir-430-o19-v3 scaffold_5233:366-425(-)
ACCCUAACUGAGGGCAUUUAUUGAAUAUUUGGGAAGAUAAGUGCUCUCUGUUGGGGUGA
>Tcy-Mir-430-o19-v4 scaffold_3:617172444-617172503(-)

ACCCUAACUGAGGGCAUUUAUUGGAUAUUUGGAUAGAUAAGUGCUCUCUGUUAGGGUGA
>Tcy-Mir-430-o19-v5 scaffold_3:617161923-617161982(-)
ACCCCAACUGAGGGCACUUAUUGGAUAUUUGGAAAGAUAAGUGCUCUCUGUUGGGGUGA
>Tcy-Mir-430-o23-v1 scaffold_31303:495-555(-)
AUCCUAACUGAGAGCACUUACAGGAUUUGUAAGAAUCAUACGUGCUCUCUGUUAGGACAA
>Tcy-Mir-430-o23-v2 scaffold_5233:3223-3284(-)
AUCCUAACUCAGAGCACUUACUGGAUAUGUUAGCACCAUUAAGUGCCUUCUGUUGGGAUGA
>Tcy-Mir-430-o23-v3 scaffold_5233:1701-1761(-)
AUCCUAAUUCAGAGCACUUCUAGGAUUUGUAAGAACCAUACGUGCUCUCUGUUAGGAUGA
>Tcy-Mir-430-o23-v4 scaffold_5233:3738-3798(-)
AUCCUAACUCAGAGCACUUAUAGGAUUGGUAAGAACCAUAAGUGCUCUCUGUUAGGAUGA
>Tcy-Mir-430-o24-v1 scaffold_5233:1202-1262(-)
AUCCUAACUCAGGGCAUUUAUUGGGUUUUUGUGAUAAAUAAGUGCUCUGAGUUGGGAUGA
>Tcy-Mir-430-o24-v2 scaffold_31303:648-706(-)
AUUCUAAUUGAGAUCAUUUGUUGGUUGUUUGAGGAAUAAGUGCUCUCAAUUGGGGUAG
>Tcy-Mir-430-o26-v1 scaffold_3:617162068-617162128(-)
AUCCUAACUCCUGGCAUUUAUUGGGUUUUUGUGACAAAUAAGUGCUCUGAGUUGGGAUGA
>Tcy-Mir-430-o26-v2 scaffold_31303:335-396(-)
AUCCUGACUCCAGAGCAUUUAUUGAAUAUUUAUAAAAGAUAAGUGCUCUCUGUUGGGAUGA
>Tcy-Mir-430-o29 scaffold_5233:896-956(-)
AUCCUAACUCAGAGCACUUAUUGGAUAUAUAAGAAUGAUAAGUGUUCUCUGUUAGGAUGA
>Tcy-Mir-451 scaffold_4:447593625-447593667(-)
AAACCGUUACCAUUACUGUGCUUAGUAAUGGUAAGGGUUCUC
>Tcy-Mir-455 scaffold_2:490820055-490820113(-)
UAUGUGCCCUUGGACUACAUCGUGGAAGCCAGCACCAUGCAGUCCAUGGGCAUAUACA
>Tcy-Mir-460-P1 scaffold_1:93777702-93777760(+)
CCUGCAUUGUACACACUGUGUGUUUUCACUACUAUGCACAGCGCAUACAAUGUGGAUA
>Tcy-Mir-499 scaffold_2:433455885-433455944(+)
UUAAGACUUGCAGUGAUGUUUAAUUCUUCUCUACGUGAACAUCACAACAAGUCUAUACA
>Tcy-Mir-551-P1 scaffold_3:600182720-600182780(+)
GAAAUCCAGAAGGGGUGGAGCCUGUUGGACAGUUUCUAGGCGACCCAUUCUUGGUUUCAA
>Tcy-Mir-551-P2 scaffold_3:28096251-28096312(+)
GAAAUCAAGGAUGGGUGAGACCUCGUGUGCAAACUGAAAGGCGACCCAUACUUGGUUUCAG
>Tcy-Mir-599 scaffold_1:596989874-596989932(-)
UUUGAUAAGCUGACAUGGGACAGGGUUCUUUUCACUGUUGUGUCAGUUUAUCAAACCC
>Tcy-Mir-671 scaffold_5:205542848-205542908(+)
AGGAAGCCCUGGAGGGGCUGGAGGUGCUCGACGUUUUUCUACGCUUCUCAGGGCUGCACC
>Tcy-Mir-675 scaffold_6:244195430-244195488(-)
CGGAGCGGAGAAAGCAAACAGUGGGGGCUCCAAGCACUGUCUGCUUUAUCCGCUCCUG
>Tcy-Mir-7-P1 scaffold_2:561789436-561789500(+)
UGGAAGACUAGUGAUUUUGUUGUUCUCUAACGUAAAUAUUGACAACAAAUCCCAGUCUGCCUUA
>Tcy-Mir-7-P2 scaffold_1:329766918-329766982(+)
UGGAAGACUAGUGAUUUUGUUGUUUUUAGAUAACUAAAACGACAACAAAUCACAGUCUGCCAUA
>Tcy-Mir-7-P4 scaffold_1:672401061-672401124(-)
UGGAAGACUAGUGAUUUUGUUGUUCUAAUUUAUGAAAAAGACAACAAAACACAGCCUGCCUUA
>Tcy-Mir-7244 scaffold_2:181193902-181193958(+)
AUGGGAAAAUCAAGUGAAAAUGUGAUAUAUAUUUUUUCACCUGUUCUUCCUCUAUU
>Tcy-Mir-7246 scaffold_2:22549495-22549555(-)
UAUCCAACCUUACUUCCCACUACGUGUACUAGAAGAGUGAUGGAAACUAAGGUUGGAUAG
>Tcy-Mir-7247 scaffold_2:646526473-646526529(-)
UGGGAGAACGUGUGUGGGGGAAUGCCGAAGUGGUUUCGCGACAUGCGGUCUCUCAU
>Tcy-Mir-7248 scaffold_2:531349009-531349066(-)
CAUAGAGCGUGGACUGGGUGUGCUGGCACACCCAGGCACAACCGGACCACACUCUGC
>Tcy-Mir-7251 scaffold_2:413951718-413951780(+)

CUAAGCUGGGUGGAUAGUCAAUGCUAACUAUCCCAAUGCUGACUAUCUACUUAGCUUAGUCC
>Tcy-Mir-7254-v1 scaffold_2:442656338-442656398(+)
CACCACCUCCUUGAAUCCUGUUCUAAUGUGAGUGAUGCAGAGUUCAGGGUGGAUGUGGCC
>Tcy-Mir-7257 scaffold_2:670259446-670259504(-)
ACAUGAACCAAUACUUCUAGUUUAGUUUUUAUAUAGAUUGAAAGUAUUGGUUCUUGUU
>Tcy-Mir-7262-P1 scaffold_4:392909241-392909295(+)
AAGUUUUAUUGCAGUUUUAAGCUUUAGAAACUUACAACGGCAGUAAGACUUUUA
>Tcy-Mir-7262-P2 scaffold_4:43720525-43720584(-)
CCAAAAGUCUUAGUACAGUUUUAAGAUAUUAAAGCCUAAAACUGGACCAAGGCUUUUGG
>Tcy-Mir-7262-P3 scaffold_5:276393387-276393440(-)
AAGUCUCAGUGCAGCUAGAAGUUUUUAUCCGCCUCAAGCAGCACUAAGACUUU
>Tcy-Mir-7262-P6 scaffold_7:29568249-29568307(-)
CAAAAGCCUUAGUACGGUUUUAAACCGUUAAGUCUUAAAACUGUGCAAAGGCUUUUGG
>Tcy-Mir-7262-P7 scaffold_6:614495-614553(+)
AAAAGUUUUAGUGUAGUUUUAAGUUUAUUAAAGCUUAAACUGCUCUAAGACCUUUGGG
>Tcy-Mir-7281 scaffold_4:23268212-23268271(+)
AAUAGGCUCUUAGUAAAUGUGUGUUAAAUUGAACAACCAUUUAUGAAGUGCCUAUUAUG
>Tcy-Mir-7284 scaffold_4:8248358-8248419(-)
ACUAGCAUAUAGUUGGUAAUUUAUAAAUGUUUCCUAACUGAUUACCAACUAUACGCCAGUG
>Tcy-Mir-7286-v1 scaffold_1:639304718-639304778(-)
GAGGAGCUUACAUUCUACUGGGGGAGUUCUUGGAACCUAUUUUAGAUUAUAAGCUCCUGG
>Tcy-Mir-7286-v2 scaffold_4:411426043-411426124(-)
UAGGAGCUUACAUUCUAAUAGAAGAAAUAAAGAAACCUUAAGCACUUCUUUGUCUCUUCCAUUAGAAUGUAAG
CUCCUAGA
>Tcy-Mir-7292 scaffold_2:566906867-566906929(+)
AGGAAGGGCCUCACUUUCAAGCUUCUAUAUCCUUGUAGCUGCUAGGAGUUCCUUCCUUCUCC
>Tcy-Mir-7293 scaffold_1:594737203-594737259(+)
GCAGAAAUGCCCCAGCUCCCAGUGGUAAUAGACAGGCGCUGGGGAAUUUCUGCCAC
>Tcy-Mir-7303-P1-v1 scaffold_3:622610921-622610979(-)
UCAUCCAAACUGGACGCACUGUCCCAUAUGAGCAGGAUAUGCGUCCUCUUUGGAUUUU
>Tcy-Mir-7303-P2 scaffold_3:622609455-622609509(-)
ACAUCCAAAGUAGACGCACUCUCUGGUCCUAGACAGGCGUCUUCUUUGGGUGUG
>Tcy-Mir-7304 scaffold_3:622610451-622610509(-)
AUUCAUACCACACUCUCAUUCUUAUCUGUAUCUAGAAUGGGGUGUCCUGGUGUGAAUG
>Tcy-Mir-7309 scaffold_3:573296327-573296386(-)
AGGGAUGGGGAGCUCAGUCAGUGUGUUCCUCCAUUCACUCAAUGUGUUCCCCCUCUCAG
>Tcy-Mir-7318 scaffold_6:168705950-168706008(-)
CCUCAGAUUUCUAUACGCAUUUGUAUUCUCUACAUGAAUGCGUAUAGAAAUCUGAGGC
>Tcy-Mir-7319 scaffold_6:226719961-226720022(+)
CCCUCUCUGGGCCUCCUGUUUCCUCAUCUGUAAAAUGGGAAAGUUGGACUAGAGAUGGUGU
>Tcy-Mir-7323 scaffold_6:94576333-94576395(+)
UACAGAAUUGUGUGACAGGUUGUGCUUAGUGUUAAAACAGCUGUCACCUGGGUUCUGUAACC
>Tcy-Mir-7327 scaffold_1:53667585-53667645(+)
GAGGGGUUGAAGACUCGCCCCCUAACCUGACUCAGAGGGGCGAUUUUACAACCCUUCCCA
>Tcy-Mir-7332 scaffold_1:25505808-25505870(-)
CAAUAGCCUGGAUGAUUCAGAUGAAAGAAUCCACUUGGCCUCCUGAAUCAACUGACUGUUGG
>Tcy-Mir-7333 scaffold_1:25615154-25615211(-)
AAUUAUAUUCAGUCAACAGACCCUUUUAUACAUGAGUCUGUUGCCUGAAUAUAGUCA
>Tcy-Mir-7336 scaffold_3:163795826-163795885(-)
UGUGGAUGCAAAGUGUGCGAUGCUGCUAUAUGAAACGCCGCACACUACUGAAUCCUCAG
>Tcy-Mir-7363 scaffold_7:19518798-19518856(-)
AUCACAGAUCCAUUUGGGUAUUUUAGCAUGUGAAGAAGUACCCAAAGGAUCUGUGAUU
>Tcy-Mir-7368 scaffold_7:9341885-9341945(+)
GCGUAGGAGAAGGGCUGUGGAUCUGUAACUGAGAUGGUCACCAGCUCACCUUCUGCCUAG

>Tcy-Mir-7370 scaffold_7:50281079-50281137(-)
AUUCACACCAGAUGCUCCCUUUUGUGAAUUCUGUCAAAAGGGAUAUCCUGUGUGAAAG
>Tcy-Mir-7371-P10 scaffold_7:51798017-51798073(+)
GUGGAAAAGGUCCUCCAUUUUGUGGAUUCUUCCAAAAAGAGGCCCUUGUCCAUAGA
>Tcy-Mir-7371-P11a scaffold_7:51798506-51798565(+)
ACGUGGAUAAGGCAUUCUAUUUUGUGGAUUUUGCCAAAAUAAUCACUUUAUCCACGGAG
>Tcy-Mir-7371-P13 scaffold_7:51800487-51800545(+)
AUUGUACAAGGUGUUCCAUUUUGUGGUUUUCAUUCAAAGGAAUAUCUUGUAUAAUGGA
>Tcy-Mir-7371-P14 scaffold_7:51801134-51801192(+)
GUUGUACCAGAUGUUACCUUUUAUAGAUUGCUAAAAAAGGUACUUCCUGUGCAAUAGC
>Tcy-Mir-7371-P20 scaffold_7:51810045-51810103(+)
GCUGUACUAGAUGCUGCAUUUUAGAGAUUGUGUGAAAUGUUGCAUCUUGUGCACUAGU
>Tcy-Mir-7371-P22 scaffold_7:51814953-51815009(+)
GUGGACCAUGUUCUUCAAUUUGUAGAUUUUGUACAAAGAAGUAUUUGGUCCAUAGA
>Tcy-Mir-7371-P27 scaffold_7:51824959-51825015(+)
UUGUACCAGAUGCUCCAUUUUCUAGAUUUUAUGAAACGUUGCAUCUUGUAUAACAG
>Tcy-Mir-7371-P29 scaffold_7:51817106-51817164(+)
CUUGGGUAAGGCACUCUUAUUUUGUGAAUUUUACCAAAAUAAAUUACCUUAUCCUUGG
>Tcy-Mir-7371-P39 scaffold_7:51861686-51861743(+)
AUUGUACCCGAUACAACGUUUUAUACAGUAUGAAGAAAGUUGUAUUAUGUACAAUAG
>Tcy-Mir-7371-o10 scaffold_7:51805729-51805788(+)
ACUUGGAUGAGGCGCACUAUUUUGUGAAUCCUACCAAAAUAAUGACCUUAUCCAAGGAG
>Tcy-Mir-7371-o11 scaffold_7:51807213-51807270(+)
UUUGGACAAGGUGCUCUAUUUUGUGGAUUCUCCCAAAAAGAGCAUCUUGUCCAUAGA
>Tcy-Mir-7371-o26 scaffold_7:51858988-51859047(+)
GUUGUACAGAUGUUACAUUUUGUAUCUUAUGAUAAAAUAGUCCCAUCAUGUGCAACAGU
>Tcy-Mir-7371-o7 scaffold_7:51798816-51798871(+)
GCUGGAUAAGGUACUCUAUUGUACAUGCUGUCAAAAUGAGCACCUUAUCCAUAGA
>Tcy-Mir-7373-P1a scaffold_7:41755493-41755550(-)
AAGCCCUUUUUCUGGUAGUUGAGUGAUUUUGCACAACUGCUGCUAAUUAAAGGCUUG
>Tcy-Mir-7373-o1 scaffold_7:41759418-41759472(-)
AAGCCCUUGACAGUAUAUUAGAUAAAUCUAAAUCUAAUAUAAGGAAAGGGGUUG
>Tcy-Mir-7373-o2 scaffold_7:41769888-41769942(-)
AAGCCCUUCUGUUAGUAGUUGAGUUAUCUUACUCAGCUACGAAUGAAGGGCGUG
>Tcy-Mir-7373-o4 scaffold_7:41762336-41762390(-)
AAGCCCUUACCAUUUGGUCUGAUAGAUGUCACUCAGAUCAAAAAGAAGGUAUUG
>Tcy-Mir-7373-o6 scaffold_7:41763205-41763260(-)
AAGUCCUUACUCCACUAACUUAUAGAUUUUUAUCUGUUAGAGGAGGAAGGAAUAG
>Tcy-Mir-7373-o8g scaffold_69402:10-67(+)
AGCCCUUACCCAAAUCUUUGAUGAGUGUUUGUUAAUCAAAGAGGAAGGAAGGGUUGC
>Tcy-Mir-7373-o9c scaffold_7:41762616-41762673(-)
AAGCUCUUACCCCACUGGUUAAUAGGUGUUAAUCUGUUAGGGUGGGGGAAGGGGCUA
>Tcy-Mir-7377 scaffold_7:26847509-26847566(+)
AGAGGUCACCCAGGAAGGAGAUGUAGAAAAAGUGCAUCAUCCUGGCUGACUUCUGCU
>Tcy-Mir-7378 scaffold_7:75956539-75956597(-)
GUGGGGCAUCUUCCAUGGAACUGGAUAUGCACUGUAGCCCCUUAGGAGAUGUCCCAGU
>Tcy-Mir-7379 scaffold_7:75972357-75972413(-)
UAUAUCACCCAUACAUCCAGAUAUAAUUGAAAAAUCUGGAUGCUUUGAUGGUGUAG
>Tcy-Mir-7382 scaffold_7:80355694-80355752(+)
GAGGCUCCUUUCUCGAGUGGGAUAGUCUGAGCAACCCACUAGGGGGAGAAGUUUCUGU
>Tcy-Mir-7391 scaffold_7:53110864-53110922(-)
CCGUGGUAUGGUUAGAAAGCUGCCUUGAUCAUAACGGCAGCUUGCUGCCAUCCAUGUA
>Tcy-Mir-7392-P1 scaffold_54797:190-245(+)
ACGAGUAUACCAUGGUUACCUGAGUGUAUUUUGGUAACCUCAGGAUACUCGUUCC

>Tcy-Mir-7394-P1 scaffold_7:44665715-44665768(+)
AUGUGUGUCCAUGUUAUAUGUGUUUCUUUACAUGUAACCUGGUCACACAUAUU
>Tcy-Mir-7394-P2 scaffold_7:44674144-44674197(+)
CACAUAUGCCCAGGUGACAUGCUGUUCUACAUGUGACCUCAACAUAUGUGUUU
>Tcy-Mir-7394-P3 scaffold_7:44680335-44680389(+)
ACAUGUAUGCCUUGGUUACUUGUUGAUCUACAUGUGACCUCAACAUACAUGUUC
>Tcy-Mir-7397 scaffold_7:44678005-44678060(+)
AUCUGUAUGUCUUGGUAACACAUAUAAAUAAAGGUGACCAAGACCUACAGAUUCU
>Tcy-Mir-7398-P1 scaffold_7:57252663-57252721(+)
GUGUAGAGAGGGGUGAAAAUGCUGUGCUUUGUCAGGUAAUUCUCCCUUCUCUUACUGU
>Tcy-Mir-7398-P25 scaffold_7:57284478-57284536(+)
GUUGAAAAGGGGUGAGGGUACCGUGCGCUAUUGGGUAACCCUCCCUUCUUUCACAGUG
>Tcy-Mir-7398-P3 scaffold_7:57268164-57268222(+)
GUGUAGAGGUGAGAAAAUUACUGUGUUGUGGCUUGGUAAAUUUCUCGCCUUUUACAAU
>Tcy-Mir-7398-P5 scaffold_7:57259804-57259866(+)
GUGUGGAGGUAAGGAACUAUGCCGUUUCCUCUCUCUCGGUAAAUCCUUGCCUCUCACAGUUG
>Tcy-Mir-7398-o1 scaffold_7:57272971-57273029(+)
GUGUAGAAGCAGGAAAAUAUGCCGUAUUUUUUCUUGGUAAAUCCUUGCUUCUCGCAGU
>Tcy-Mir-7398-o10 scaffold_7:57273650-57273706(+)
GUAGAGAGGGGUGAGGUUGCUGUGAUAUAUCAGGUAAUUCACCCUUCUCUCACUAA
>Tcy-Mir-7398-o11-v1 scaffold_7:57281115-57281175(+)
GUACAGAGGGUGGUGAGCAUGCUGUGGCCUCUCAAGUAUCUCACCUUUCUCUGACUGUCC
>Tcy-Mir-7398-o12-v1 scaffold_7:57283448-57283508(+)
GUGCAGAGGAUGGUGAGCAUGCUGUGGCCUCUCGAGUAUCUCACCUUUCUCUGACUGUCC
>Tcy-Mir-7398-o14 scaffold_7:57286007-57286067(+)
GUGUUGAGGUUGGGAAUAUGCCAUGCUCUCUCUUGGGUAAAUUCUCAGCCUCUCACAGUU
>Tcy-Mir-7398-o2 scaffold_7:57260372-57260434(+)
GUGUGGAGGUAAGGAACUACGCCGUGUUCUCUCUCGGUAAAUCCUUACCUCUCACUGUUGGA
>Tcy-Mir-7398-o3-v2 scaffold_7:57263147-57263205(+)
GUGUAGAGGUAAGAAAUAGGCUGUGUUCUUGGUUGGUAUAUUUCUUGCCUCUACCAAU
>Tcy-Mir-7398-o4-v2 scaffold_7:57262535-57262597(+)
UGUGUAGAGGUAAGAAAUAUGCUGUGUUCCCGAUGGGUAUAUUUCUUGCCUCUACCAAUUGA
>Tcy-Mir-7398-o5 scaffold_7:57264422-57264484(+)
GUGUAGAGGUGUGAAACAUGCUGUGUUCUCUCUACUGGUAAAUUUCUCGCCUCUCACAGUUG
>Tcy-Mir-7398-o6 scaffold_7:57269237-57269295(+)
GUGUAGAGGUGAGAAAAUUACUGUGUUGUGGCUUGGUAAAUUUCUCGCCUUUUACAAU
>Tcy-Mir-7398-o7 scaffold_7:57269615-57269681(+)
GUGUAGAGGUAAGAAAAUUACUGUGCUGUAUCUUGGUAAAUUUCUUGCCUUUCACAAUUGAGCAUC
>Tcy-Mir-7398-o8 scaffold_7:57272162-57272220(+)
GUUGAGAGGCAGGAAACGUGCCGUGUUUCCUCUGGGUAAGUUUCUUGCCUCUUACAAU
>Tcy-Mir-8-P1a scaffold_3:605484798-605484858(-)
CAUCUUACUGGGCAGCAUUGGAUGGUGUCUGUGUUUCUAAUACUGCCUGGUAAUGAUGAU
>Tcy-Mir-8-P1b scaffold_5:109430131-109430194(-)
CAUCUUACCCAGCAGUGUUUGGGUGCUGCUUGGACGUCUCUAAUACUGCCGGGUAACGAUGGA
>Tcy-Mir-8-P2a scaffold_3:605482175-605482235(-)
CAUCUUACUAGACAGUGCUGGAUUUUUGGAUGUAUUCUAACACUGUCUGGUAACGAUGUU
>Tcy-Mir-8-P2b scaffold_5:109429608-109429673(-)
CAUCUUCCAGUGCAGUGUUGGAUCGUGUAAUCGUGAAGCUUCUAACACUGUCUGGUAAAGAUGCC
>Tcy-Mir-8-P3a scaffold_3:605477706-605477767(-)
UGUCUUACCAGACAAAGUUAGAUCUCGCUAUUUCUGUCUAAUACUGUCUGGUAAUGCCAUU
>Tcy-Mir-875 scaffold_1:596990027-596990085(-)
AAUACCUCAGUUUUAUCAGGUGUUCUUUAAAAAUCACCUGGAAAUGCUGAGGUUGCGU
>Tcy-Mir-9-P1 scaffold_1:187960108-187960168(-)
UCUUUGGUUAUCUAGCUGUAUGAGUGUAUUGGUCUUCAUAAAGCUAGAUAACCGAAAGUA

```
>Tcy-Mir-9-P2 scaffold_4:368600136-368600198(-)
UCUUUGGUUAUCUAGCUGUAUGAGUGGUGUCGAGUCUUCAUAAAGCUAGAUAACCGAAAGUA
>Tcy-Mir-9-P3 scaffold_2:562602795-562602857(+)
UCUUUGGUUAUCUAGCUGUAUGAGUGUUAUUGAGCUAUCAUAAAGCUAGAUAACCGAAAGUA
>Tcy-Mir-9-P4 scaffold_1:685692577-685692636(+)
UCUUUGGUUUCCUAGCUGUGUGAGUGUCUCUGAAUCAUAAAGCUGGAGAACCGAAUGUG
>Tcy-Mir-92-P1a scaffold_3:143852581-143852640(+)
AGGUUGGGAUCAGUUGCAAUGCUGUGUCUGUCUGUAGUAUUGCACUUGUCCCGGCCUGU
>Tcy-Mir-92-P1c scaffold_7:37924763-37924828(-)
GGGUGGGGAUUUGUUGCAUUACUUGAUCUUGUGUCUGUAAGAGUAUUGCACUUGUCCCGGCCUGU
>Tcy-Mir-92-P1d scaffold_4:367378036-367378098(+)
AGGGACGGGACGUGGUGCAGUGUUGUUUUUUCCCCGCCAAUAUUGCACUCGUCCCGGCCUCC
>Tcy-Mir-92-P2a scaffold_6:129571534-129571594(+)
ACUGUUGCUAACAUGCAACUCUGUUACAUGUAAACGGGAAUUGCACUUUAGCAAUGGUGA
>Tcy-Mir-92-P2c scaffold_7:37924627-37924692(-)
CGGGUGGAUCACGAUGCAAUUUUGAUAAGUUUAGUAGGAGAAAAAUUGCACGGUAUCCAUCUGUA
>Tcy-Mir-92-P2d scaffold_4:295402343-295402403(-)
AGGCGGAGACUUGGGCAAUUGCUGAACACUGCCCUAGGCAUUGCACUUGUCUCGGUCUGA
>Tcy-Mir-96-P1 scaffold_5:182343873-182343937(-)
UUUGGCACUAGCACAUUUUUGCUUCUGUCUCUCUGCUCUGAGCAAUCAUGUGUAGUGCCAAUAU
>Tcy-Mir-96-P2 scaffold_5:182334825-182334890(-)
UUUGGCAAUGGUAGAACUCACACUGGUGAGAUAACAGAAUCCGGUGGUUCUAGACUUGCCAACUA
>Tcy-Mir-96-P3-v1 scaffold_5:182344428-182344490(-)
UAUGGCACUGGUAGAAUUCACUGUGAAAACACACUAUCAGUGAAUUACCAAAGGGCCAUAAA
>Tcy-Novel-1 scaffold_1:220905164-220905223(+)
GGGGAACCAGGUGAUUGCGUGCCUAUUGAAGACAGCAUCAACACUUCUUGUUUCUCUCU
>Tcy-Novel-10 scaffold_14513:2234-2292(-)
UGGCAGCAGGUGAUUGCUGAGGCUGAGGCUUCUUUAGGCCCCAGCUCUCACCUCCCAC
>Tcy-Novel-15 scaffold_7:74897398-74897456(+)
AUCCGGCCUGAAGACACAUUGUGUGAUUAUUUUAAACUAUGUAUUUUCAGACCAGAGU
>Tcy-Novel-16-P16 scaffold_5062:993-1052(+)
AAACACUCUGAAGCCACAUUGUUUGUAUAAUUCUUUGAACAAUGUGUCUCAGUGUGUUG
>Tcy-Novel-16-P17 scaffold_5062:2274-2334(+)
AAAGACUCUGUAGCCACAUUGUUUGUAUAAUUCUUUGAACAAUGUGUCUCAGAGUGUUGU
>Tcy-Novel-16-P18 scaffold_7:74907761-74907821(+)
UCUGGUCUGAAGACAUAUUGUUUUUAUGACUGAACAAUGUGUCUCAGAUCAGUUGCGGGU
>Tcy-Novel-16-P23 scaffold_2579:5926-5986(+)
AGACAUUCUGAGAGCGCAUUGUUUGUAUGACUCUUUGAACAAUGCAUCUCAGAAUGUUGU
>Tcy-Novel-16-P24 scaffold_7:74909254-74909314(+)
AGCCAUUCUGAAAUCAUAUUGUUUGUAUGACUCUUUGAACAAUGUGUCUCAGAAUGUUGU
>Tcy-Novel-16-P28s scaffold_4292:114-173(-)
AGACAUUCUGAACCCACAUUGUUUGUAUGACUCUUUGAACAAUGUGUCUCAGAAUGUAG
>Tcy-Novel-16-P2a scaffold_7:74898279-74898339(+)
AUACGGUCUCAAGGCACAUUGUUUCUGUGAUUGUUUGAACAAUGUGUCUCAGAUCGGAGU
>Tcy-Novel-16-P30 scaffold_2668:2531-2591(+)
AGAUAUUCUGUAAGCACAUUGUUUGUAUGACUCUUUGAACAAUGUGUCUCAGAAUAUUGU
>Tcy-Novel-2 scaffold_1:121651293-121651349(-)
AGGUUUCCUCAUCCGGGAAUUUCUUAUACUAAUGAAAUCACAGUUGAGGAAACUCG
>Tcy-Novel-3 scaffold_3:500632941-500632997(-)
AUUUCUCCCCUUUGAGCUCAGGGGGUAGAAUAGCACCUGAACUGUGGAGAGAAAAG
>Tcy-Novel-4 scaffold_2:609241009-609241064(-)
AAGUAAUGAGAAUGAUUUCUGUAGUGGAGUAAAUGGAAAUCAGUUUCAUUACUUU
>Tcy-Novel-5 scaffold_2:33213472-33213549(-)
```

ACAGGGCAAGCAGGGGCACAGGUUACCCCUCUGUGAUGAAACAGGGCAUCCCAAGCCCUGCCUCUACUUGCCU
UGGG
>Tcy-Novel-6 scaffold_2:465375712-465375771(+)
UCAAAUCCUGGAGUUACUCCUUGUCAACAACAAUAAGGAGUAACUCUGGGAUUUGAACC
>Tcy-Novel-7 scaffold_3:139359504-139359567(+)
UCUGUCCUCCAGCUGUCUUUUGAAUUCCAGAUUGUCAACUCAAGGAUAGUUGUAGGCAAGGGA
>Tcy-Novel-8 scaffold_4821:3388-3445(-)
AUGUGCAUGCAUAUAGGUACAUAUAUGCAUCUAUUUGCACGUGUGUGCAUGCAUAUG
>Tcy-Novel-9 scaffold_48478:589-645(+)
ACUCACAUUAGGCAACCUGCUUCAGCUCGCCCAGCAAGUUGGCUAAUGUGAGUUCA

Supplemental File 8

Provisional ID	:	scaffold_2_6678
Score total	:	1.7
Score for star read(s)	:	-1.3
Score for read counts	:	0
Score for mfe	:	2
Score for randfold	:	1.6
Score for cons. seed	:	-0.6
Total read count	:	55
Mature read count	:	55
Loop read count	:	0
Star read count	:	0

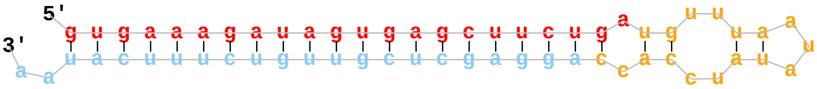

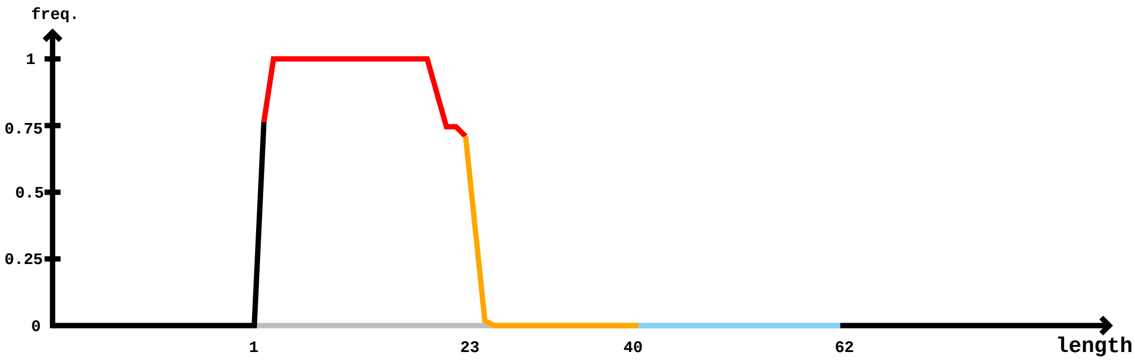

Mature Star

```
5'- gaucacggggauguuaguucgugaaagauagugagcuucugauguuuaauauauccaccaggagcucguugucuuucauaaauuaauagacgaaaguccccguacua   -3'    exp
    ....(((((((((((((((.(((((((((((((((((((((.((..((...))..)).))))))))))))))))))))).)))))))).........))))))))....     reads     mm        sample
    ....................gugaaagauagugagcuu.....................................................................    3         0         Msc
    ....................gugaaagauagugagcCu.....................................................................    1         1         Msc
    ....................gugaaagaAagugagcuu.....................................................................    1         1         Msc
    ....................gugaaagauagugUgcuu.....................................................................    1         1         Msc
    ....................gugaaGgauagugagcuu.....................................................................    1         1         Msc
    ....................gugaaGgauagugagcuuc....................................................................    1         1         Msc
    ....................gAgaaagauagugagcuuc....................................................................    1         1         Msc
    ....................Cugaaagauagugagcuuc....................................................................    1         1         Msc
    ....................gugGaagauagugagcuuc....................................................................    1         1         Msc
    ....................gugaaagaAagugagcuuc....................................................................    2         1         Msc
    ....................gCgaaagauagugagcuuc....................................................................    1         1         Msc
    ....................gugaaagauagugagcuucuCa.................................................................    1         1         Msc
    ....................gugaaagauagugagcuuUuga.................................................................    1         1         Msc
    ....................Uugaaagauagugagcuucuga.................................................................    1         1         Msc
    ....................gugaaagauagugagcuucugU.................................................................    1         1         Msc
    ....................gugaaagauagugagcCucuga.................................................................    1         1         Msc
    ....................gugaaagauagAgagcuucuga.................................................................    1         1         Msc
    ....................gugaaUgauagugagcuucuga.................................................................    1         1         Msc
    ....................gugaaagauagugCgcuucuga.................................................................    1         1         Msc
    ....................guCaaagauagugagcuucuga.................................................................    1         1         Msc
    ....................gugaaagauagugagcuucuga.................................................................    5         0         Msc
    ....................gugaaagauGgugagcuucuga.................................................................    1         1         Msc
    ....................gAgaaagauagugagcuucuga.................................................................    1         1         Msc
    ....................Cugaaagauagugagcuucuga.................................................................    1         1         Msc
    ....................gugaaagauagugagcuucugaA................................................................    1         1         Msc
    ....................gugaaagauagugagcuucugau................................................................    3         0         Msc
    ....................gugaaagauagugagcuuAugau................................................................    1         1         Msc
    ....................gugaaagauagugagcuuGugau................................................................    1         1         Msc
    ....................gugaaagauagugUgcuucugau................................................................    1         1         Msc
    .....................ugaaagauagugagcuucuga.................................................................    4         0         Msc
    .....................uUaaagauagugagcuucuga.................................................................    1         1         Msc
    .....................ugaaagauGgugagcuucuga.................................................................    1         1         Msc
    .....................ugaaagaCagugagcuucuga.................................................................    1         1         Msc
    .....................ugaaagauGgugagcuucugau................................................................    1         1         Msc
```

```
                    Mature                                  Star
gaucacggggauguuaguucgugaaagauagugagcuucugauguuuaauauauccaccaggagcucguugucuuucauaaauuaauagacgaaaaguccccguacua
.....................ugaaagauagugaAcuucugau.................................................................   1   1   Msc
.....................ugaaagauagugagcuucuCau.................................................................   1   1   Msc
.....................ugaaagauagugagcuucugau.................................................................   3   0   Msc

....................gugGaagauagugagcuucug...................................................................   1   1   Skn
....................gugaaagauagugagcGucug...................................................................   1   1   Skn
....................gugaaagauagugCgcuucugau.................................................................   1   1   Skn
....................gCgaaagauagugagcuucugaug................................................................   1   1   Skn
```

Provisional ID	:	scaffold_7_26772
Score total	:	1.6
Score for star read(s)	:	-1.3
Score for read counts	:	0
Score for mfe	:	1.9
Score for randfold	:	1.6
Score for cons. seed	:	-0.6
Total read count	:	21
Mature read count	:	21
Loop read count	:	0
Star read count	:	0

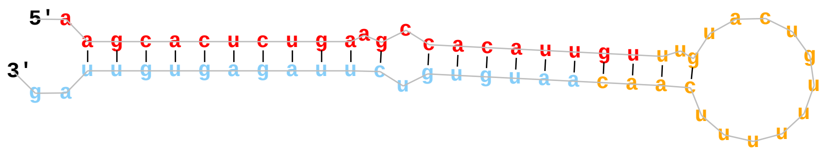

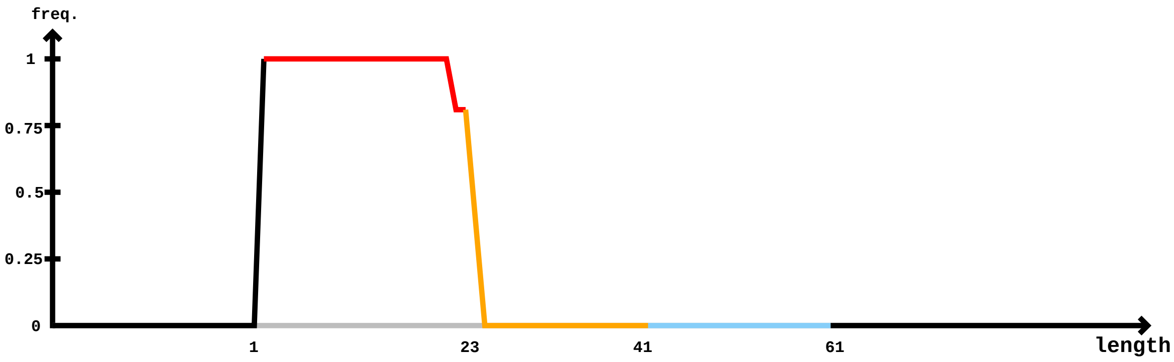

Mature **Star**

```
5'- ugagcugcugaaguaccccuaagcacucugaagccacauuguuuguacuguuuuuucaacaaugugucuuagaguguuaggggcccuuagcacccacagcaucuucugugag   -3'    exp
    .....((((((.(..(((((((.((((((((.(.(((((((((.(...........))))))))).))))))))))))))))..))))))).(((((......))))).. reads     mm       sample
    ....................aaCcacucugaagccacauu........................................................................ 1         1        Skn
    ....................aaUcacucugaagccacauu........................................................................ 1         1        Skn
    ....................aagcacucugCagccacauu........................................................................ 1         1        Skn
    ....................aagcacAcugaagccacauu........................................................................ 1         1        Skn
    ....................aagcacucugCagccacauugu...................................................................... 1         1        Skn
    ....................aagcacAcugaagccacauugu...................................................................... 1         1        Skn
    ....................aagcacucugaagccacauugu...................................................................... 3         0        Skn
    ....................aagcGcucugaagccacauugu...................................................................... 1         1        Skn
    ....................aagcacucugaagccacauAgu...................................................................... 1         1        Skn
    ....................aagcCcucugaagccacauugu...................................................................... 1         1        Skn
    ....................aagcacucugaagccaUauugu...................................................................... 1         1        Skn
    ....................aagcacucugaagccacauuCu...................................................................... 1         1        Skn
    ....................aagcaAucugaagccacauugu...................................................................... 1         1        Skn
    ....................aagcacucugaCgccacauugu...................................................................... 1         1        Skn
    ....................aagGacucugaagccacauugu...................................................................... 1         1        Skn
    ....................aagcacucugaagAcacauugu...................................................................... 1         1        Skn
    ....................aagcacucugaagccaUauuguu..................................................................... 1         1        Skn
    ....................aagcacucugaagccacauuguA..................................................................... 1         1        Skn
    ....................aagcaGucugaagccacauuguu..................................................................... 1         1        Skn
```
