# Supplementary material for: Historical RNA expression profiles from the extinct Tasmanian tiger
Source: Genome Res. 2023 Aug;33(8):1299–316. doi: 10.1101/gr.277663.123 (PMC10552650; doi:10.1101/gr.277663.123)
Supplement: Supplement 9 [file Supplemental_Figures.pdf]

Historical RNA expression profiles from the extinct Tasmanian tiger

Emilio Mármol-Sánchez[1,2*], Bastian Fromm[1,3], Nikolay Oskolkov[4], Zoé Pochon[2,5], Panagiotis Kalogeropoulos[1], Eli Eriksson[1], Inna Biryukova[1], Vaishnovi Sekar[1], Erik Ersmark[2,6], Björn Andersson[7], Love Dalén[2,6,8#*] and Marc R. Friedländer[1#*]

[1]Department of Molecular Biosciences, The Wenner-Gren Institute, Science for Life Laboratory, Stockholm University, Stockholm, Sweden. [2]Centre for Palaeogenetics, Stockholm, Sweden. [3]The Arctic University Museum of Norway, UiT, The Arctic University of Norway, Tromsø, Norway. [4]Department of Biology, National Bioinformatics Infrastructure Sweden, Science for Life Laboratory, Lund University, Lund, Sweden. [5]Department of Archaeology and Classical Studies, Stockholm University, Stockholm, Sweden. [6]Department of Bioinformatics and Genetics, Swedish Museum of Natural History, Stockholm, Sweden. [7]Department of Cell and Molecular Biology, Karolinska Institute, Stockholm, Sweden. [8]Department of Zoology, Stockholm University, Stockholm, Sweden. [#]LD and MRF contributed equally. [*]Corresponding authors: MRF, LD and EMS.

Corresponding authors:

Marc R. Friedländer: marc.friedlander@scilifelab.se

Love Dalén: love.dalen@zoologi.su.se

Emilio Mármol-Sánchez: emilio.marmol.sanchez@gmail.com

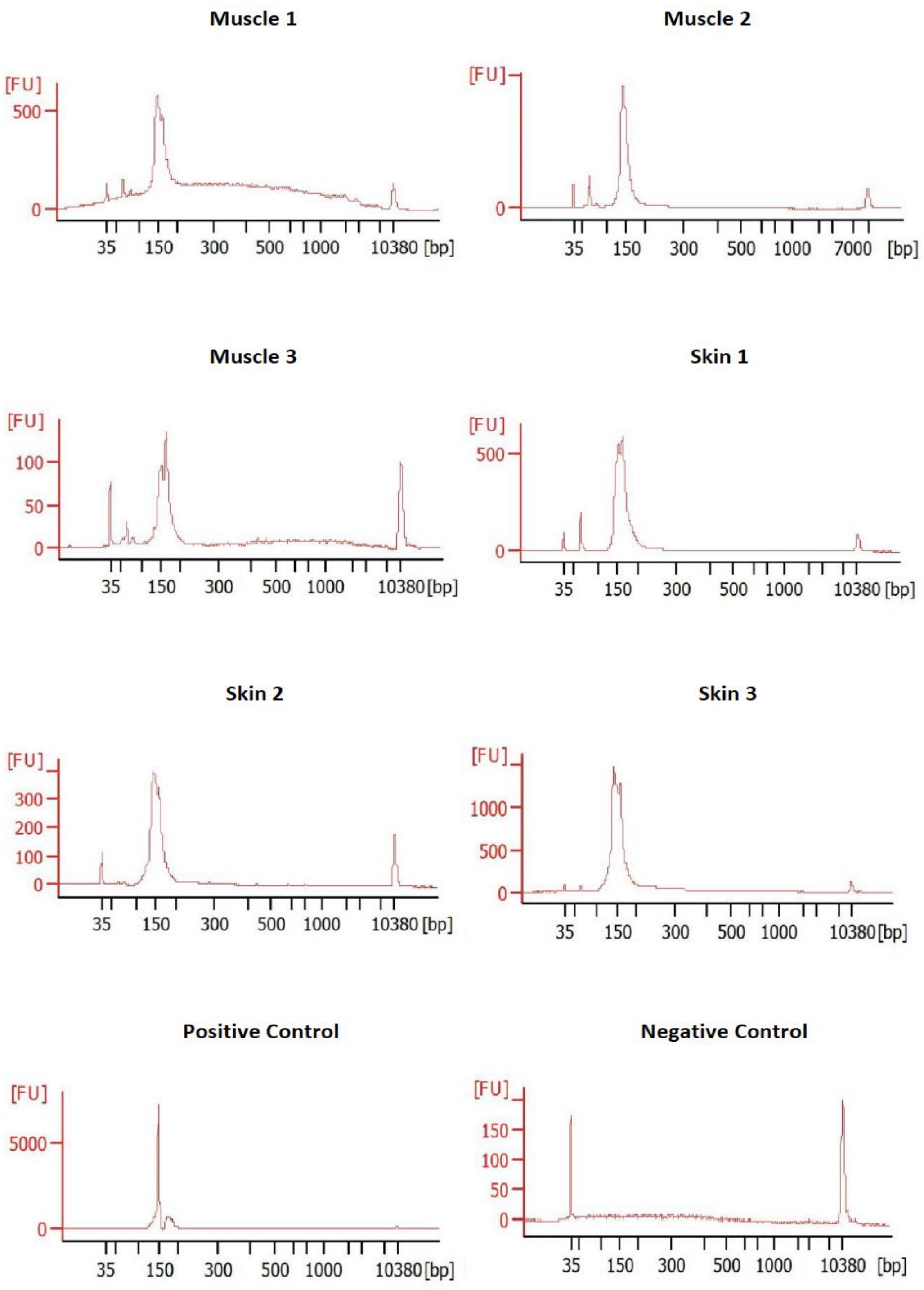

Supplemental Figure 1: Distribution of DNA fragment sizes in base pairs (bp) from sequencing libraries prepared using the NEXTflex^{TM} Small RNA-seq kit v3 protocol.

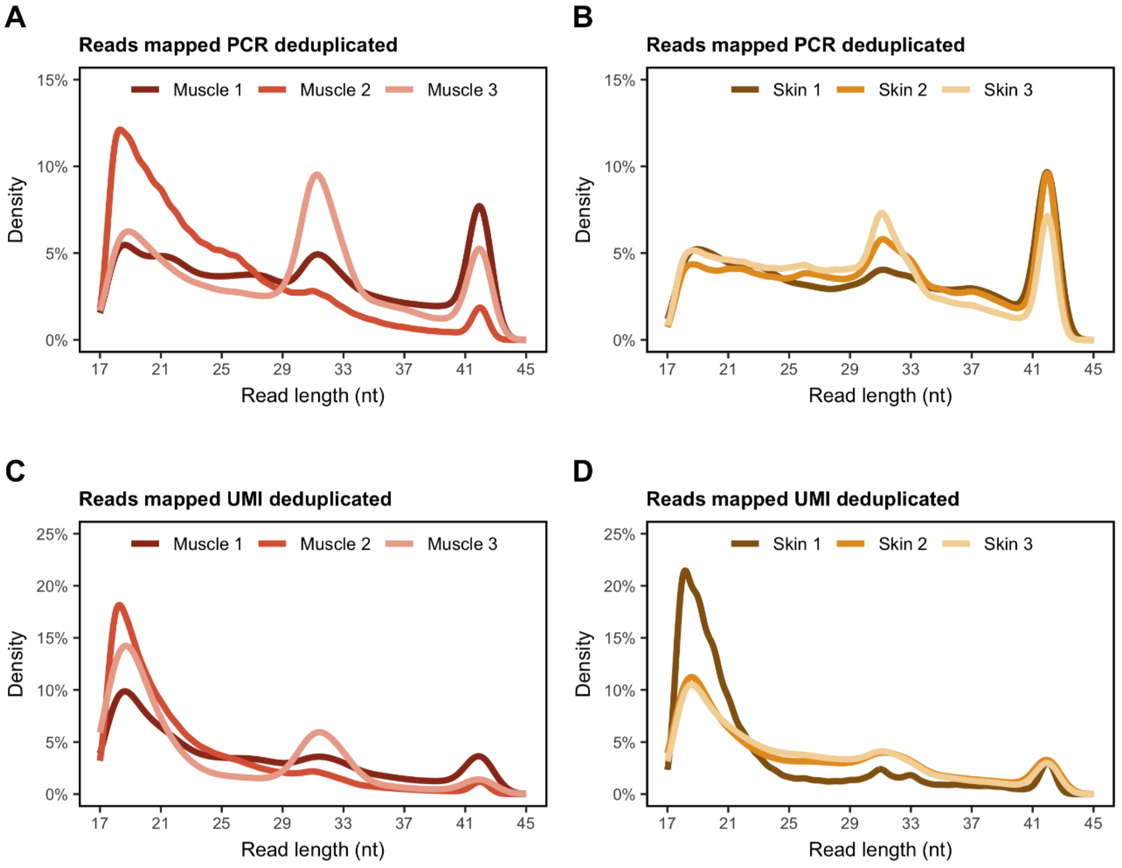

Supplemental Figure 2: Read length distribution across samples. Total reads mapped to the thylacine genome after PCR deduplication in **(A)** skeletal muscle and **(B)** skin tissues. Total reads mapped to the thylacine genome after UMI deduplication in **(C)** skeletal muscle and **(D)** skin tissues.

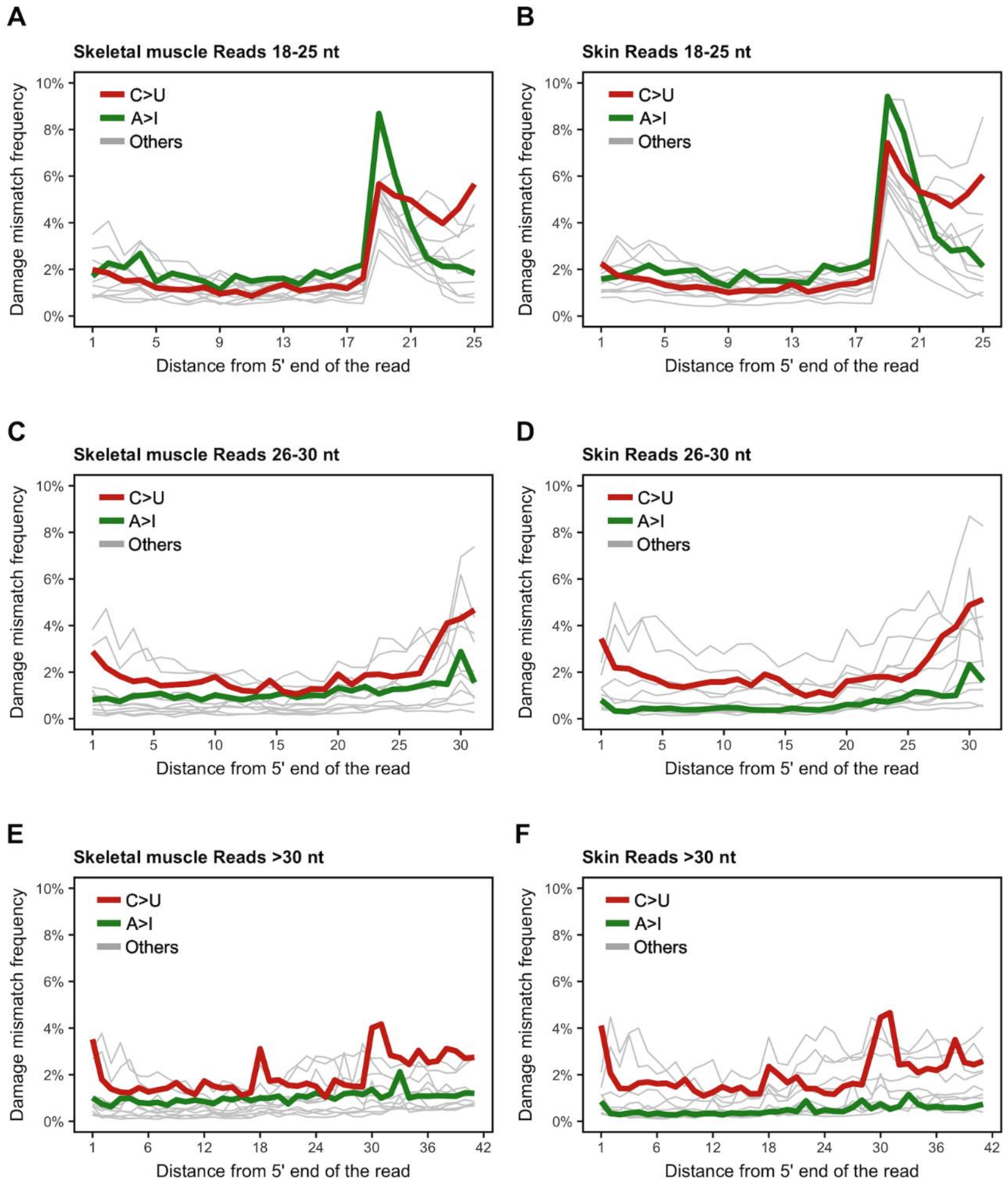

Supplemental Figure 3: Damage analyses performed on RNA reads of length **(A, B)** 18-25 nt, **(C, D)** 26-30 nt and **(E, F)** >30 nt from skeletal muscle and skin tissues, respectively. Reads were aligned to the thylacine genome assembly. Misincorporations based on cytidine deamination (C>U, in red), and adenosine deamination (A>I, in green) are shown across the full length of the reads analyzed. Other types of misincorporations are shown in grey.

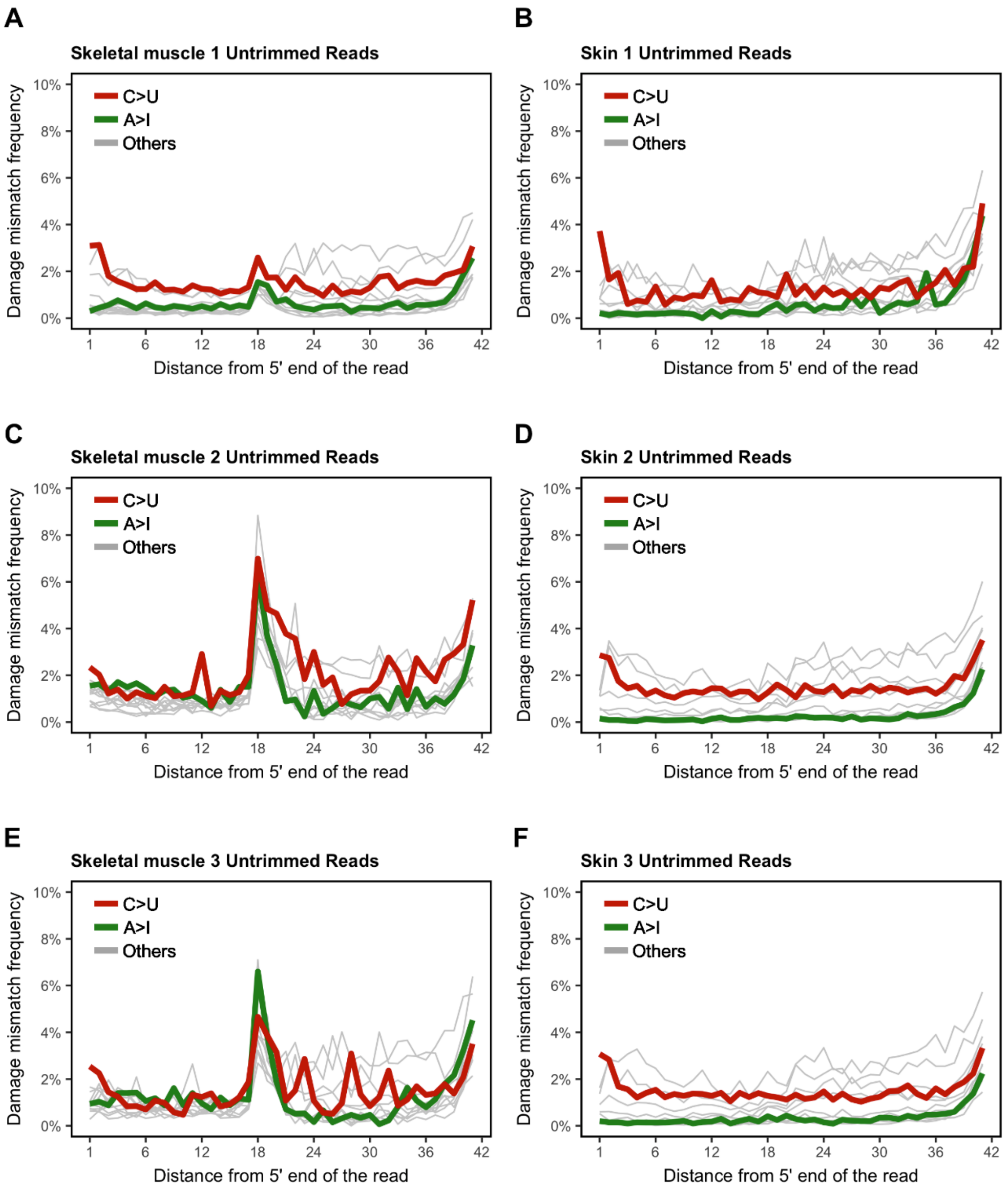

Supplemental Figure 4: Damage analyses performed on untrimmed RNA reads from skeletal muscle and skin samples. **(A)** Skeletal muscle sample 1. **(B)** Skin sample 1. **(C)** Skeletal muscle sample 2. **(D)** Skin sample 2. **(E)** Skeletal muscle sample 3. **(F)** Skin sample 3. Reads were aligned to the thylacine genome assembly. Misincorporations based on cytidine deamination (C>U, in red), and adenosine deamination (A>I, in green) are shown across the full length of the reads analyzed. Other types of misincorporations are shown in grey.

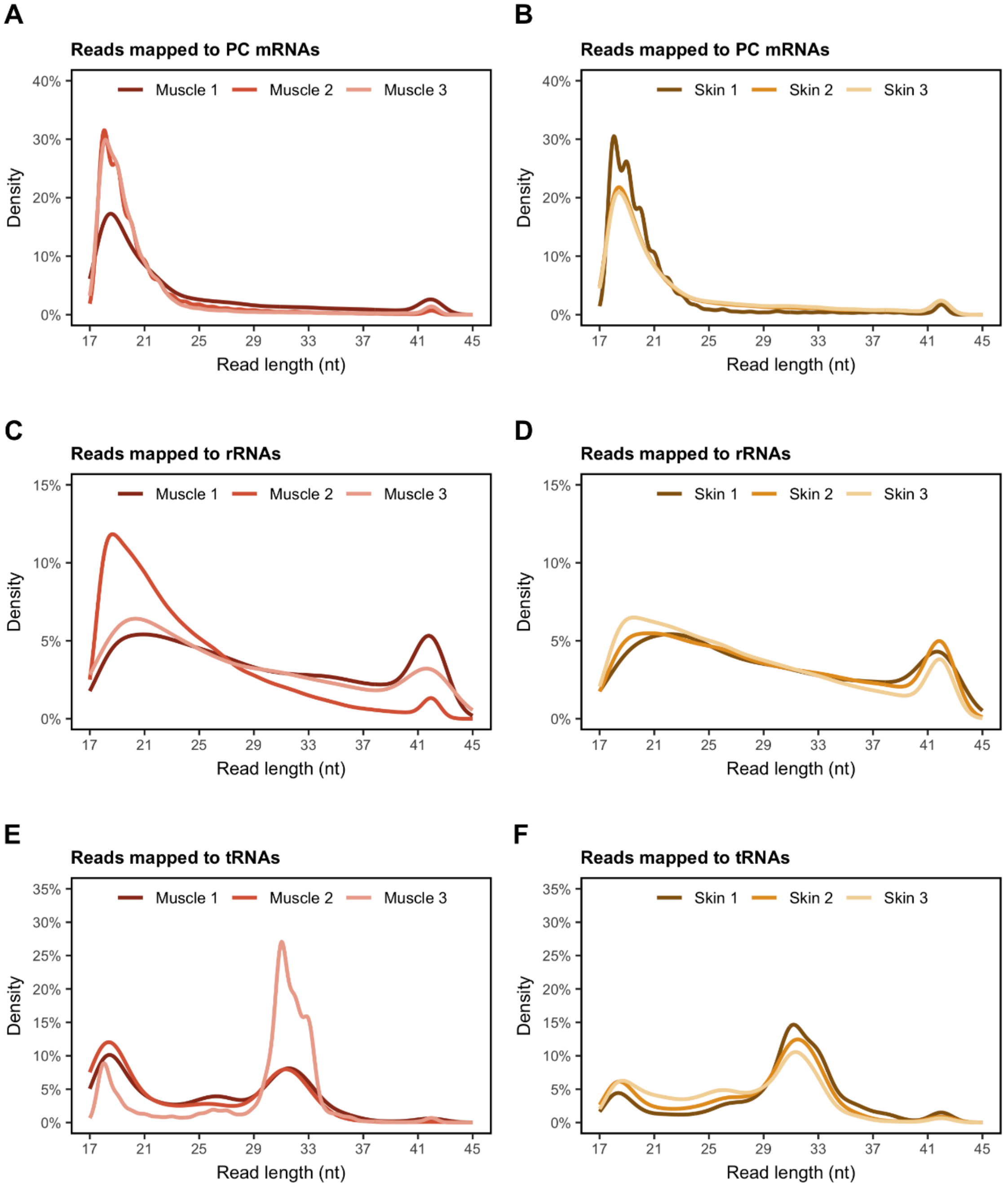

Supplemental Figure 5: Read length distribution across samples mapped to annotated genes. Total reads UMI-deduplicated and mapped to protein-coding (PC) mRNA genes in **(A)** skeletal muscle and **(B)** skin tissues. Total reads UMI-deduplicated and mapped to ribosomal RNA (rRNA) genes in **(C)** skeletal muscle and **(D)** skin tissues. Total reads UMI-deduplicated and mapped to transfer RNA (tRNA) genes in **€** skeletal muscle and **(F)** skin tissues.

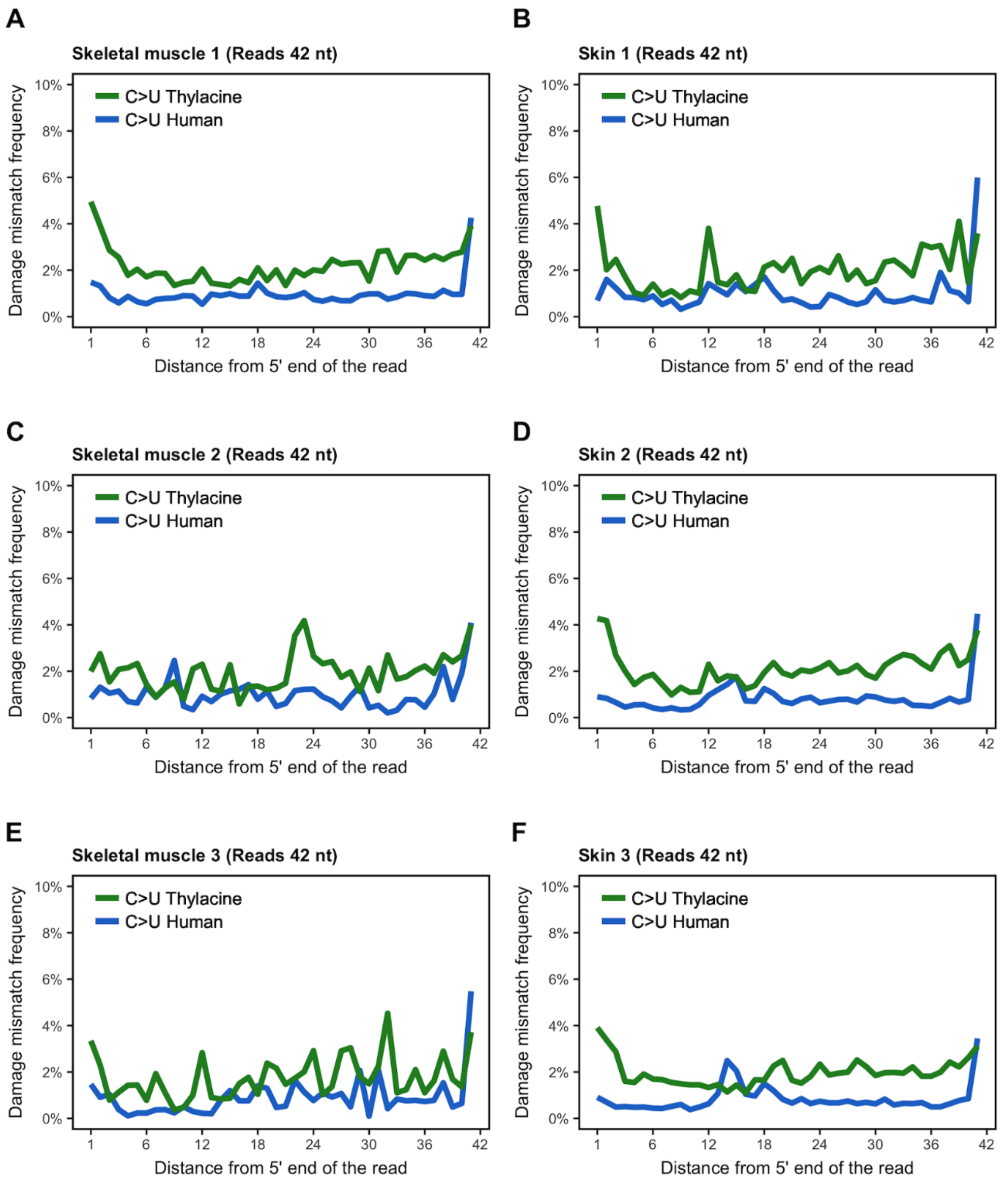

Supplemental Figure 6: Deamination (C>U) profile of trimmed RNA reads of length 42 nt mapped to both thylacine (Feigin et al. 2022), and human (GRCh37, hg19) genome assemblies (suspicious of modern human-derived contamination), compared to deamination (C>U) profile of trimmed RNA reads of length 42 nt mapped only to the thylacine assembly. **(A)** Skeletal muscle sample 1. **(B)** Skin sample 1. **(C)** Skeletal muscle sample 2. **(D)** Skin sample 2. **(E)** Skeletal muscle sample 3. **(F)** Skin sample 3.

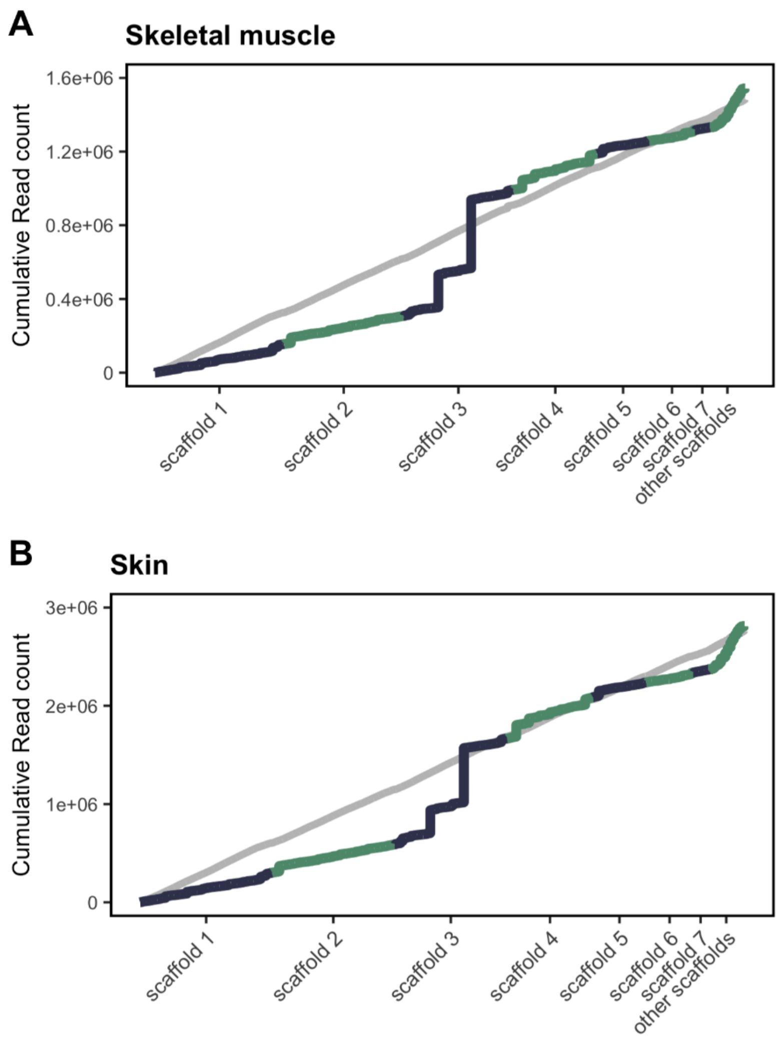

Supplemental Figure 7: Cumulative distribution of the number of RNA reads mapped to each consecutive 250 kbp window genome-wide in **(A)** skeletal muscle and **(B)** skin tissues.

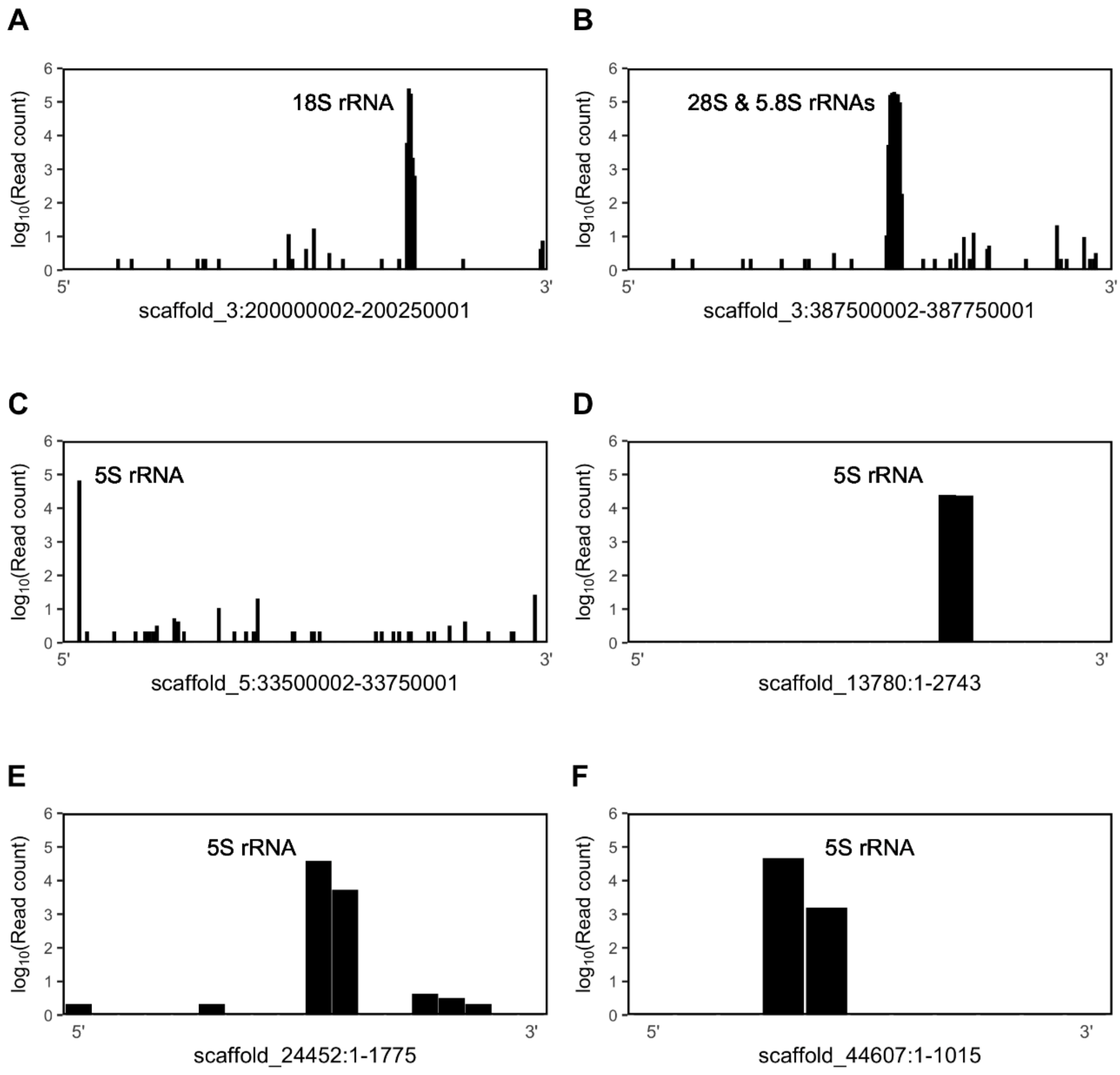

Supplemental Figure 8: Number of RNA reads mapped to genomic windows harboring gene expression hotspots compatible with the presence of the novel annotated **(A)** 18S and **(B)** 28S and 5.8S rRNA loci in the thylacine genome assembly. **(C-F)** Number of RNA reads mapped to genomic windows harboring the four annotated 5S rRNA loci in the thylacine genome assembly.

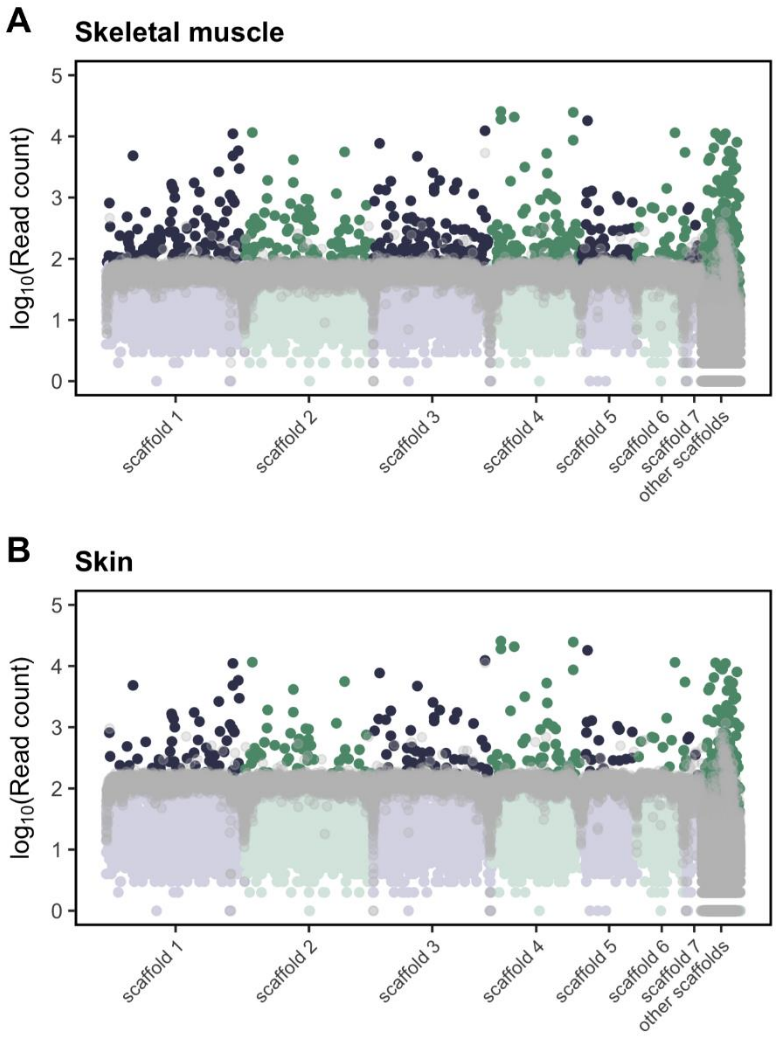

Supplemental Figure 9: Number of intergenic trimmed RNA reads mapped to each consecutive 250 Kbp window genome-wide in **(A)** skeletal muscle and **(B)** skin tissues. Thylacine DNA reads (SRR5055304) mapped to each consecutive 250 Kbp window genome-wide are in grey. Gene annotations include those provided by Feigin et al. 2022, as well as novel 28S, 18S and 5.8S rRNAs and microRNAs (N = 325) in the thylacine assembly.

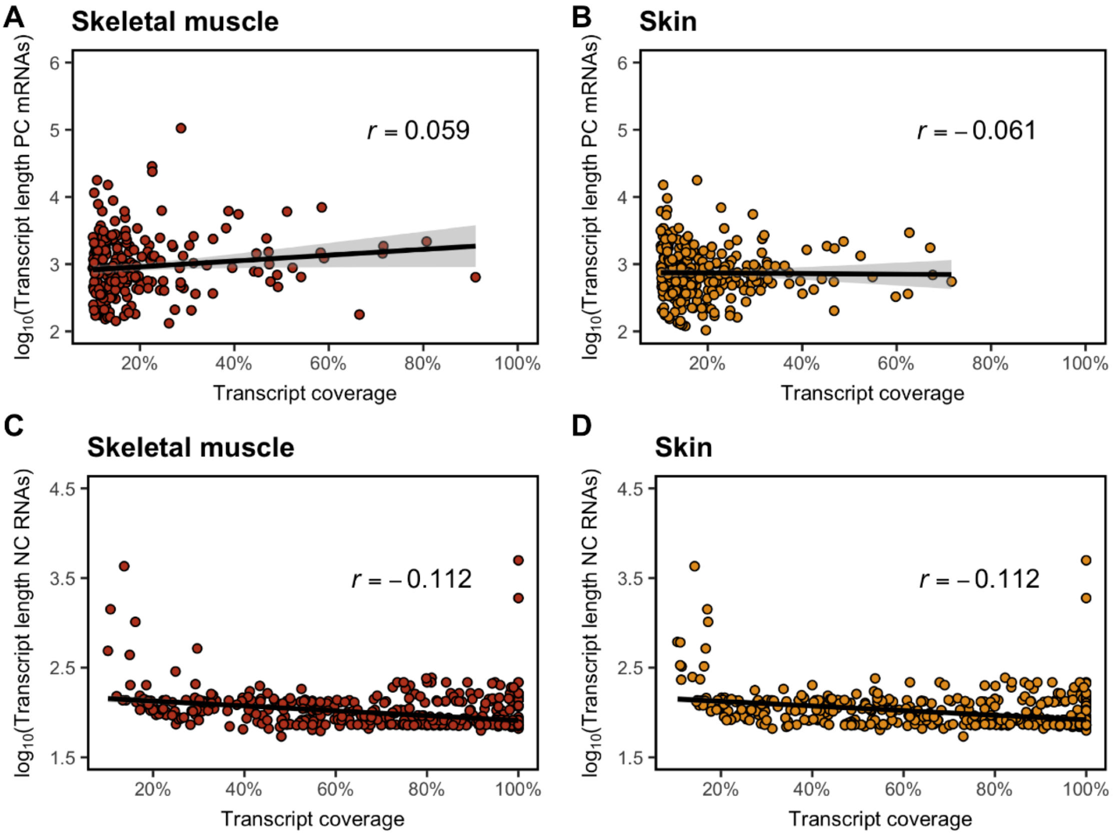

Supplemental Figure 10: Pearson's correlation coefficient between transcript length and coverage in protein-coding (PC) and noncoding (NC) RNA genes. Transcript length and the corresponding obtained coverage of each PC gene (at least one read mapped and at least 10% coverage) in **(A)** skeletal muscle (N = 236) and **(B)** skin (N = 270). Transcript length and the corresponding obtained coverage of each NC RNA gene (at least one read mapped and at least 10% coverage) in **(C)** skeletal muscle (N = 608) and **(D)** skin (N = 637).

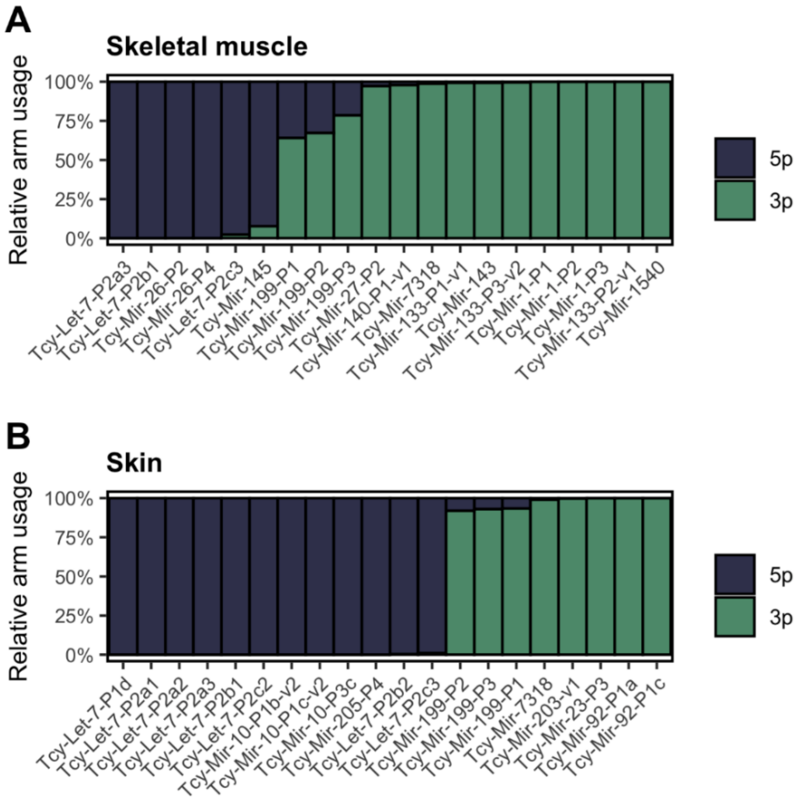

Supplemental Figure 11: Relative proportion of arm usage for each of the 20 most abundant thylacine miRNAs profiled in **(A)** skeletal muscle and **(B)** skin tissue.

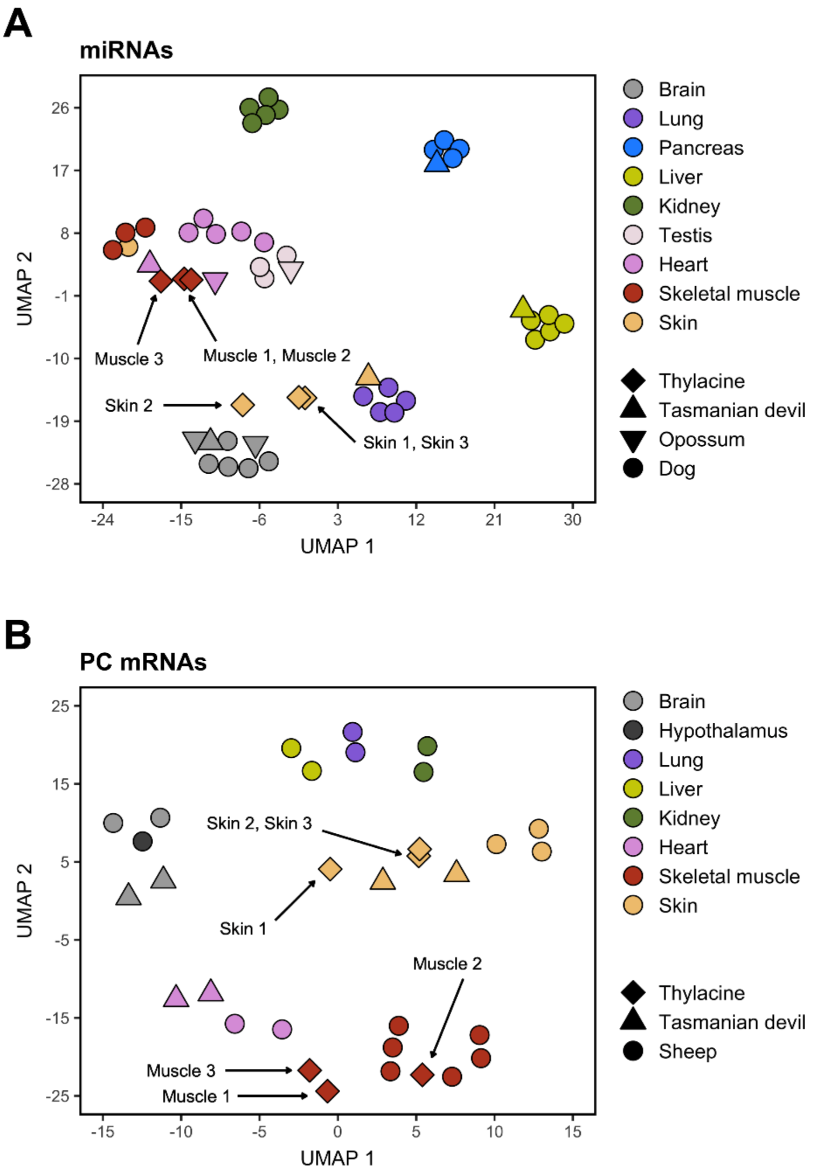

Supplemental Figure 12: (A) UMAP embedding depicting diverse tissue samples clustering belonging to dog (circular shape), Tasmanian devil (triangular shape), and opossum (inverted triangular shape) miRNA expression profiles (N = 119) available at MirGeneDB2.1 (Fromm et al. 2022), as well as miRNA profiles of each individual sample (N = 6) from thylacine skeletal muscle and skin tissues (diamond shape). **(B)** UMAP embedding depicting diverse tissue samples clustering belonging to sheep (circular shape) and Tasmanian devil (triangular shape) protein-coding mRNA expression profiles (N = 261 mRNAs), as well as protein-coding mRNA expression profiles of each individual samples (N = 6) from thylacine skeletal muscle and skin tissues (diamond shape).

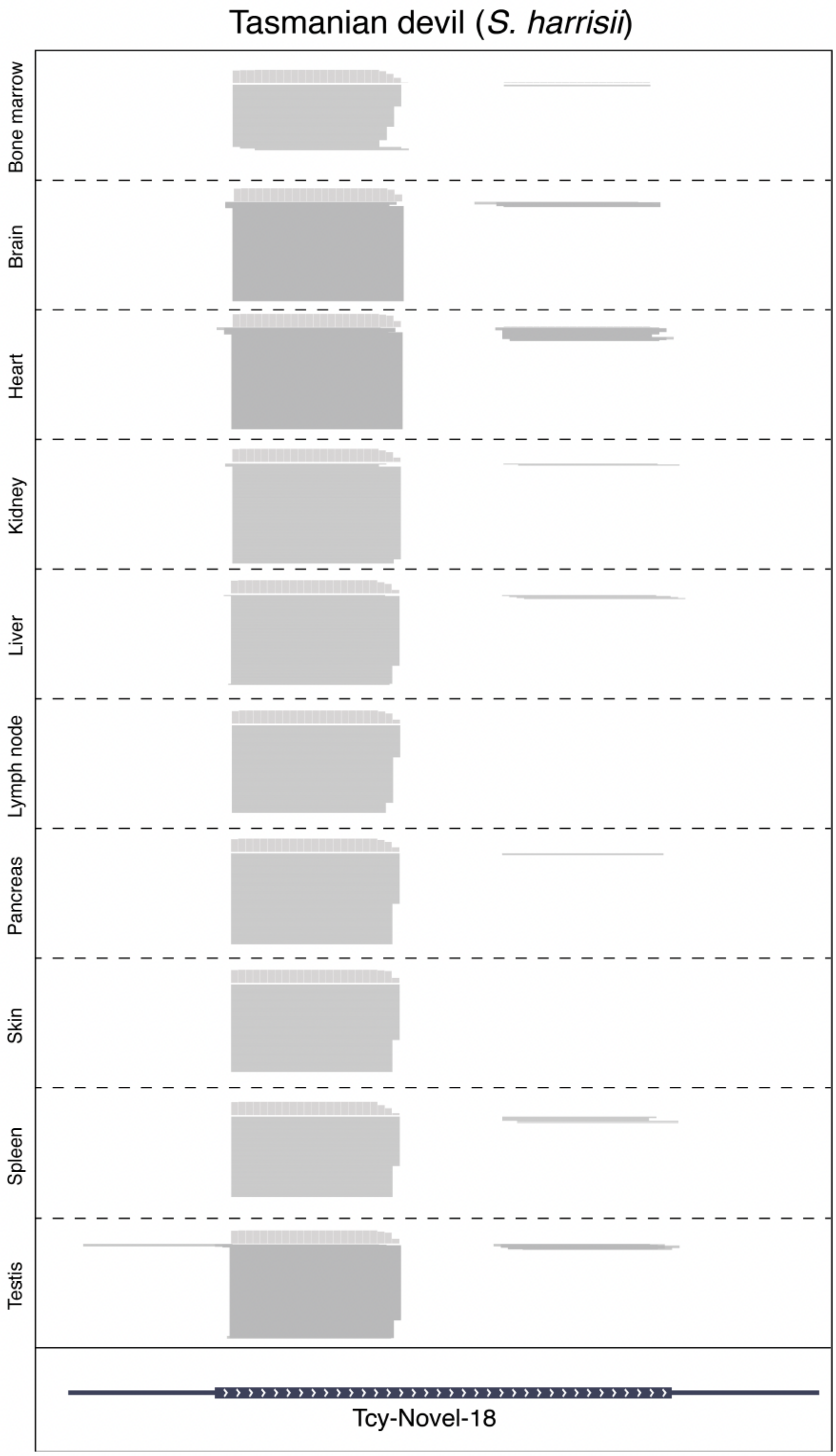

Supplemental Figure 13: Transcriptional evidence for Tcy-Novel-18 homologous miRNA locus in the Tasmanian devil genome assembly (mSarHar1.11) using a collection of small RNA-seq datasets (GSE18352) obtained from bone marrow, brain, heart, kidney, liver, lymph node, pancreas, skin, spleen, and testis tissues from the Tasmanian devil (*S. harrisii*) species.

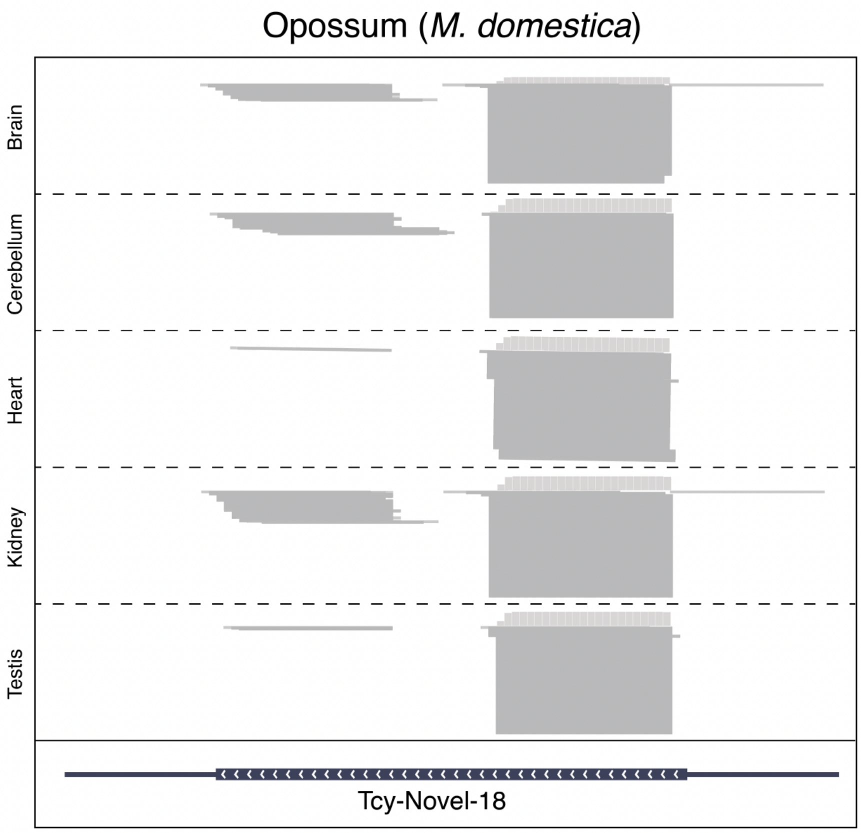

Supplemental Figure 14: Transcriptional evidence for Tcy-Novel-18 homologous miRNA locus in the opossum genome assembly (MonDom5) using a collection of small RNA-seq datasets (GSE40499) obtained from brain, cerebellum, heart, kidney and testis tissues from the opossum (*M. domestica*) species.

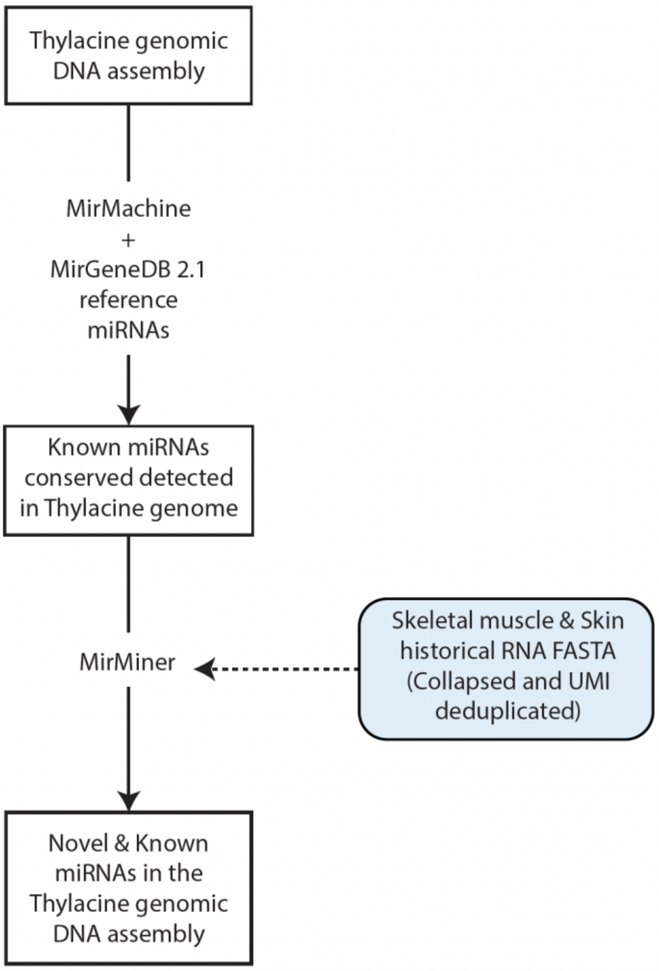

Supplemental Figure 15: miRNA annotation pipeline.

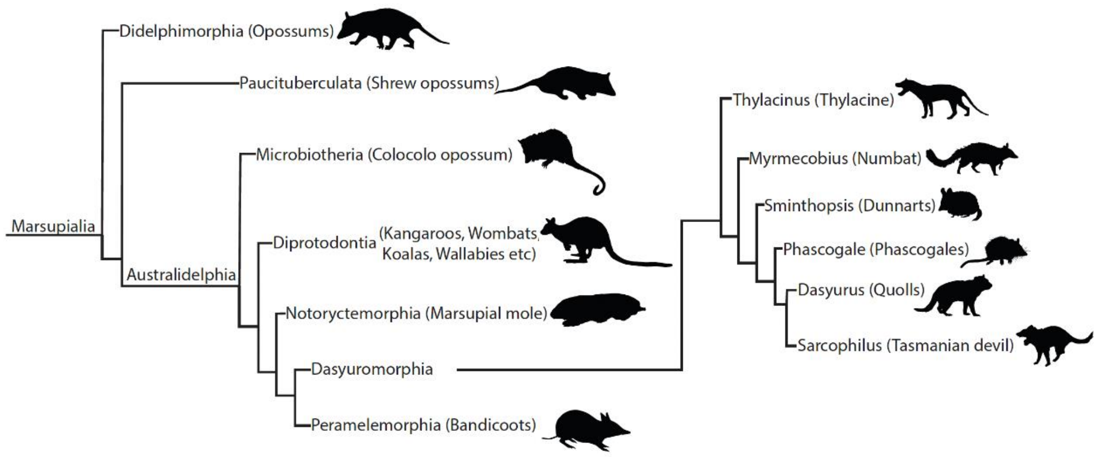

Supplemental Figure 16: Schematic representation of the evolutionary relationship among marsupial species and a detailed view of relevant species from the order *Dasyuromorphia*.
